# Development of Biphenyl-Substituted Uracil-Based Hydroxamic Acids (UBHAs) as Potent HDAC Inhibitors with Pro-Apoptotic Activity in Leukemia and Prostate Cancer Cells

**DOI:** 10.1021/acs.jmedchem.5c02737

**Published:** 2026-04-30

**Authors:** Francesco Fiorentino, Giulio Bontempi, Federica Michetti, Valeria Pecci, Emanuele Fabbrizi, Daniela Passeri, Letizia Corsetti, Valentina Farini, Fabrizio Casano, Antimo Gioiello, Antonella Di Sotto, Roberto Pellicciari, Donatella Del Bufalo, Daniela Trisciuoglio, Simona Nanni, Raffaele Strippoli, Antonello Mai, Dante Rotili

**Affiliations:** † Department of Biochemical Sciences, 9311 Sapienza University of Rome, Piazzale Aldo 5, 00185 Rome, Italy; ‡ Department of Molecular Medicine, Sapienza University of Rome, Piazzale Aldo Moro 5, 00185 Rome, Italy; § Gene Expression Laboratory, National Institute for Infectious Diseases, Lazzaro Spallanzani IRCCS, Via Portuense, 292, 00149 Rome, Italy; ∥ Department of Translational Medicine and Surgery, Università Cattolica del Sacro Cuore, Largo Francesco Vito, 1, 00168 Rome, Italy; ⊥ Department of Drug Chemistry and Technologies, Sapienza University of Rome, Piazzale Aldo 5, 00185 Rome, Italy; # 299875 TES Pharma S.r.l., Via P. Togliatti 20, Corciano, 06073 Perugia, Italy; ∇ Department of Physiology and Pharmacology “V. Erspamer”, Sapienza University of Rome, Piazzale Aldo Moro 5, 00185 Rome, Italy; ○ Preclinical Models and New Therapeutic Agents Unit, 18658 IRCCS-Regina Elena National Cancer Institute, Rome 00144, Italy; ◆ Department of Pharmaceutical Sciences, University of Perugia, Via del Liceo 1, 06122 Perugia, Italy; ¶ Institute of Molecular Biology and Pathology, 9327 National Research Council (CNR), Via degli Apuli, 4, Rome 00185, Italy; ⋈ Fondazione “Policlinico Universitario A. Gemelli IRCCS”, Largo Francesco Vito, 1, 00168 Rome, Italy; ⧓ Department of Science, Roma Tre University, Viale Marconi 446, 00146 Rome, Italy; ⧖ Biostructures and Biosystems National Institute (INBB), Via dei Carpegna 19, 00165 Rome, Italy

## Abstract

Histone deacetylases (HDACs) regulate transcription by removing acetyl groups from lysines, and their dysregulation promotes cancer. Clinically approved HDAC inhibitors show limited isoform selectivity, toxicity, and modest efficacy in solid tumors. We therefore designed and synthesized uracil-based hydroxamic acids (UBHAs) bearing systematic cap group and linker modifications. Several compounds achieved nanomolar inhibition, particularly against HDAC6, and reduced activity toward class I isoforms. Structure–activity relationships highlight that *para*-substituted phenyl moieties and four-carbon linkers enhance potency. Compounds **14a** and **14b** emerged as lead candidates, reducing cancer cell viability at submicromolar doses while sparing noncancerous cells. In U937 cells, both promoted cell-cycle arrest, apoptosis, and H3K9 and α-tubulin acetylation, alongside modulation of apoptosis-related genes and microRNAs. In prostate cancer models, **14a** inhibited AR^–^ and AR^+^ cell proliferation, enhanced histone and tubulin acetylation, upregulated p21, and downregulated Bcl-2. These findings identify biphenyl-substituted UBHAs as promising therapeutics and probes to dissect HDAC biology.

## Graphical abstract

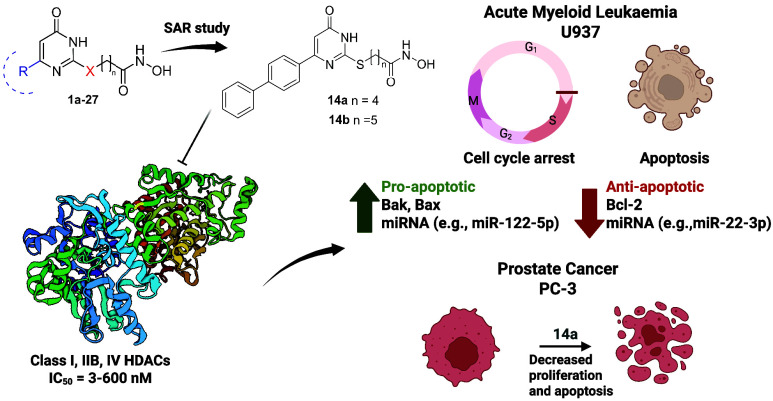

## 1 Introduction

The dynamic regulation of chromatin structure through reversible post-translational modifications is a cornerstone of eukaryotic gene expression and cellular function modulation. Among the most influential of these modifications there is lysine acetylation, a process tightly controlled by the opposing actions of histone acetyltransferases (HATs) and histone deacetylases (HDACs). Specifically, HDACs catalyze the removal of acetyl groups from the ε-amino group of lysine residues in both histone and nonhistone proteins. Their activity on histones leads to chromatin condensation, thereby resulting in transcriptional repression.^1–3^ Among nonhistone targets, HDACs may deacetylate key factors such as the tumor suppressor p53, cell cycle kinases including CDK1–9, and the essential microtubule component α-tubulin.^4–6^ Consequently, aberrant expression or activity of HDACs is linked to the alteration of multiple key cancer-related pathways. HDACs may alter cell cycle progression by repressing cyclin-dependent kinase (CDK) inhibitors like p21 and p27, leading to uncontrolled proliferation.^7^ They also may promote resistance to apoptosis through modulation of the Bcl-2 family^8,9^ and DNA damage tolerance by inactivating repair factors.^10^ HDAC aberrant activity is also linked to dysregulation of autophagy,^11^ angiogenesis, and epithelial–mesenchymal transition (EMT), thereby promoting metastasis.^12,13^ Hence, HDACs are linked to the onset and progression of various cancer types, where they contribute to the repression of tumor suppressor genes and support oncogenic signaling pathways.^12,14^

Beyond their classical roles in modifying histones and various nonhistone proteins, HDACs have recently been recognized as regulators of noncoding RNA landscapes, particularly microRNAs (miRNAs). Many miRNAs serve as critical checkpoints in apoptosis by targeting both pro-apoptotic and antiapoptotic genes.^15,16^ Aberrant HDAC function can silence tumor-suppressive miRNAs through chromatin remodeling, thereby sustaining pro-survival signals and resistance to cell death.^17^ For example, HDAC1 and HDAC3 represses the transcription of miR-769-5p/3p in mesothelium and gastric cancer respectively, promoting EMT and tumor cell viability,^18,19^ while HDAC6 has been implicated in glioblastoma development by modulating networks that link long noncoding RNAs, including miRNAs, and mRNAs, ultimately affecting cell proliferation and fate decisions.^20^ In line with these findings, pharmacological inhibition of HDACs has been shown to reprogram miRNA expression profiles in various cancer types, tilting the balance toward apoptosis by upregulating pro-apoptotic miRNAs and downregulating those promoting cell survival.^18,21–24^

Given these critical roles, HDACs have become prominent targets in the search for epigenetic cancer therapies. To this end, HDAC inhibitors (HDACi) aim to restore the balance of protein acetylation, reactivate genes silenced in cancer, and disrupt networks that sustain tumor growth. HDACi have demonstrated diverse anticancer activities, including arresting cell division, inducing apoptosis, promoting differentiation, and inhibiting the formation of new blood vessels.^1–3^ Several studies have shown that HDACi can reverse epigenetic aberrations and suppress tumor progression through mechanisms such as stimulating extrinsic and intrinsic apoptotic pathways, generating reactive oxygen species (ROS), enhancing the stability and activity of p53, causing cell cycle blockade, triggering autophagy, disrupting angiogenic signaling via factors like HIF-1α and VEGF, and influencing immune responses.^3,25,26^

Structurally, HDACi share a modular architecture composed of four key elements: a zinc-binding group (ZBG), typically a 2′-aminoanilide or a hydroxamate moiety, which chelates the catalytic Zn^2+^ ion within the HDAC active site; a hydrophobic linker that mimics the lysine side chain and extends into the enzyme’s catalytic channel; a surface-interacting cap group that engages residues at the entrance of the active site; and a connecting unit (CU) acting as a bridge between the cap and the hydrophobic linker. Several chemical classes have been developed, including hydroxamic acids, benzamides, cyclic peptides, and short-chain fatty acids, each offering distinct selectivity profiles and pharmacological behavior.^27,28^

To date, five HDACi, including the hydroxamic acids vorinostat (SAHA), belinostat, panobinostat, and givinostat, and the peptide romidepsin (Figure 1), have been approved for clinical use by regulatory agencies such as the U.S. Food and Drug Administration (FDA) and the European Medicines Agency (EMA). These agents are primarily indicated for the treatment of hematologic malignancies, while givinostat has received approval for Duchenne muscular dystrophy, which highlights the growing interest in HDAC inhibition beyond oncology. Additionally, the benzamide derivatives entinostat and tucidinostat (Figure 1) have been approved by the Chinese National Medical Products Administration (NMPA) for the treatment of both hematologic cancers and certain solid tumors.^26^

**Figure 1:**
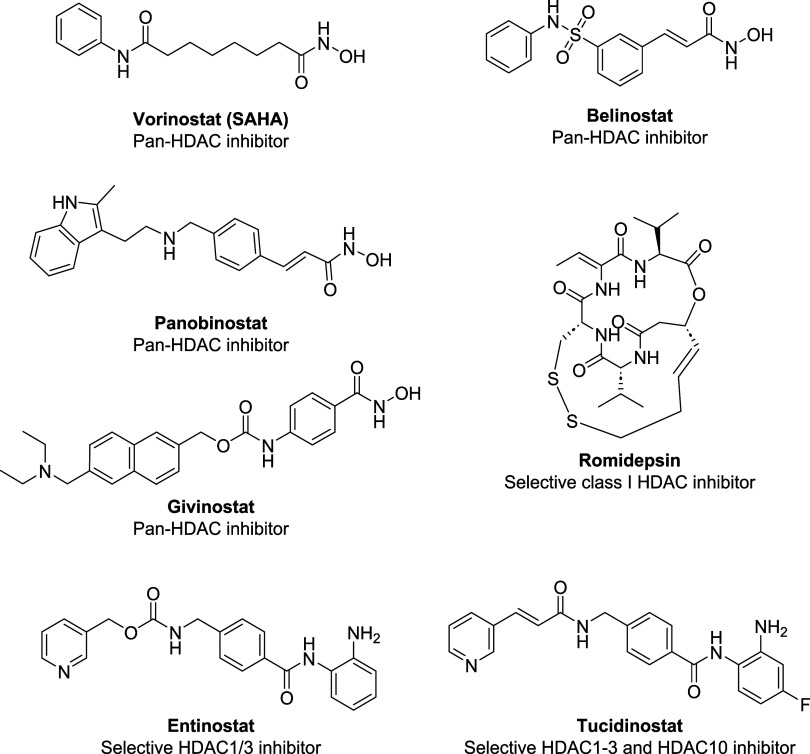
Chemical structures of approved HDACi.

Despite the approval of several HDACi for clinical use, their therapeutic application remains constrained by a few challenges. As demonstrated by currently approved therapies, HDACi are predominantly used in hematologic malignancies and have shown only limited efficacy as single agents in solid tumors. One of the most significant issues is the lack of isoform selectivity in many HDACi, which contributes to dose-limiting toxicities such as fatigue, gastrointestinal symptoms, hematologic suppression, and cardiac effects, because these agents often inhibit multiple HDAC family members in both malignant and normal tissues.^14,26,29,30^ Additionally, tumor heterogeneity and adaptive changes in HDAC expression can lead to the emergence of drug resistance, with cross-resistance frequently observed among different HDACi.^14,31^ While newer generations of HDACi are being designed to target specific isoforms or classes, thereby aiming to reduce these side effects, most clinically approved agents remain pan-inhibitors.^26^ These factors collectively limit the efficacy of HDACi, particularly in solid tumors, and underscore the need for new inhibitors with improved selectivity and resistance profiles.

In response to the continuing need for more effective HDACi for cancer treatment, our research group has devoted the past 20 years to developing a wide range of HDACi based on various chemical scaffolds.^32–51^ Among these, we designed uracil-based hydroxamic acids (UBHAs), featuring an uracil core that serves as the CU between an aromatic cap group and a short aliphatic chain functioning as the hydrophobic linker. These compounds demonstrated promising activity in cell-based assays, particularly in the context of acute myeloid leukemia (AML)^36,40^ and in cancer stem cells derived from sarcoma, colorectal cancer, and glioblastoma.^42,46,48^ However, despite their potential, this class of compounds remains underexplored from a medicinal chemistry perspective, and the impact of various substituents on the phenyl or benzyl moiety at the C6 position, which constitutes the cap group, has not been systematically investigated. Moreover, their application has thus far been largely restricted to studies in blood tumors and cancer stem cells, and they have not been thoroughly evaluated in other cancer types, such as prostate cancer, where HDACs are known to play a pivotal role.^52^ Therefore, starting from the previously reported lead compounds **1a**, **1b**, and **2**,^36^ we developed a new series of UBHAs featuring diverse substituents at the C6 position and linkers of varying length and chemical composition. The integration of medicinal chemistry optimization, biochemical characterization, and cellular assays across both hematologic and solid tumor cell lines led to the identification of two compounds, **14a** and **14b**, which exhibited nanomolar to submicromolar inhibition of HDAC1–3, 8, 10, 11, with preferential low-nanomolar inhibition of HDAC6. These compounds were found to be highly effective in inhibiting proliferation and inducing apoptosis in both hematological and solid tumor cell lines, including prostate cancer, through modulation of nuclear and cytoplasmic HDAC targets and the regulation of key apoptosis-related genes and microRNAs.

## 2 Results and Discussion

### 2.1 Chemistry

The synthesis of final compounds **1a**–**26** is illustrated in Scheme 1. UBHAs **1a**–**26** were prepared starting from the appropriate β-oxoesters, which were either commercially available (**28**–**39**, **41**–**50**, **52**) or obtained as previously reported (**40**,^53^ **51**,^54^ **53** ^54^). These β-oxoesters underwent a condensation reaction with thiourea in the presence of sodium ethoxide in ethanol under reflux conditions, affording the corresponding 6-substituted 2-thiouracil derivatives **54**,^35^ **55**,^35^ **56**–**58**, **59**,^55^ **60**,^37^ **61**,^37^ **62**–**66**, **67**,^40,49^ **68**, **69**,^49^ **70**, **71**–**73**,^56^ **74**, **75**,^57^ **76**, **77**,^54^ **78**,^58^ **79**.^54,58^ Intermediates **54**–**79** were subsequently treated with either ethyl 5-bromovalerate (**80a**, **82a**–**96a**) or ethyl 6-bromohexanoate (**81**, **82b**–**96b**, **97**–**105**) at room temperature in the presence of anhydrous potassium carbonate, affording the ethyl esters **80a**,**b**,^36^ **81**,^36^ **82a**,**b**–**84a**,**b**, **85a**,^55^ **85b**, **86a**,^37^ **86b**, **87a**,^37^ **87b**, **88a**,**b**–**92a**,**b**, **93a**,^40^ **93b**,^49^ **94a**,**b**, **95a**,**b**,^49^ **96a**,**b**, **97**–**105**. These esters were then hydrolyzed with a 2 N potassium hydroxide solution in ethanol at room temperature to yield the corresponding carboxylic acids **106a**,**b**,^36^ **107**,^36^ **108a**,**b**–**110a**,**b**, **111a**,^55^ **111b**, **112a**,^37^ **112b**, **113a**,^37^ **113b**, **114a**,**b**–**118a**,**b**, **119a**,^40^ **119b**,^49^ **120a**,**b**, **121a**,**b**,^49^ **122a**,**b**, **123**–**131**. Finally, sequential treatment of the carboxylic acids with (i) ethyl chloroformate and triethylamine in anhydrous THF, (ii) *O*-(2-methoxy-2-propyl)­hydroxylamine in anhydrous THF, and (iii) Amberlyst 15 ion-exchange resin in methanol at room temperature furnished the target hydroxamic acids **1a**,**b**,^36^ **2**,^36^ **3a**,**b**–**5a**,**b**, **6a**,^55^ **6b**, **7a**,^37^ **7b**, **8a**,^37^ **8b**, **9a**,**b**–**13a**,**b**, **14a**,^40,48^ **14b**,^49^ **15a**,**b**, **16a**,**b**,^49^ **17a**,**b**, **18**–**26**.

**Scheme 1:**
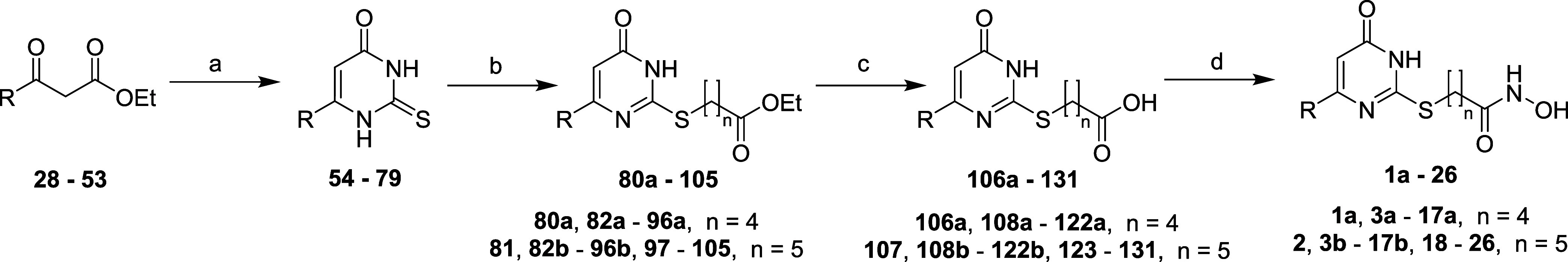
a Reagents and conditions: (a) Sodium metal, anhydrous ethanol, reflux, 12–20 h (yield = 45–68%); (b) ethyl 5-bromovalerate/ethyl 6-bromohexanoate, anhydrous potassium carbonate, anhydrous DMF, r.t., 0.5–1.5 h (yield = 51–78%); (c) 2 N potassium hydroxide, ethanol, r.t., 15–20 h (yield = 84–98%); (d) (i) ethyl chloroformate, triethylamine, anhydrous THF, 0 °C to r.t., 10 min, (ii) *O*-(2-methoxy-2-propyl)­hydroxylamine, 0 °C to r.t., 15 min–3 h, (iii) Amberlyst 15, MeOH, r.t., 0.5–2 h (yield = 45–67%).

The synthesis of compound **27** is depicted in the Scheme 2. Pyrimidinone derivative **27** was prepared starting from the commercially available β-oxoester **41**, which was initially treated with ammonium hydroxide at 120 °C to afford the β-enaminoamide derivative **132**.^40^ This intermediate then underwent a condensation reaction with diethyl pimelate in the presence of sodium ethoxide in ethanol under reflux, yielding 6-(4-([1,1′-biphenyl]-4-yl)-6-oxo-1,6-dihydropyrimidin-2-yl)­hexanoic acid **133**.^40^ Sequential treatment of compound **133** with (i) ethyl chloroformate and triethylamine in anhydrous THF, (ii) *O*-(2-methoxy-2-propyl)­hydroxylamine in anhydrous THF, and (iii) Amberlyst 15 ion-exchange resin in methanol at room temperature furnished final compound **27**.

**Scheme 2:**
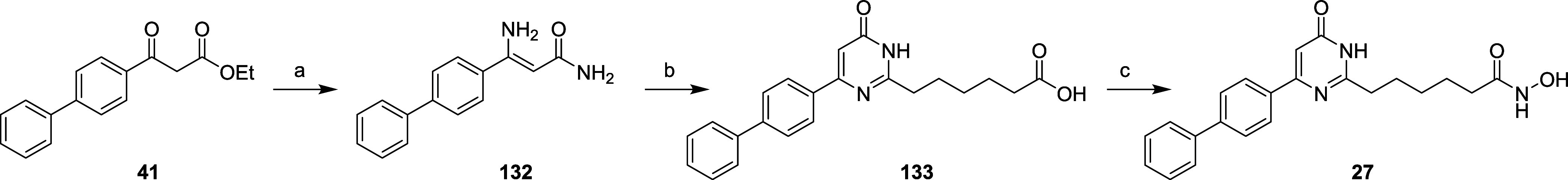
a Reagents and conditions: (a) NH_4_OH 28%, 120 °C, 2 h (yield = 66%); (b) diethyl pimelate, EtONa, EtOH, reflux, 6 h (yield = 67%); (c) (i) ethyl chloroformate, triethylamine, anhydrous THF, 0 °C to r.t., 10 min, (ii) *O*-(2-methoxy-2-propyl)­hydroxylamine, 0 °C to r.t., 1 h, (iii) Amberlyst 15, MeOH, 1.5h, r.t. (yield = 48%).

Chemical-physical data of the newly synthesized final compounds **3a**,**b**–**13a**,**b**, **15a**,**b**, **17a**,**b**–**27** are reported in the Experimental Section. Elemental analyses for final compounds **3a**,**b**–**13a**,**b**, **15a**,**b**, **17a**,**b**–**27** are reported in Table S1 (Supporting Information). High-performance liquid chromatography (HPLC) traces for final compounds **8a**, **14a**, **14b**, **16a**, and **27** are reported in are reported in Supporting Information Figures S1–S5.

### 2.2 HDAC Inhibition and Structure–Activity Relationship Evaluation

Starting from lead compounds **1a**, **1b**, and **2**, which feature either phenyl (**1a**, **1b**) or benzyl (**2**) caps at the C6 position of the uracil core, we designed an extensive series of UBHAs to explore how different substituents influence the inhibitory activity (Figure 2A). In derivatives **3a**–**26**, the thiouracil core and the hydroxamate zinc-binding group were retained, while systematic variations were introduced both in the cap group at C6 and in the length of the linker, which consists of either four or five methylene units. Specifically, compounds **3a** to **14b** incorporate a range of substituted phenyl groups, chosen to explore diverse steric and electronic properties. These substituents include small methyl groups positioned at the *ortho*, *meta*, or *para* positions in compounds **3a** to **5b**, as well as electron-withdrawing groups such as *ortho*, *meta*, or *para* chloro substituents in compounds **6a** to **8b**, and *meta* or *para* fluoro in **9a** to **10b**. Additionally, *meta* or *para* methoxy groups were introduced in compounds **11a** to **12b** (Figure 2A).

**Figure 2:**
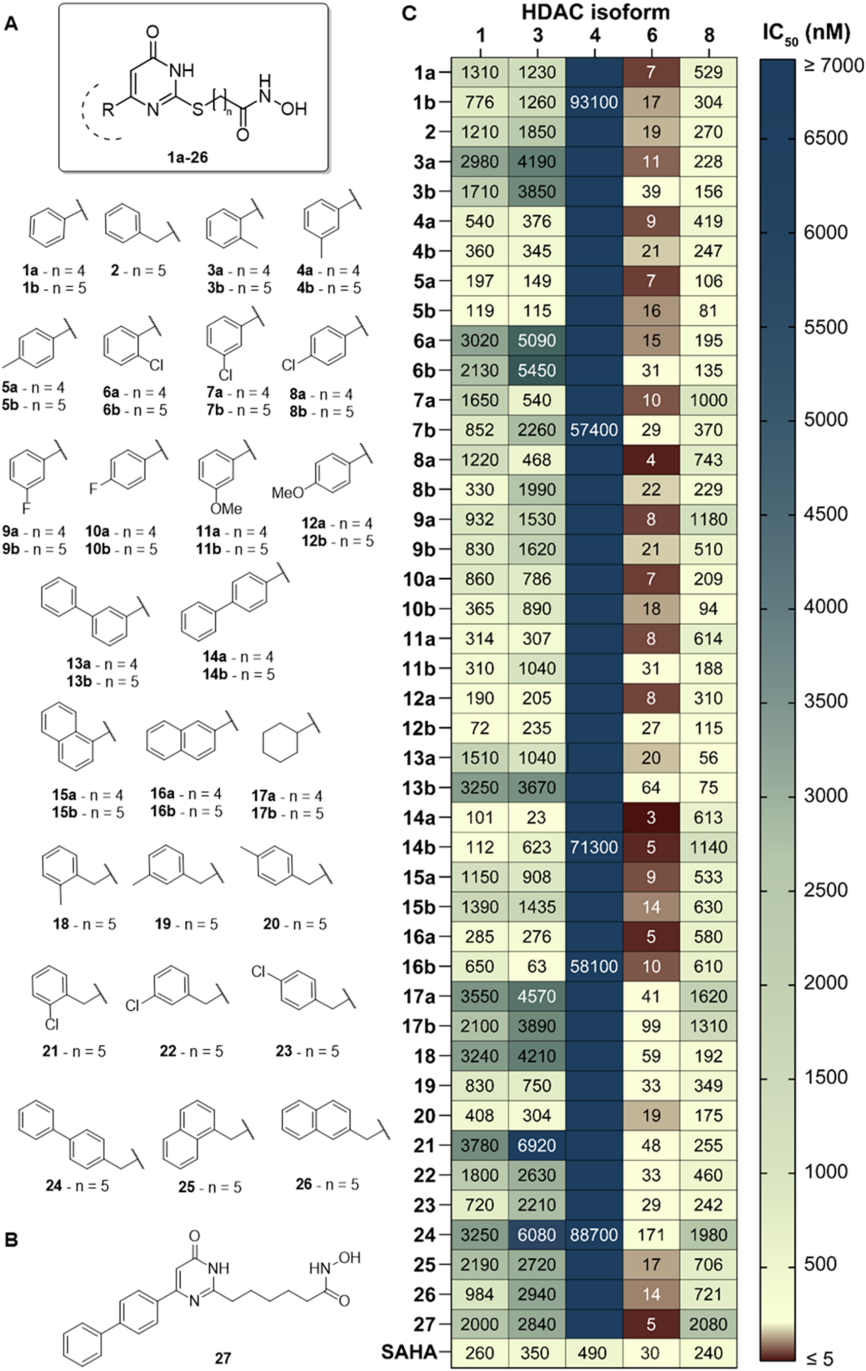
(A) Chemical structures of UBHAs **1a**–**26**. (B) Chemical structure of compound **27**. (C) Heatmap depicting the HDAC inhibition profiles of compounds **1a**–**27** and SAHA, expressed as IC_50_ values (nM) against human recombinant HDAC1, 3, 4, 6, 8. Values corresponding to an IC_50_ > 100 μM are not shown.

To further investigate potential aromatic surface interactions, we incorporated bulkier phenyl moieties in the *meta* and *para* positions, resulting in biphenyl derivatives **13a**, **13b**, **14a**, and **14b**. Similarly, compounds **15a**,**b** and **16a**,**b** feature 1- and 2-naphthyl rings, respectively, providing increased rigidity and aromatic surface area. In contrast, compounds **17a** and **17b** feature a cyclohexyl group as a nonaromatic cap, enabling us to assess the impact of purely aliphatic bulk on enzyme binding. For each of these UBHAs, we synthesized both four- and five-carbon linkers to probe the crucial role of linker length in accurately positioning the hydroxamic acid moiety within the HDAC active site (Figure 2A).

Compounds **18**–**26** are analogues of the lead molecule **2**, all bearing benzyl-based substituents at C6 and incorporating a five-carbon linker. Within this 6-benzyl series, we examined many of the same substituents explored in the first 6-phenyl series, including *ortho*, *meta*, and *para* methyl groups, *ortho*, *meta*, and *para* chloro groups, *meta* and *para* phenyl substituents, and both 1- and 2-naphthyl moieties (Figure 2A). Finally, compound **27** is an analogue of **14a** in which the sulfur atom is replaced with a methylene group (Figure 2B).

We initially evaluated the synthesized derivatives for their inhibitory activity against HDAC1, HDAC3, and HDAC8, which are representative of class I HDACs, as well as against the class IIA enzyme HDAC4 and the class IIB isoform HDAC6. Overall, the compounds developed here emerged as preferential inhibitors of HDAC6, displaying IC_50_ values in the nanomolar range, while inhibiting class I HDACs with potencies ranging from nanomolar to micromolar, and showing negligible activity toward HDAC4 (Figure 2B, Table S2).

Focusing first on the 6-phenyl derivatives, we observed that the length of the linker connecting the uracil core to the hydroxamic acid moiety consistently influenced inhibitory activity. Compounds bearing a four-carbon linker consistently exhibited stronger inhibition of HDAC3 and HDAC6 compared to their five-carbon counterparts. Conversely, the four-carbon linker proved detrimental for HDAC1 and, to a lesser extent, HDAC8 inhibition (Figure 2B, Table S2).

Moreover, *ortho*-substitution on the phenyl ring negatively impacted activity, while *meta*- and, even more so, *para*-substitution conferred better inhibition of HDAC1, HDAC3, HDAC6, and HDAC8. For this reason, we restricted the synthesis of fluoro, methoxy, and biphenyl derivatives (compounds **9a** through **14b**) to *meta* and *para* substitution patterns. The inhibitory activity of the 3′-biphenyl derivatives **13a** and **13b** against the tested HDAC isoforms showed a significant reduction (e.g., IC_50_ values vs HDAC6 of 20 and 64 nM, respectively). Differently, the 4′-biphenyl derivatives exhibited enhanced HDAC inhibitory activity, achieving HDAC6 inhibition in the low nanomolar range, with IC_50_ values 6 to 10 times lower compared to SAHA. Compound **14a** emerged as the most potent HDAC6 inhibitor of the series (IC_50_ = 2.7 nM), while **14b** ranked among the top five (IC_50_ = 4.9 nM). Importantly, compound **14a** also demonstrated notable HDAC3 inhibition (IC_50_ = 23 nM), resulting in an 8.5-fold selectivity for HDAC6 over HDAC3, whereas **14b** was over 120-fold selective for HDAC6 versus HDAC3 (Figure 2B, Table S2).

Condensation of two phenyl rings proved beneficial for HDAC inhibition, as evidenced by the naphthyl derivatives **15a**,**b** and **16a**,**b**, which were also low-nanomolar inhibitors of HDAC6. Consistent with trends observed among the phenyl derivatives, the 2-naphthyl compounds **16a** and **16b**, in which steric hindrance is located further from the uracil core, were slightly more potent than the 1-naphthyl analogues **15a** and **15b**. Finally, the significant reduction in activity observed with the 6-cyclohexyl derivatives **17a**,**b** suggests that a planar aromatic moiety is essential for maintaining high inhibitory potency.

When examining the 6-benzyl derivatives **18**–**26** alongside their five-carbon linker counterparts from the 6-phenyl series, it becomes evident that the benzylic substitution decreases compound activity. Nonetheless, the same trend persists concerning the relative position of the substituent, with *para* substitution being most favorable, followed by *meta*, and then *ortho* (Figure 2B, Table S2).

Motivated by the promising HDAC inhibitory activity observed for compound **14a**, we explored the role of the sulfur atom serving as a bridge between the uracil CU and the linker. Interestingly, derivative **27**, in which the sulfur atom of **14a** was replaced by an additional methylene group, exhibited a different activity profile compared to its analogue. Specifically, the inhibitory potency of **27** toward class I HDACs decreased substantially (IC_50_ values around 2 μM for HDAC1, 3, and 8), while it retained low-nanomolar inhibition of HDAC6 (IC_50_ = 4.9 nM).

Based on these findings, we set out to evaluate the most potent compounds possessing also an optimal calculated Caco-2 permeability^59^ (higher than 6 × 10^–6^ cm/s) against the full panel of HDAC isoforms (Table S2). To this end, we extended the profiling of compounds **8a**, **8b**, **14a**, **14b**, **16a**, **16b**, and **27** to include their inhibitory activity toward HDAC2, 5, 7, 9, 10, and 11 and compared the results with those of SAHA (Table 1). This evaluation revealed that all UBHAs displayed weak or negligible inhibition toward HDAC2, 5, 7, 9, and 11, whereas SAHA exhibited submicromolar potency against these isoforms. In contrast, UBHA derivatives showed midnanomolar IC_50_ values against HDAC10 and were consistently more potent than SAHA, thereby confirming the overall preference of this series for class IIB HDACs. Among the chloro-substituted derivatives, compound **8a** showed moderate inhibition of HDAC10 (IC_50_ = 85.1 nM), albeit still substantially weaker than its activity against HDAC6. Compound **8b** exhibited slightly improved potency against the newly tested isoforms, with IC_50_ values approximately 1.4- to 3-fold lower than those of **8a**.

**Table 1:**
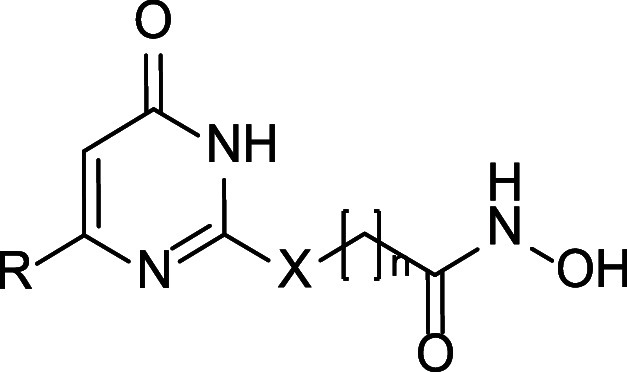

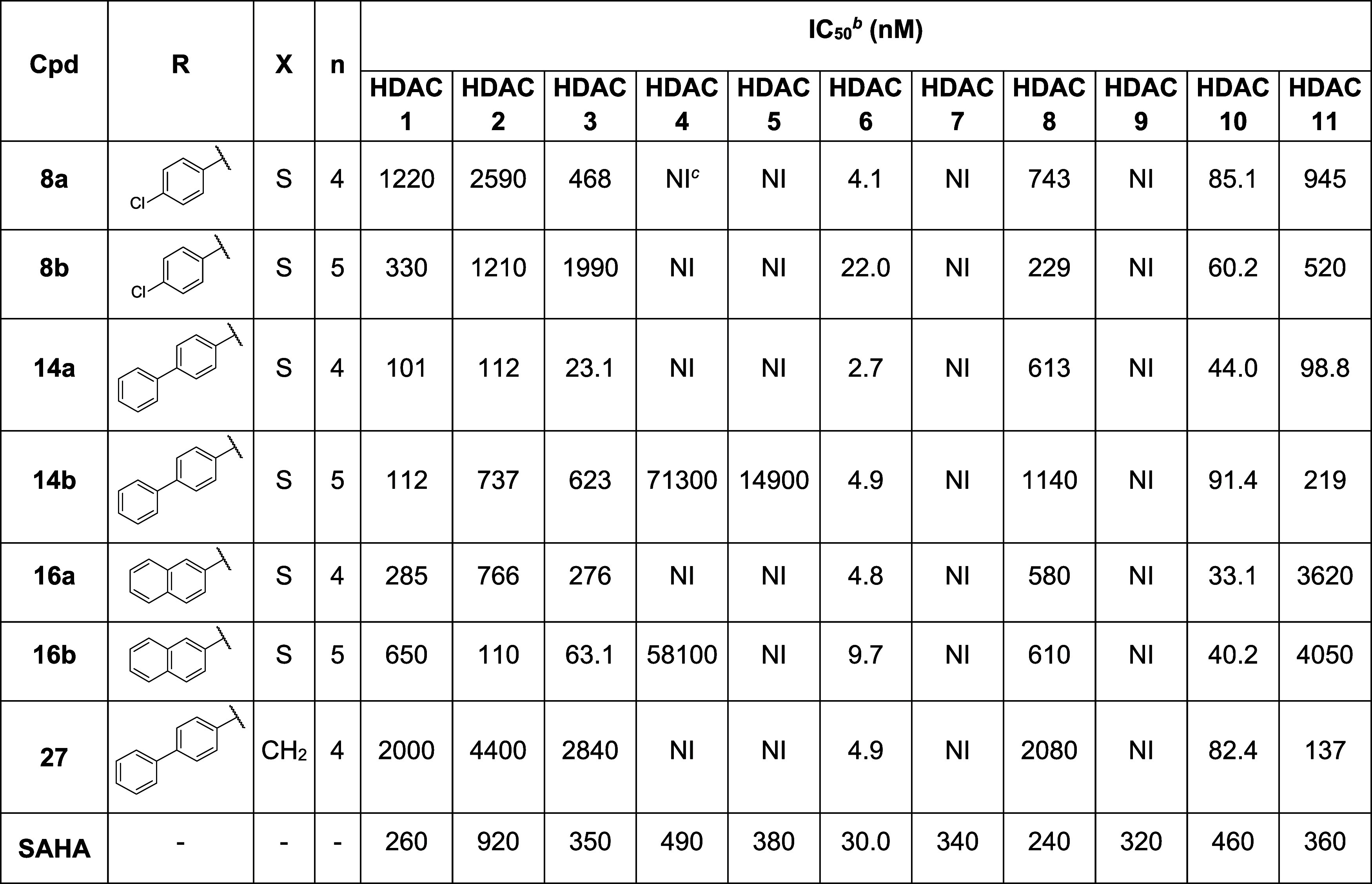
Inhibitory Activity of Compounds **8a**,**b**,**14a**,**b**,**16a**,**b**, **27** and SAHA against HDAC1-11^a^

a The compounds were tested against HDAC1, 3, 4, 6, 8 in a 10-dose IC_50_ mode with 3-fold serial dilution starting from 100 μM solutions.

b Inhibitory dose 50: dose required to inhibit the enzymatic activity by 50%.

c NI, no inhibition at 100 μM.

In the biphenyl series, compound **14a** showed overall higher activity across the newly tested HDAC isoforms, displaying midnanomolar inhibition of HDAC10 (IC_50_ = 44.0 nM) and moderate activity against HDAC11 (IC_50_ = 98.8 nM). Compound **14b** exhibited 7-fold weaker inhibition of HDAC2 compared to **14a**, and 2-fold weaker activity against HDAC10 (IC_50_ = 91.4 nM) and HDAC11 (IC_50_ = 219 nM). A similar trend was observed for compound **27**, which inhibited HDAC2 only in the micromolar range, but showed midnanomolar potency against HDAC10 (IC_50_ = 82.4 nM) and HDAC11 (IC_50_ = 137 nM). The naphthyl derivatives **16a** and **16b** exhibited modest inhibition of HDAC2, with IC_50_ values of 766 and 110 nM, respectively, while demonstrating good inhibition of HDAC10 (IC_50_ = 33.1 nM for **16a** and 40.2 nM for **16b**). Notably, their activity against HDAC11 was considerably weaker than that of other tested compounds, with IC_50_ values in the 3–4 μM range (Table 1).

In summary, our systematic exploration of UBHAs highlights HDAC6 as the primary target across the series, while HDAC4 appears to be the least responsive, and has enabled us to establish important structure–activity relationship (SAR). A four-carbon linker generally enhances potency toward HDAC3 and HDAC6, whereas longer linkers reduce activity against HDAC1 and HDAC8. Substituent positioning strongly influences activity, with *para*-substitution consistently outperforming *meta* and *ortho* configurations in both 6-phenyl and 6-benzyl derivatives. Incorporation of bulkier aromatic substituents proved detrimental when placed at the *meta*/3′ position, as observed for compounds **13a** and **13b**, but was highly beneficial in the *para*/4′ position, yielding the potent compounds **14a** and **14b**. The presence of a sulfur bridge significantly contributes to potency against class I HDACs, as evidenced by reduced activity upon its replacement with a methylene moiety in compound **27**, although its removal improves selectivity for HDAC6. Further testing against other HDAC isoforms confirmed the preference of these compounds for class IIB HDACs, particularly through the preferential inhibition of HDAC10 observed across all tested molecules.

Overall, this detailed SAR study, combined with the calculation of Caco-2 permeability for each compound (Table S2), has led us to select several promising candidates for the evaluation in cancer settings. Specifically, compounds **8a**, **14a**, **14b**, **16a**, and **27** have emerged as key candidates for further investigation in cellular assays.

### 2.3 Selected UBHAs Significantly Reduce Cell Viability in a Panel of Solid and Hematological Cancer Cell Lines

To assess their ability to impair cell viability in different cancer cell types, we selected the novel compounds **8a**, **14a**, **14b**, **16a**, and **27** and evaluated their cytotoxic activity (CC_50_ values, Table 2) after 48 h of treatment in a panel of 9 human cancer cell lines that include lung carcinoma (H1299), colorectal carcinoma (HT29), glioblastoma (ADF), fibroblastoma (HT1080), breast adenocarcinoma (MCF7), melanoma (M14), ovarian carcinoma (OVCA3), promyelocytic leukemia (HL60), and AML (U937).

**Table 2:**
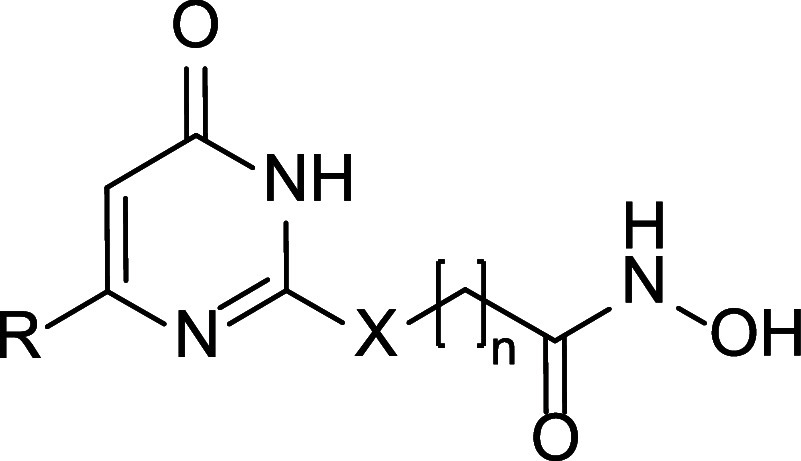

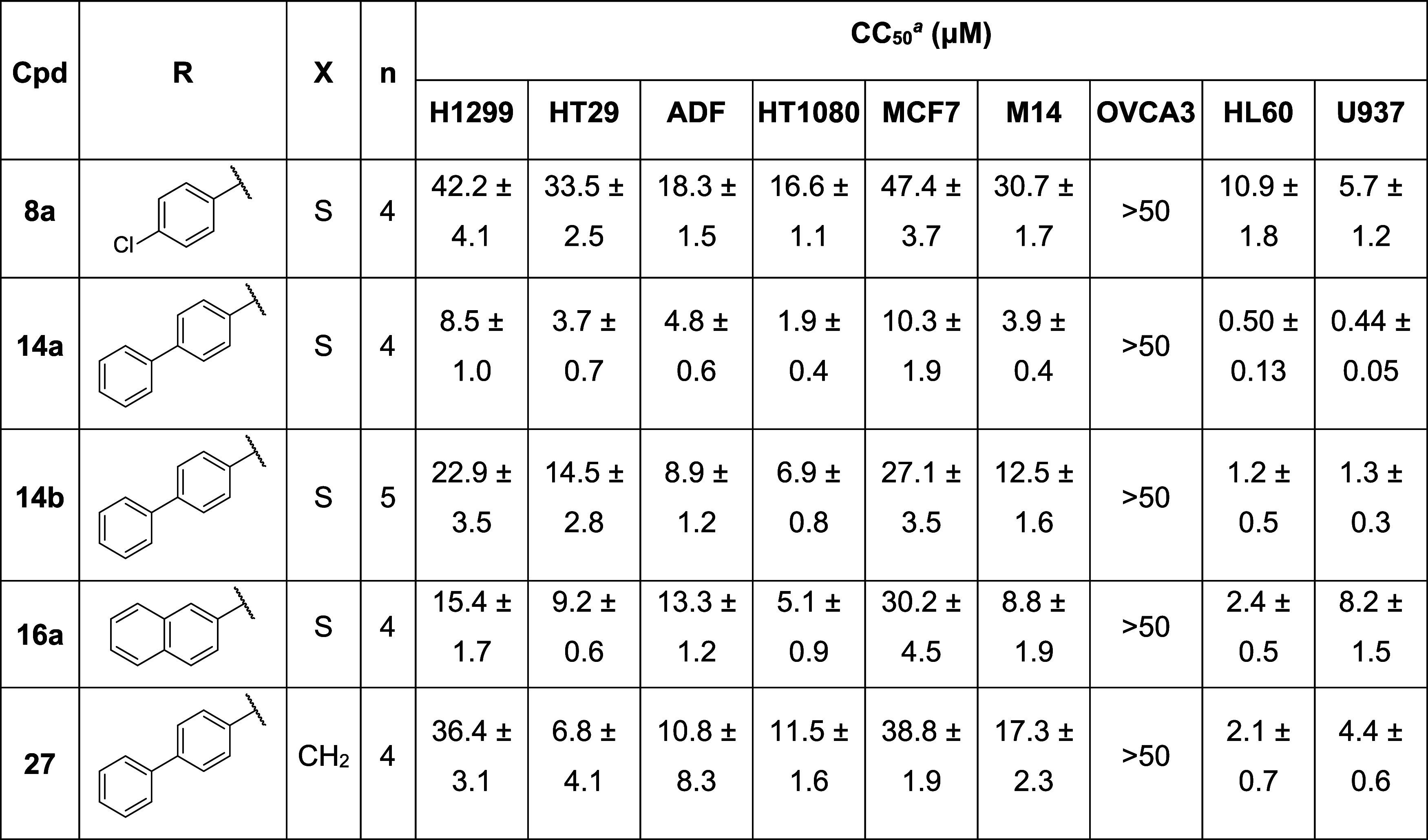
CC_50_ Values (μM) of **8a**, **14a**,**b**,**16a** and **27** after 48 h Treatment in a Panel of 9 Different Cancer Cell Lines

a CC_50_: Concentration required to reduce cell viability by 50%. Values are means ± standard deviation (SD).

Among the tested derivatives, the 6-(*para*-chloro)­phenyl derivative **8a** exhibited the mildest cytotoxic profile among the compounds tested, with CC_50_ values exceeding 30 μM in several cell lines, while still demonstrating good activity toward the hematological cancer cell lines HL60 and U937 with CC_50_ values of 10.9 and 5.7 μM, respectively. Conversely, the 6-biphenyl derivative **14a** emerged as the most potent compound overall, exhibiting submicromolar cytotoxic activity against the hematological cancer cells HL60 and U937 (CC_50_ values of 0.50 and 0.44 μM, respectively). Compound **14a** also showed low micromolar activity across several solid tumor lines, including HT1080 (CC_50_ = 1.9 μM), HT29 (CC_50_ = 3.7 μM), ADF (CC_50_ = 4.8 μM), and M14 (CC_50_ = 3.9 μM), while being completely inactive against the ovarian carcinoma cell line OVCA3. Compound **14b**, which also features a biphenyl substituent, but with an additional methylene spacer in the linker chain compared to **14a**, demonstrated significant reduction of cancer cell viability, particularly in leukemic cell lines, with CC_50_ values of 1.2 and 1.3 μM in HL60 and U937 cells, respectively. Moreover, **14b** maintained low micromolar activity in the solid cancer cell lines HT1080 (CC_50_ = 6.9 μM) and ADF (CC_50_ = 8.9 μM), albeit being generally less potent than its shorter counterpart **14a**. Compound **27**, a structural analogue of **14a** in which the sulfur linker has been replaced by a methylene group, retained acceptable activity against leukemic cell lines, displaying CC_50_ values of 2.1 μM in HL60 and 4.4 μM in U937 cells. Although generally less potent than **14a**, compound **27** confirms the contribution of the biphenyl scaffold to activity, particularly in hematologic malignancies. Finally, compound **16a**, bearing a 6-naphthyl moiety rather than a biphenyl system, moderately reduced the cell viability of most cell lines, with CC_50_ values ranging from 5.1 μM in HT1080 to 30.2 μM in MCF7, indicating that while the naphthyl group supports biological activity, it appears less effective than the biphenyl scaffold in driving high potency.

Taken together, these results highlight the biphenyl-containing derivatives, especially compound **14a**, as the most active compounds, particularly against leukemic cell lines (HL60 and U937). The comparison between **14a** and **14b** also underscores the importance of the linker nature for optimizing anticancer potency and reflects the differences observed in HDAC *in vitro* inhibition, whereby **14a** stood out as the most potent inhibitor, preferentially targeting HDAC6 and HDAC3, while **14b** was slightly less potent, albeit more HDAC6-selective.

We then assessed the cancer-selectivity of the most promising compound **14a** and its superior homologue **14b**. Importantly, we found that both HDACi **14a** and **14b** are cancer-selective, as they did not significantly affect the viability of noncancerous cells, including human fibroblasts (BJ), human endothelial cells (EA.hy926), human mammary epithelial cells (HME), and B lymphocyte AHH1 cells.

Before proceeding with further cellular evaluation, we also assessed the stability of **14a** and **14b** in PBS (pH 7.4) at 37 °C. After 48 and 72 h, we did not observe significant loss of either compound under these conditions, suggesting no appreciable degradation (Figures S6–S7). Moreover, both HDACi exhibited a high estimated Caco-2 permeability (Table S3 and Figure S8) while not compromising the integrity of the Caco-2 epithelial barrier (Figure S9), with experimental *P*_app_ values of 1.17·10^–5^ and 1.24·10^–5^ cm/s.

**Figure 3:**
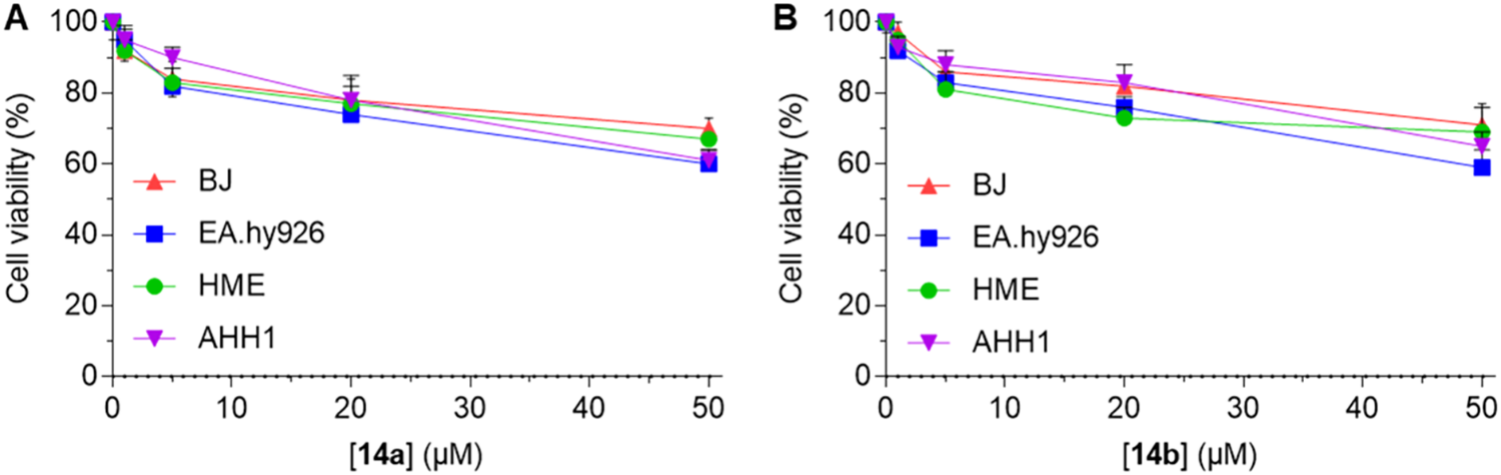
Effect of compounds **14a** (A) and **14b** (B) on the viability of human fibroblasts (BJ), endothelial cells (EA.hy926), mammary epithelial cells (HME), and B lymphocyte AHH1 cells after 48 h incubation at 1, 5, 20, and 50 μM.

### 2.4 Compounds **14a** and **14b** Promote Apoptosis in U937 Cells

Next, we selected the 6-biphenyl derivatives **14a** and **14b** to investigate their impact on cell cycle progression and apoptosis induction in AML U937 cells. To this end, we quantified the percentage of cells in the sub-G1 phase and the proportion of Annexin V-positive cells by flow cytometry. For these experiments, **14a** and **14b** were tested at concentrations of 0.125 and 0.25 μM after 24 and 48 h of treatment, with SAHA (1 μM) used as reference HDACi (Figure 4). Cell cycle distribution profiles of U937 cells revealed that SAHA induced a significant, time-dependent G0–G1 arrest, with only a minimal sub-G1 population (∼5%) relative to control. In contrast, both **14a** and **14b** triggered a marked, time- and dose-dependent accumulation of cells in the sub-G1 phase, indicative of apoptosis. At 24 h, treatment with these compounds led to at least 30% of cells in sub-G1, with compound **14a** at 0.25 μM reaching approximately 50%. This effect became even more pronounced after 48 h, with sub-G1 populations rising to 60–80% for higher concentrations of both compounds, whereas SAHA continued to show no significant impact under the same experimental conditions (Figure 4A,B).

**Figure 4:**
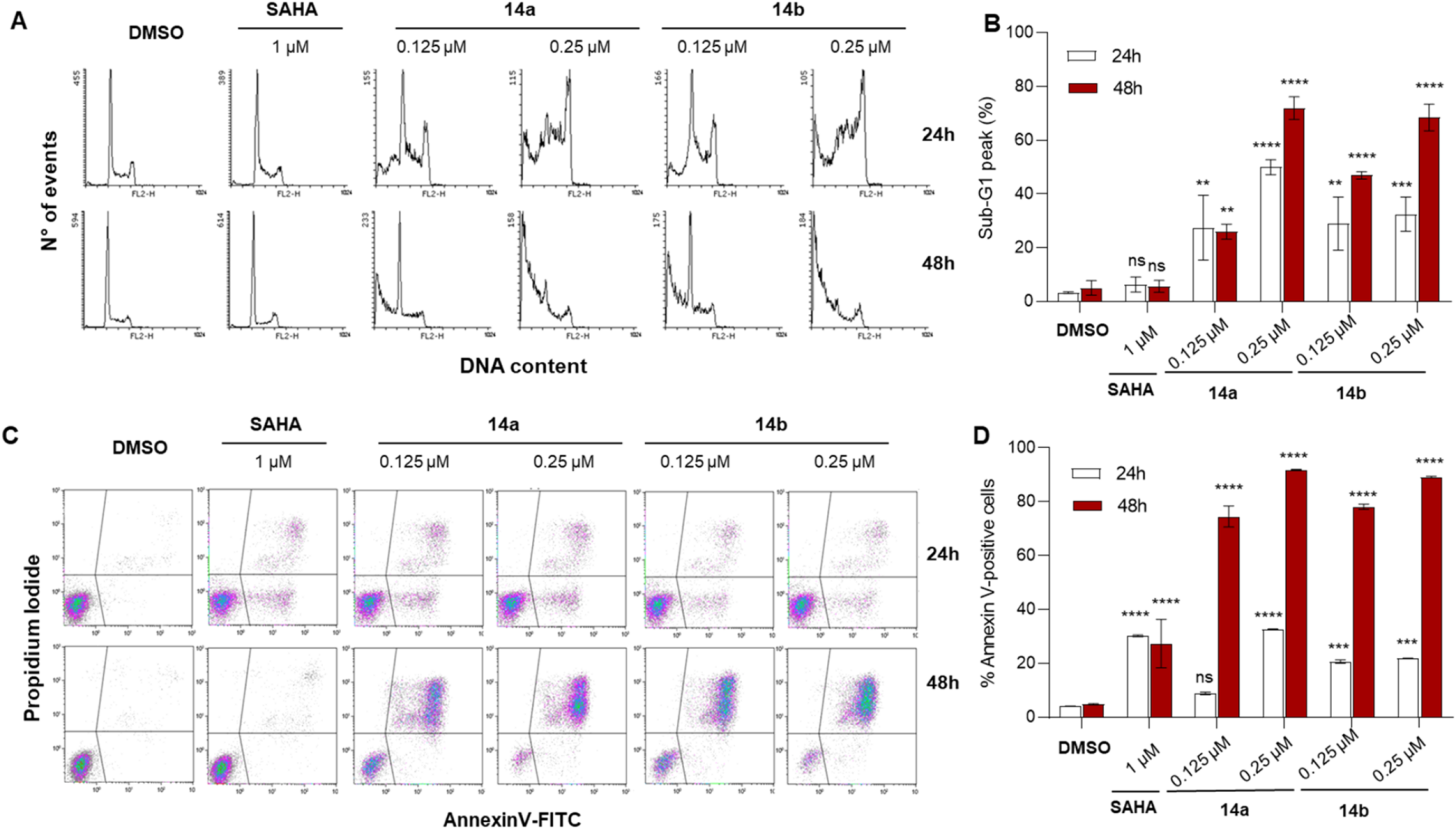
(A) Representative histograms of DNA content evaluated by flow cytometry in U937 cells treated with the indicated compounds for 24 and 48 h. (B) Quantification of the percentage of cells in the sub-G1 phase after treatment as indicated in (A). (C) Representative dot plots of apoptosis analysis by Annexin V-FITC/PI staining in U937 cells treated with the indicated compounds for 24 and 48 h. (D) Quantification of Annexin V-positive cells following treatments described in (C). Data are presented as mean ± SD; statistical analysis was performed using two-way ANOVA (ns, nonsignificant, ***p < 0.001; ****p < 0.0001). Control consists of 0.5% (v/v) DMSO-treated cells.

Having demonstrated that both **14a** and **14b** induce a sub-G1 peak, indicative of apoptosis in U937 cells, we next investigated their pro-apoptotic effects more directly through Annexin V-FITC/PI staining and flow cytometry analysis (Figure 4C,D). Untreated cells displayed minimal Annexin V positivity (∼5–10%). In contrast, treatment with **14a** and **14b** led to a marked, time- and concentration-dependent increase in Annexin V-positive cells. At 24 h, both compounds induced apoptosis in approximately 40–50% of the cell population at 0.125 μM, with levels rising to over 60% at 0.25 μM, similar or slightly exceeding the effects observed for SAHA, which reached around 35% Annexin V-positive cells at this time point. After 48 h, the pro-apoptotic effect became even more pronounced, with both compounds inducing apoptosis in more than 80% of cells at the higher concentration. Altogether, these findings confirm the strong pro-apoptotic activity of the two biphenyl-substituted UBHAs in U937 cells (Figure 4C,D).

Taken together, these results demonstrate that compounds **14a** and **14b** effectively induce apoptosis in U937 cells, as evidenced by both the significant accumulation of cells in the sub-G1 phase and the high percentages of Annexin V-positive cells. Their pro-apoptotic effects were markedly stronger than those observed with SAHA, highlighting the potent activity of these derivatives against leukemia cells.

### 2.5 Compounds **14a** and **14b** Inhibit HDAC Nuclear and Cytoplasmic Activity and Induce the Expression of Cell Cycle Regulators in U937 Cells

To assess the target engagement of the biphenyl derivatives **14a** and **14b**, we performed Western blot (WB) analyses in U937 cells after 48-h treatments with increasing compound concentrations (up to 0.25 μM), using SAHA (1 μM) as positive control. We initially analyzed the levels of acetylated histone H3 at lysine 9 (H3K9Ac) to evaluate the compounds’ ability to inhibit nuclear HDACs. Treatment with both **14a** and **14b** resulted in pronounced hyperacetylation of H3K9 across all tested concentrations, indicating effective nuclear HDAC inhibition comparable to or exceeding that of SAHA at 1 μM (Figure 5A).

**Figure 5:**
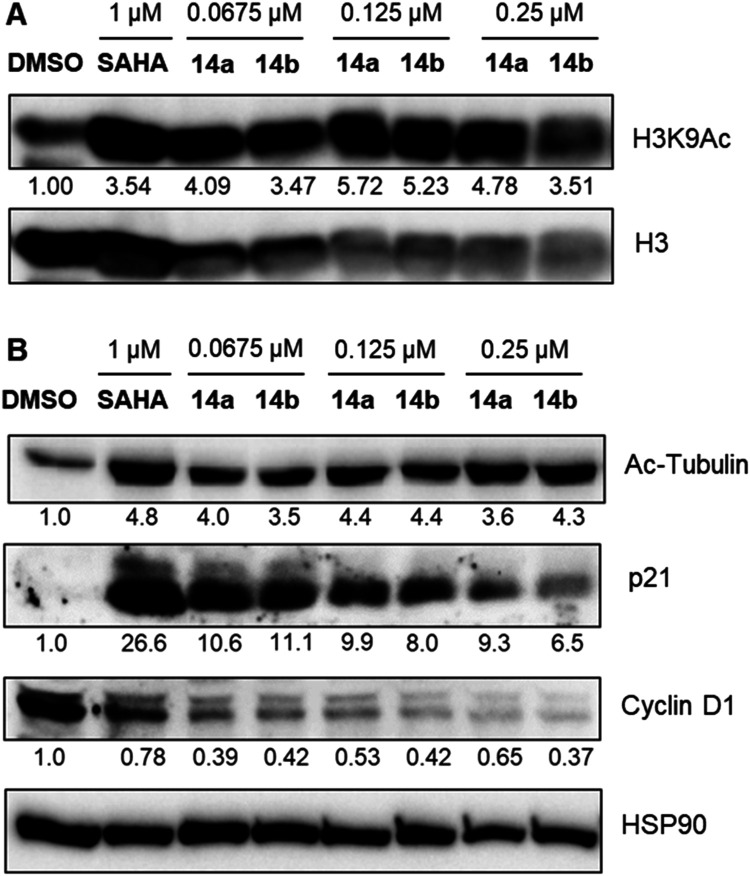
(A) Representative WB analysis of acetylated histone 3 at lysine 9 (H3K9Ac) in U937 cells treated with SAHA (1 μM) or increasing concentrations (0.0675, 0.125, and 0.25 μM) of compounds **14a** and **14b** for 48 h. Histone H3 was used as a loading control. Densitometric values are reported below each lane and represent fold-change relative to DMSO (set to 1) after normalization to total H3. (B) Representative WB analysis of acetylated α-tubulin (Ac-Tubulin), p21, and cyclin D1 in U937 cells treated with SAHA (1 μM) or increasing concentrations (0.0675, 0.125, and 0.25 μM) of compounds **14a** and **14b**. HSP90 was used as a loading control. Densitometric values are reported below each lane and represent fold-change relative to DMSO (set to 1) after normalization to HSP90. Control consists of 0.5% (v/v) DMSO-treated cells.

We also assessed acetylation levels of α-tubulin as a functional readout for HDAC6 inhibition. Notably, treatment with both compounds led to increased acetylated α-tubulin levels at all concentrations, reflecting potent inhibition of cytoplasmic HDAC6 activity (Figure 5B). To further characterize the biological implications of HDAC inhibition, we examined the expression levels of p21 and cyclin D1. Treatment with **14a** and **14b** led to a marked upregulation of the oncosuppressor p21, a cyclin-dependent kinase inhibitor implicated in cell cycle arrest, while levels of cyclin D1, a key regulator of G1/S phase progression, were substantially reduced (Figure 5B). These results are consistent with the induction of cell cycle arrest, as evidenced by p21 upregulation and cyclin D1 downregulation, and are in accordance with the previously observed increase in the sub-G1 cell population, indicative of apoptosis, and with the observed influence on cell viability of both compounds.

### 2.6 Compounds **14a** and **14b** Induce Pro-Apoptotic Gene and miRNA Changes in U937 Cells

To further explore the molecular mechanisms underlying the cytotoxic and pro-apoptotic effects of **14a** and **14b**, we performed gene expression analysis by qRT-PCR in U937 cells treated for 48 h with increasing concentrations of both compounds, using SAHA as a reference. We found that p21 mRNA expression was significantly increased in all treated samples (Figure 6, upper left panel), with both compounds inducing strong upregulation already at 0.0675 μM and maintaining elevated expression levels at higher doses. We also observed that both compounds caused a marked downregulation of cyclin D1 mRNA expression at all concentrations tested, showing a similar effect to SAHA (1 μM) (Figure 5, upper middle panel). These results are consistent with the upregulation of p21 mRNA and align with the observed effects on cell viability and cyclin D1 expression at the protein level (Figure 5). Furthermore, we found that treatment with **14a** and **14b**, but not SAHA, led to significant upregulation of the pro-apoptotic genes Bak and Bax (Figure 6, upper right and lower left panels) at all tested concentrations. Conversely, we observed that the antiapoptotic gene Bcl-2 was consistently downregulated in cells treated with either compound across all tested doses, while SAHA had no significant effect (Figure 6, lower right panel).

We then investigated the impact of **14a** and **14b** on the expression of key microRNAs (miRNAs) involved in apoptosis regulation in U937 cells. Regarding antiapoptotic miRNAs, we observed that treatment with **14a** and **14b** resulted in significant downregulation of miR-17-5p, miR-18-5p, miR-20a-5p, miR-21-5p, and miR-22-3p across all tested concentrations compared to control cells (Figure 7A). Notably, miR-18-5p and miR-22-3p were the most strongly suppressed, with expression levels reduced to less than 50% of control, and with effects stronger than those observed for SAHA.

We also found that **14a** and **14b** significantly promoted the expression of the pro-apoptotic miRNAs miR-122-5p, miR-769-5p, miR-181a-5p, and miR-181b-5p (Figure 7B). Expression of miR-122-5p was increased more than 5-fold in cells treated with **14a** at 0.25 μM, whereas SAHA had no significant effect. Similarly, both compounds induced marked upregulation of miR-769-5p and miR-181a-5p at all tested concentrations, with levels exceeding those observed in SAHA-treated cells. Interestingly, both compounds, but not SAHA, induced a significant increase in the expression levels of miR-181b-5p, another pro-apoptotic miRNA known to target Bcl-2 directly,^60^ further supporting their pro-apoptotic activity (Figure 7B).

These observations are mechanistically relevant, as downregulation of antiapoptotic miRNAs such as miR-21-5p may favor Bax activation and inhibition of Bcl-2 expression, consistent with previous reports in AML and pancreatic carcinoma cells.^61,62^ Moreover, the upregulation of pro-apoptotic miRNAs like miR-181b-5p and miR-769-5p has been linked to direct repression of Bcl-2 and enhanced apoptosis, as demonstrated in various cancer models, including gastric cancer treated with SAHA.^18^

Taken together, our gene and miRNA expression analyses demonstrate that **14a** and **14b** profoundly modulate transcriptional networks involved in apoptosis. The concurrent downregulation of antiapoptotic miRNAs and genes such as cyclin D1, along with the induction of pro-apoptotic miRNAs and genes like p21, Bax, and Bak, highlights a coordinated mechanism by which these compounds may promote apoptosis and inhibit cell proliferation in cancer cells.

### 2.7 Compounds **14a** and **14b** Exert (Sub)­micromolar Inhibition of Cell Viability in a Panel of Hematological Cancer Cells

Motivated by our previous findings, we selected the derivatives **14a** and **14b** to evaluate their efficacy in a panel of hematological cancer cell lines, including AML (OCI-AML3, IMS-M2, OCI-AML2, MV-411, Kasumi-1, U937), promyelocytic leukemia (NB4, HL60), and lymphoma (Karpas299) cell lines. We treated the cells for 48 h and determined CC_50_ values (Table 3). We found that both compounds exhibited low micromolar to submicromolar CC_50_ values across the tested cell lines, indicating potent cytotoxic activity. In general, we observed that **14a** was more potent than **14b**, showing lower CC_50_ values in all cell lines tested. Notably, **14a** showed the highest potency against promyelocytic leukemia cell lines, with CC_50_ values of 0.0837 μM in NB4 and 0.315 μM in HL60. Among the AML cell lines, we found that **14a** was particularly effective against MV-411 (0.098 μM) and Kasumi-1 (0.14 μM). We observed a similar trend for **14b**, which showed higher CC_50_ values ranging from 0.41 μM in NB4 to 2.86 μM in Karpas299 cells.

**Table 3:**
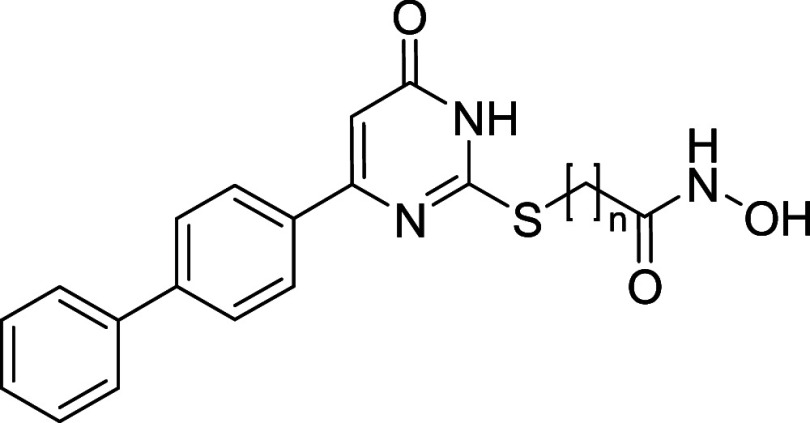
CC_50_ Values (μM) of **14a**,**b** after 48 h Treatment in a Panel of 9 Different Hematological Cancer Cell Lines

		CC_50_ ^a^ (μM)								
Cpd	n	OCI-AML3	IMS-M2	OCI-AML2	MV-411	Kasumi-1	NB4	HL60	Karpas299	U937
**14a**	4	0.89 ± 0.12	0.53 ± 0.06	0.95 ± 0.13	0.098 ± 0.006	0.14 ± 0.02	0.084 ± 0.004	0.32 ± 0.03	0.96 ± 0.10	0.39 ± 0.03
**14b**	5	1.8 ± 0.2	1.7 ± 0.2	2.1 ± 0.3	0.56 ± 0.07	0.69 ± 0.08	0.41 ± 0.05	1.0 ± 0.1	2.9 ± 0.4	1.2 ± 0.2

a CC_50_: Concentration required to reduce cell viability by 50%. Values are means ± standard deviation (SD).

### 2.8 Compound **14a** Targets Nuclear and Cytoplasmic Pathways to Suppress Prostate Cancer Cell Growth

We next investigated the effects of **14a** in prostate cancer cell lines. To capture different disease contexts, we selected PC-3 cells, which are androgen receptor (AR)-negative and represent androgen-independent prostate cancer, and 22Rv1 cells, which are AR-positive, but androgen-independent, modeling castration-resistant prostate cancer driven by active AR signaling. We characterized the impact of **14a** on cell proliferation kinetics by live-cell analysis and evaluated its antiproliferative activity in both cell lines. In PC-3 cells, treatment with increasing concentrations of **14a** led to a pronounced, dose-dependent inhibition of cell growth over 70 h compared to DMSO-treated controls (Figure 8A,B). Similar effects were observed in 22Rv1 cells, where **14a** significantly reduced proliferation in a concentration-dependent manner (Figure 8C,D). Brightfield imaging confirmed reduced cell density at 68 h in both cell lines treated with **14a** versus DMSO controls. We then determined the antiproliferative activity of compound **14a** after 48 h of treatment in PC-3 and 22Rv1 cells. Notably, we observed half-maximal growth inhibition (GI_50_) values of 0.73 and 1.25 μM, respectively, indicating potent antiproliferative effects in both models.

Importantly, in PC-3 cells, **14a** exhibited greater antiproliferative activity than SAHA when tested at the same concentrations (Figure 8E). To explore the underlying molecular mechanisms, we analyzed the expression of key markers associated with HDAC inhibition and apoptosis in the same cell line. Histone acetylation analysis revealed that **14a** markedly increased levels of acetylated H3K9 compared to DMSO controls, consistent with nuclear HDAC inhibition (Figure 8F). Furthermore, a 48-h treatment with **14a** (1 μM) led to a marked increase of α-tubulin acetylation, suggesting cellular inhibition of HDAC6 activity (Figure 8G). Finally, **14a** treatment (48 h) resulted in a slight increase of the cell cycle regulator and oncosuppressor p21, accompanied by downregulation of the antiapoptotic protein Bcl-2 (Figure 8H,I).

**Figure 6:**
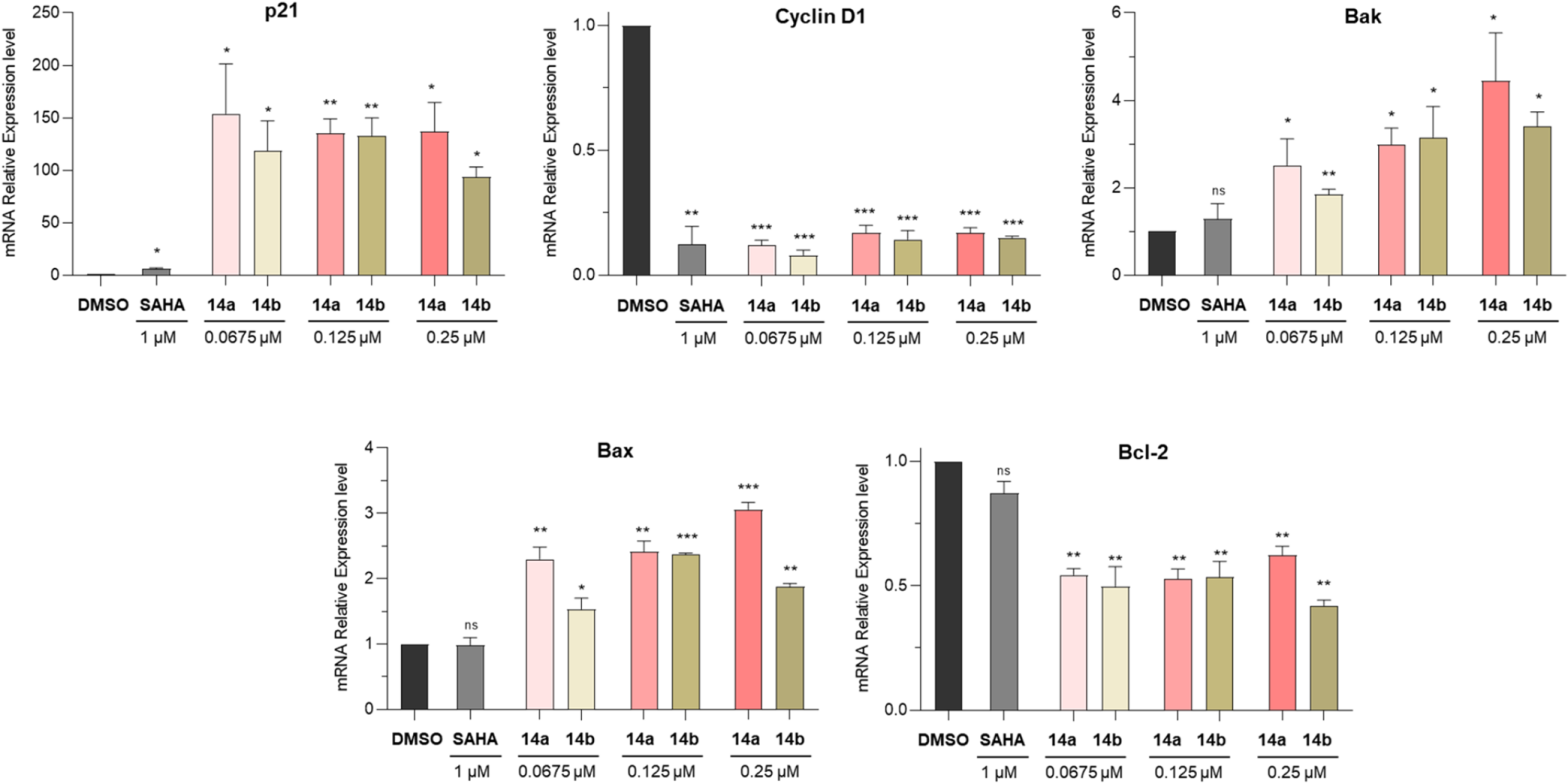
Expression of p21, cyclin D1, Bax, Bak, and Bcl-2 mRNA upon treatment of U937 cells with SAHA (1 μM) or compounds **14a** and **14b** at 0.0675, 0.125, and 0.25 μM for 48 h. Data are presented as mean ± SD; statistical analysis was performed using a Student’s *t* test (ns, nonsignificant, *p *<* 0.05; **p *<* 0.01; ***p < 0.001). Control consists of 0.5% (v/v) DMSO-treated cells.

Taken together, these results demonstrate that **14a** exerts potent antiproliferative activity in prostate cancer cell lines, associated with modulation of both cytoplasmic and nuclear HDAC targets and induction of pro-apoptotic molecular pathways. These findings highlight the potential of **14a** as a promising HDACi candidate for the treatment of prostate cancer.

## 3 Conclusions

HDACs remain central targets in the epigenetic landscape due to their pivotal roles in numerous cellular pathways whose aberrant regulation contributes to cancer and other diseases.^1–3,12,14^ Despite significant progress that has led to several HDACi reaching clinical use, challenges such as limited isoform selectivity, dose-limiting toxicities, and resistance still constrain their therapeutic application, especially in solid tumors.^14,26,29–31^ In addition, emerging insights into the regulatory effects of HDACs on nonhistone proteins and noncoding RNAs, including microRNAs, underscore the need for novel inhibitors with improved selectivity and multifaceted mechanisms of action.^15–18,20–24^

**Figure 7:**
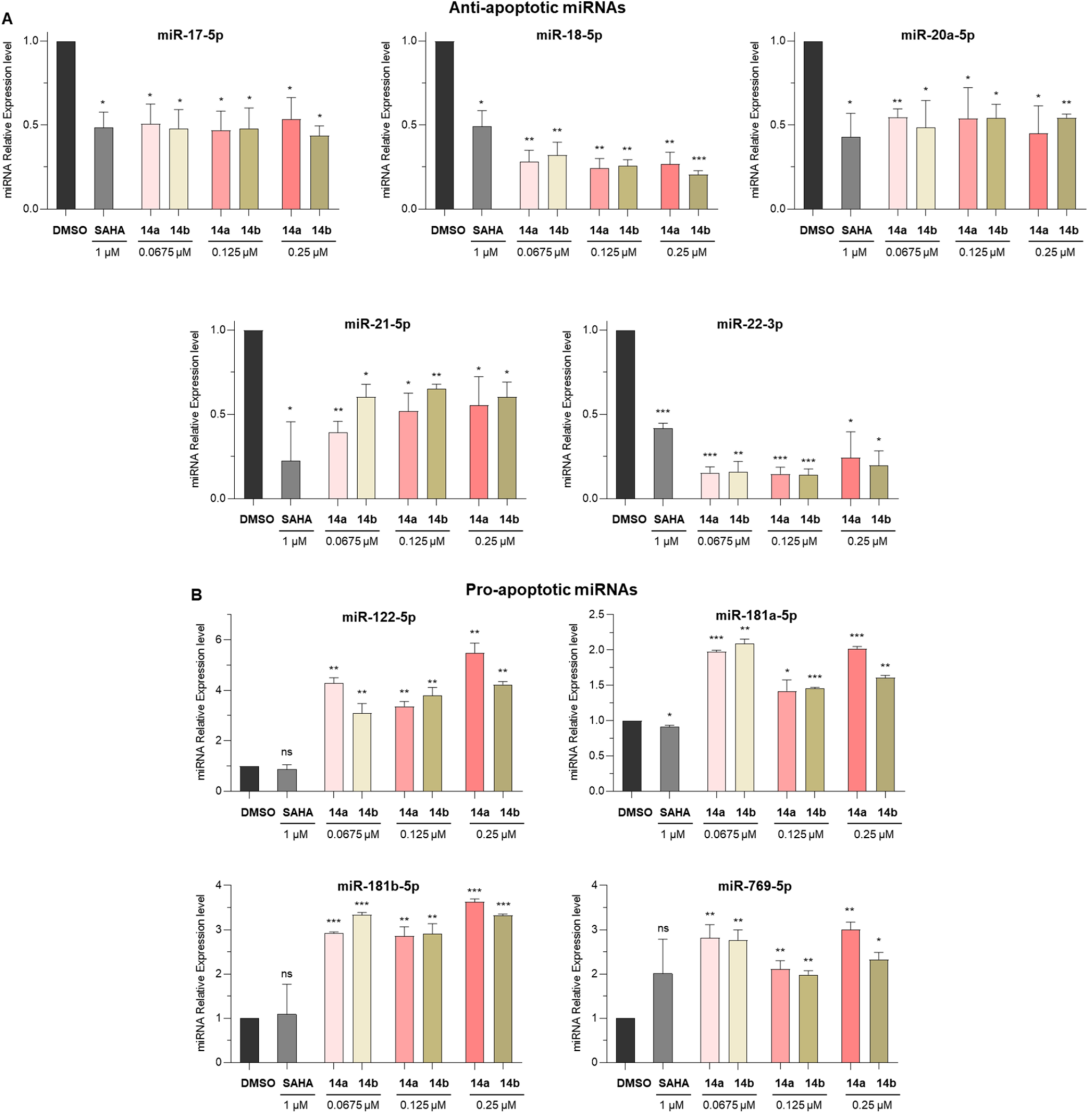
(A) Expression of antiapoptotic miRNAs miR-17-5p, miR-18-5p, miR-20a-5p, miR-21-5p, and miR-22-3p upon treatment of U937 cells with SAHA (1 μM) or compounds **14a** and **14b** at 0.0675, 0.125, and 0.25 μM for 48 h. (B) Expression of pro-apoptotic miRNAs miR-122-5p, miR-769-5p, miR-181a-5p, and miR-181b-5p upon treatment of U937 cells with SAHA (1 μM) or compounds **14a** and **14b** at 0.0675, 0.125, and 0.25 μM for 48 h. Data are presented as mean ± SD; statistical analysis was performed using a Student’s *t* test (ns, nonsignificant, *p *<* 0.05; **p *<* 0.01; ***p < 0.001). Control consists of 0.5% (v/v) DMSO-treated cells.

In this context, we have designed, synthesized, and evaluated a novel series of UBHAs as HDACi, introducing systematic variations in the aromatic cap group and linker region to optimize the potency and the isoform selectivity (Figure 2A). Our SAR studies revealed several key trends: a four-carbon linker generally enhanced inhibition of HDAC3 and HDAC6, while longer linkers reduced potency against HDAC1 and HDAC8. *Ortho* substitution on the phenyl cap was detrimental to activity, whereas *meta* and, especially, *para* substitution substantially improved inhibition across HDAC isoforms. Incorporation of bulkier aromatic moieties, such as biphenyl or naphthyl groups, at the C-6 position of the uracil central core led to significant gains in potency, particularly against HDAC6. Among the tested compounds, the 4′-biphenyl derivatives **14a** and **14b** emerged as the most promising compounds in the series. Indeed, **14a** exhibited an IC_50_ of 2.7 nM for HDAC6 and retained notable activity against HDAC3 (IC_50_ = 23 nM), achieving an 8.5-fold selectivity for HDAC6 over HDAC3, while **14b** displayed a slightly higher IC_50_ of 4.9 nM for HDAC6, but showed markedly greater selectivity (>120-fold) for HDAC6 over HDAC3 (Figure 2B). In contrast, benzylic substitution tended to decrease HDAC inhibition relative to phenyl analogues. Notably, replacing the sulfur bridge with a methylene group in compound **27** diminished activity against class I HDACs, though it preserved nanomolar potency against HDAC6. Collectively, these findings emphasize that subtle modifications at the cap group and linker regions strongly influence both potency and selectivity within the UBHA scaffold (Figure 2B).

Building upon the SAR analysis, we selected five compounds (**8a**, **14a**, **14b**, **16a**, and **27**) as the most promising candidates for cellular assays. These were chosen for their nanomolar inhibition of HDAC6, complementary activity against class I HDACs, and physicochemical properties compatible with good cellular uptake, based on calculated Caco-2 permeability (Table S2).

Among the tested compounds, **14a** and **14b** demonstrated robust anticancer activity across solid and hematological cancer cell lines. Notably, **14a** achieved submicromolar CC_50_ values in leukemic cell lines HL60 and U937 (0.50 and 0.44 μM, respectively), while maintaining low micromolar activity in solid tumors such as colorectal carcinoma, glioblastoma, and melanoma (Table 1). While **14b** was generally less potent, it still retained significant activity, highlighting the importance of linker architecture in modulating the biological effects. Importantly, both **14a** and **14b** exhibited cancer cell selectivity, with no significant cytotoxicity toward noncancerous cell types (Figure 3).

Mechanistic studies in U937 cells demonstrated that both compounds robustly induced apoptosis, as evidenced by substantial accumulation of cells in the sub-G1 phase and high levels of Annexin V-positive cells, exceeding the effects observed with SAHA (Figure 4). Consistent with their biochemical inhibition profiles, treatment with **14a** and **14b** led to significant hyperacetylation of nuclear H3K9 and cytoplasmic α-tubulin, confirming the inhibition of nuclear and cytoplasmic HDAC targets (Figure 5). Additionally, both compounds modulated key regulators of cell cycle and apoptosis, as demonstrated by increased expression of p21, Bax, and Bak mRNA, and decreased expression of cyclin D1 and Bcl-2 mRNA (Figure 6). For p21 and cyclin D1, these changes were also found at the protein level by WB analysis (Figure 5), overall supporting the compounds’ capacity to induce cell cycle arrest and apoptosis.

Furthermore, **14a** and **14b** profoundly altered the expression of microRNAs implicated in apoptosis regulation. Both compounds downregulated antiapoptotic miRNAs, such as miR-21-5p, miR-18-5p, and miR-22-3p, while significantly upregulating pro-apoptotic miRNAs including miR-181b-5p and miR-769-5p, underscoring a multifaceted mechanism for triggering apoptosis (Figure 7).

Of note, **14a** also demonstrated potent antiproliferative activity in prostate cancer cell models, achieving GI_50_ values in the sub- to low-micromolar range in both AR-negative PC-3 cells and AR-positive 22Rv1 cells (Figure 8A–E). Treatment with **14a** led to cellular effects consistent with inhibition of both nuclear and cytoplasmic HDACs and pro-apoptotic activity, including increased acetylation of H3K9 and α-tubulin and decreased Bcl-2 protein levels (Figure 8F–I).

**Figure 8:**
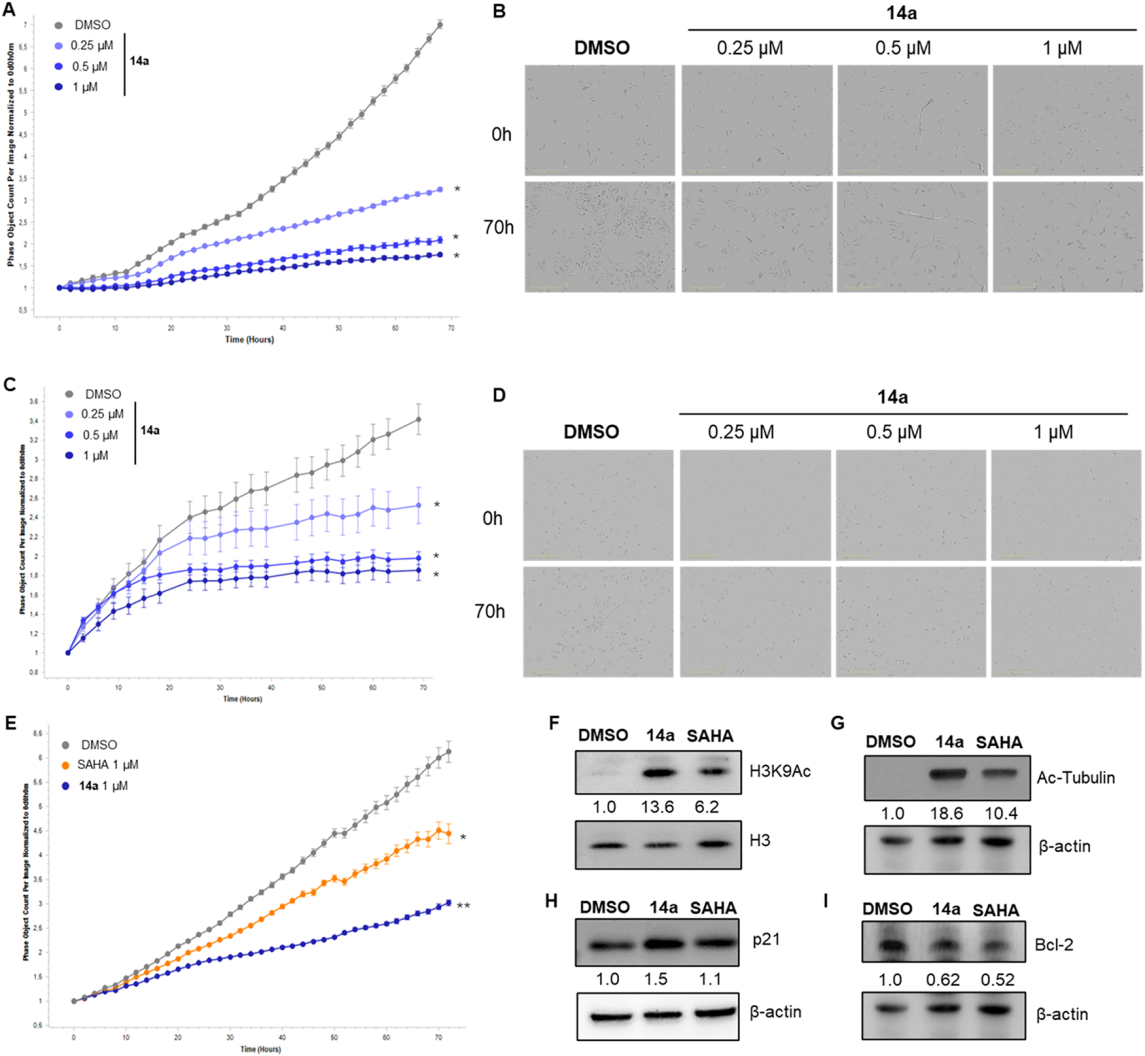
(A–D) Effect of compound **14a** on the proliferation of prostate cancer PC-3 (A, B) and 22Rv1 (C, D) cell lines. Cells were treated for 70 h with increasing concentrations of **14a** (0.25, 0.5, and 1 μM) or with DMSO (0.5%) as a control. Cell proliferation was monitored in real-time using the IncuCyte live-cell analysis system. Growth curves (A, C) are a single representative experiment out of three independent biological replicates; each data point shows mean ± SD of four replicates. Statistical analysis was performed using a Student’s *t* test (*p *<* 0.05). Control consists of 0.5% (v/v) DMSO-treated cells. Representative images (B, D) of cell confluence captured by the IncuCyte system; scale bar as indicated. (E) Effects of **14a** (1 μM) and SAHA (1 μM) on the proliferation of PC-3 prostate cancer cells. Cell proliferation was monitored using the IncuCyte live cell analysis system. Cell proliferation was calculated from raw data images; the data shown is a representative experiment of 3 biological replicates, and each time point represents mean ± SEM of 4 samples. Statistical analysis was performed using a Student’s *t* test (*p < 0.05; ** *p* < 0.01). Control consists of 0.5% (v/v) DMSO-treated cells. (F) Representative WB analysis of H3K9Ac in PC-3 cells treated with **14a** (1 μM) or SAHA (1 μM) for 48 h. Histone H3 was used as a loading control. (G-I) Representative WB analysis of acetylated α-tubulin (Ac-Tubulin, G), p21 (H), and Bcl-2 (I) in PC-3 cells treated with **14a** (1 μM) or SAHA (1 μM). β-actin was used as a loading control. Control consists of 0.5% (v/v) DMSO-treated cells. Number represents densitometric analysis of normalized protein level and expressed as fold change vs DMSO.

Taken together, our findings identify **14a** and **14b** as potent and selective HDAC inhibitors capable of engaging both nuclear and cytoplasmic targets and orchestrating extensive modulation of gene and miRNA regulatory networks involved in cell cycle control and apoptosis. These biphenyl UBHAs demonstrated robust cytotoxic and/or antiproliferative and pro-apoptotic effects across a range of hematological and solid tumor models, including notable efficacy in prostate cancer cell lines. Importantly, both compounds exhibited a favorable cancer-selectivity profile, showing minimal cytotoxicity toward noncancerous cells. Overall, our results highlight **14a** and **14b** as promising lead compounds for further development as anticancer therapeutics, with the potential to overcome some of the current limitations of HDAC-targeted therapy in oncology. In addition, their biochemical and biological features make them valuable chemical tools for probing the biological consequences of HDAC inhibition in both hematologic and solid tumor contexts.

## 4 Experimental Section

### 4.1 Chemistry

#### 4.1.1 General

Melting points were determined on a Buchi 530 melting point apparatus. ^1^H NMR and ^13^C NMR spectra were recorded at 400 and 100 MHz, respectively, with a Bruker AC400 spectrometer. Chemical shifts are reported in δ (ppm) units relative to the internal reference tetramethyl silane (Me_4_Si). Low-resolution mass spectra of final and intermediate compounds were recorded on a MSQ Plus Mass Spectrometer (Thermo Fisher), samples were injected by a Harvard pump using a flow rate of 100 μL/min, infused in the Electrospray system.

All compounds were routinely checked by TLC and ^1^H NMR; all final compounds were also checked by ^13^C NMR. TLC was performed on aluminum-backed silica gel plates (Merck DC, Alufolien Kieselgel 60 F254) with spots visualized by UV light. All solvents were reagent grade and, when necessary, were purified and dried by standard methods. Concentration of solutions after reactions and extractions involved the use of a rotary evaporator operating at reduced pressure of ca. 20 Torr. Organic solutions were dried over anhydrous sodium sulfate. Elemental analysis has been used to determine the purity of all final compounds that is >95%. Analytical results are within ± 0.40% of the theoretical values.

HPLC was also used to determine the purity of final compounds. The HPLC system consisted of a Dionex UltiMate 3000 UHPLC (Thermo Fisher) system equipped with an automatic injector, column heater and coupled with a Diode Array Detector DAD-3000 (Thermo Fisher). The analytical controls were performed on a Hypersil GOLD C18 Selectivity 5 μm (4.6 × 250 mm) HPLC Column (Thermo Fisher) in gradient elution. Eluents: (A) H_2_O + 0.1% TFA; (B) CH_3_CN + 0.1% TFA. The chromatographic run comprised 5 min at 10% solvent A, a subsequent 20 min linear gradient from 10 to 90% solvent B, and a final 5 min isocratic step at 90% solvent B. The flow rate was 1.0 mL/min, and the column was kept at a constant temperature of 30 °C. Samples were dissolved in solvent A at a concentration of 0.6 mg/mL and the injection volume was 1 μL.

All chemicals were purchased from Sigma-Aldrich s.r.l, Milan (Italy), or from TCI Europe N.V., Zwijndrecht (Belgium), and were of the highest purity.

#### 4.1.2 Synthesis

##### 4.1.2.1 General Procedure for the Synthesis of 6-Substituted 2-thioxo-2,3-dihydropyrimidin-4­(1*H*)-ones **28–53**

*Example: 2-thioxo-6-(o-tolyl)-2,3-dihydropyrimidin-4­(1H)-one (* **56** *)*. Thiourea (14 mmol, 1.4 equiv, 1.07 g) was added at room temperature to a clear solution of sodium ethoxide (obtained by dissolving 2 equiv of sodium metal in 23 mL of dried ethanol), followed by ethyl 3-oxo-3-(*o*-tolyl)­propanoate (**30**) (10 mmol, 1 equiv, 2.06 g). The reaction was stirred under reflux conditions for 16 h. Upon the conclusion of the reaction, the solvent was evaporated, and the resulting solid crude was dissolved in the minimum amount of water. The aqueous solution was then acidified with 2 N hydrochloric acid while cooling on an ice bath, obtaining a white solid in suspension that was filtered off, washed with water, and dried under vacuum to afford intermediate **56**, which was finally triturated with diethyl ether to provide a white solid that was used in the next step without further purification.

Mp: 278–282 °C. Yield: 49%; rec. solv.: ethanol. ^1^H NMR (400 MHz, DMSO-*d*_6_) δ 2.28 (s, 3H, Ph–C*H*_3_), 5.75 (s, 1H, C_5_-*H)*, 7.28–7.39 (m, 4H, benzene ring), 12.47 (d, 2H, 2 × -N*H*- uracil ring).

##### 4.1.2.2 General Procedure for the Synthesis of 6-Substituted Ethyl ((6-oxo-1,6-dihydropyrimidin-2-yl)­thio)­alkanoates **80a**–**105**

*Example: ethyl 5-((4-(naphthalen-1-yl)-6-oxo-1,6-dihydropyrimidin-2-yl)­thio)­pentanoate (* **94a** *)*. A mixture of 6-(naphthalen-1-yl)-2-thioxo-2,3-dihydropyrimidin-4­(1*H*)-one (**68**) (9.16 mmol, 1 equiv, 2.3 g), commercially available ethyl 6-bromohexanoate (10 mmol, 1.1 equiv, 1.8 mL), and anhydrous potassium carbonate (10 mmol, 1.1 equiv, 1.4 g) in 3 mL of anhydrous DMF was stirred at room temperature for 1 h. After quenching with cold water (100 mL), the resulting precipitate was filtered and washed over filter with water to furnish crude **68**. This was in turn purified by crystallization from an acetonitrile/methanol mixture to yield the pure intermediate **68**.

Mp: 120–122 °C. Yield: 59%; rec. solv.: acetonitrile/methanol. ^1^H NMR (400 MHz, CDCl_3_) δ 1.21 (t, 3H, −CH_2_C*H*_3_), 1.74–1.81 (m, 4H, -SCH_2_C*H*_2_C*H*_2_CH_2_CO−), 2.28 (t, 2H, −C*H*_2_CO−), 3.26 (t, 2H, −C*H*_2_S-), 4–08 (q, 2H, −C*H*_2_CH_3_), 6.58 (s, 1H, C_5_-*H*), 7.52–7.69 (m, 4H, 1-naphtyl ring), 7.91–7.97 (m, 2H, 1-naphtyl ring), 8.26–8.27 (m, 1H, 1-naphtyl ring), 12.78 (s, 1H, -N*H-* uracil ring).

##### 4.1.2.3 General Procedure for the Synthesis of 6-Substituted ((6-oxo-1,6-dihydropyrimidin-2-yl)­thio)­alkanoic acids **106a**–**131**

*Example: 6-((4-([1,1′-biphenyl]-4-ylmethyl)-6-oxo-1,6-dihydropyrimidin-2-yl)­thio)­hexanoic acid (* **129** *)*. A mixture of **103** (1.1 mmol, 1 equiv, 449 mg), 2 N potassium hydroxide (8.8 mmol, 8 equiv, 490 mg), and ethanol (5 mL) was stirred at room temperature for 18 h. The basic solution was then poured into water (50 mL) and extracted with ethyl acetate (2 × 20 mL). HCl (2 N) was added to the aqueous layer until pH 2 was reached. The resulting precipitate was then filtered off and recrystallized from an acetonitrile/methanol mixture to yield the intermediate **129** as a pure white pure solid.

Mp: 205–207 °C. Yield: 95%; rec. solv.: ethanol. ^1^H NMR (400 MHz, DMSO-*d*_6_) δ 1.30–1.33 (m, 2H, −C*H*_2_CH_2_CH_2_S−), 1.46–1.56 (m, 4H, −SCH_2_C*H*_2_CH_2_C*H*_2_CH_2_CO−), 2.16 (t, 2H, −C*H*_2_CO−), 3.05 (t, 2H, −C*H*_2_S-), 3.78 (s, 2H, Ph–C*H*_2_−), 5.99 (s, 1H, C_5_-*H*), 7.33–7.44 (m, 5H, 5 × −C*H*– 4′-biphenyl ring), 7.58–7.64 (m, 4H, 4 × −C*H*– 4′-biphenyl ring), 12.32 (br s, 2H, −COOH + −N*H*– uracil ring).

##### 4.1.2.4 General Procedure for the Synthesis of 6-Substituted ((6-oxo-1,6-dihydropyrimidin-2-yl)­thio)-*N*-hydroxyalkanamides **1a**–**26**

*Example: 6-((4-(4-fluorophenyl)-6-oxo-1*,*6-dihydropyrimidin-2-yl)­thio)-N-hydroxyhexanamide (MC1888-* **10b** *)*. Triethylamine (2.3 mmol, 2.6 equiv, 0.33 mL) and ethyl chloroformate (2.2 mmol, 2.4 equiv, 0.21 mL) were added in sequence to a 0 °C cooled solution of **115b** (0.9 mmol, 1 equiv, 315 mg) in anhydrous THF (5 mL), and the resulting mixture was stirred at room temperature for 10 min. The resulting white solid was quickly filtered off and the commercially available *O*-(2-methoxy-2-propyl)­hydroxylamine (5.4 mmol, 6 equiv, 0.4 mL) was added to the filtrate while cooling at 0 °C. The resulting mixture was then stirred at room temperature for 1 h, then was evaporated under reduced pressure and the residue was dissolved in methanol (5 mL). Amberlyst 15 ion-exchange resin (180 mg) was then added to the solution of the *O*-protected hydroxamate, and the resulting mixture was stirred at room temperature for 2 h. The reaction mixture was then filtered to remove the resin, and the filtrate was concentrated under vacuum to yield the crude compound **10b**, which was finally purified by crystallization from methanol.

Mp: 183–185 °C; yield: 58%; rec. solv.: methanol; ^1^H NMR (100 MHz, DMSO-*d*_6_) δ 1.38–1.40 (m, 2H, −C*H*_2_CH_2_CH_2_S−), 1.52–1.55 (m, 2H, −*CH*_2_CH_2_CO−), 1.68-1-72 (m, 2H, −*CH*_2_CH_2_S−), 1.95 (t, 2H, −C*H*_2_CO−), 3.21 (t, 2H, −C*H*_2_S−), 6.66 (s, 1H, C_5_-*H*), 7.33 (t, 2H, 2 × −C*H*– benzene ring), 8.12 (t, 1H, −C*H*– benzene ring), 8.67 (br s, 1H, −N*H*OH), 10.34 (s, 1H, −NHO*H*), 12.75 (s, 1H, -N*H-* uracil ring). ^13^C NMR (100 MHz, DMSO*-d*_6_) δ 25.2, 28.4, 29.1, 30.2, 32.6, 104.4, 116.1 (2C), 116.3 (2C), 129.6 (2C), 129.7 (2C), 132.98, 132.95, 152.3, 159.5, 162.9, 165.4, 169.5, 174.9. MS (ESI), *m*/*z*: 352 [M + H]^+^.

**MC1736** ***N*** **-hydroxy-5-((6-oxo-4-(*o*-tolyl)-1**,**6-dihydropyrimidin-2-yl)­thio)­pentanamide (3a)**. Mp: 186–188 °C; yield: 46%; rec. solv.:methanol; ^1^H NMR (400 MHz, DMSO-*d*_6_) δ 1.57–1.63 (m, 4H, -SCH_2_C*H*_2_C*H*_2_CH_2_CO−), 1.94 (t, 2H, −C*H*_2_CO−), 2.35 (s, 3H, Ph–C*H*_3_),3.12 (t, 2H, −C*H*_2_S-), 6.11 (s, 1H, C_5_-*H*), 7.24–7.41 (m, 4H, 4 × −C*H*-benzene ring), 8.76 (br s, 1H, -N*H*OH), 10.35 (s, 1H, -NHO*H*), 12.55 (s, 1H, -N*H-* uracil ring). ^13^C NMR (100 MHz, DMSO-*d*_6_) δ 25.5, 29.6, 33.7, 34.6, 37.0, 113.7, 131.2, 134.2, 134.4, 136.0, 140.8, 143.0, 167.2, 169.1, 174.0, 179.5. MS (ESI), *m*/*z*: 334 [M + H]^+^.

**MC1732** ***N*** **-hydroxy-6-((6-oxo-4-(*o*-tolyl)-1**,**6-dihydropyrimidin-2-yl)­thio)­hexanamide (3b)**. Mp: 202–204 °C; yield: 51%; rec. solv.: acetonitrile/methanol; ^1^H NMR (400 MHz, DMSO-*d*_6_) δ 1.30–1.32 (m, 2H, −C*H*_2_CH_2_CH_2_S-), 1.46–1.49 (m, 2H, -*CH*_2_CH_2_CO−), 1.62–1.64 (m, 2H, -*CH*_2_CH_2_S-),1.90 (t, 2H, −C*H*_2_CO−), 2.36 (s, 3H, Ph–C*H*_3_), 3.09 (t, 2H, −C*H*_2_S-), 6.16 (s, 1H, C_5_-*H*), 7.27–7.41 (m, 4H, 4 × −C*H*- benzene ring), 8.66 (br s, 1H, -N*H*OH), 10.33 (s, 1H, -NHO*H*), 12.63 (s, 1H, -N*H-* uracil ring). ^13^C NMR (100 MHz, DMSO-*d*_6_) δ 20.7, 25.1, 28.2, 29.1, 30.0, 32.6, 109.0, 126.4, 129.4, 129.6, 131.3, 136.0, 138.2, 162.3, 164.5, 169.4, 174.9. MS (ESI), *m*/*z*: 348 [M + H]^+^.

**MC1730** ***N*** **-hydroxy-5-((6-oxo-4-(*m*-tolyl)-1**,**6-dihydropyrimidin-2-yl)­thio)­pentanamide (4a)**. Mp: 185–187 °C; yield: 45%; rec. solv.: methanol; ^1^H NMR (400 MHz, DMSO-*d*_6_) δ 1.66–1.67 (m, 4H, -SCH_2_C*H*_2_C*H*_2_CH_2_CO−), 1.99 (t, 2H, −C*H*_2_CO−), 2.37 (s, 3H, Ph–C*H*_3_), 3.20 (t, 2H, −C*H*_2_S-), 6.64 (s, 1H, C_5_-*H*), 7.29–7.38 (m, 2H, 2 × −C*H*- benzene ring), 7.83–7.87 (m, 2H, 2 × −C*H*- benzene ring), 8.69 (br s, 1H, -N*H*OH), 10.35 (s, 1H, -NHO*H*), 12.60 (s, 1H, -N*H-* uracil ring). ^13^C NMR (100 MHz, DMSO-*d*_6_) δ 26.3, 29.7, 33.8, 34.8, 37.1, 109.7, 129.2, 132.6, 133.9, 136.5,141.2, 143.2, 165.6, 168.6, 174.0, 179.5. MS (ESI), *m*/*z*: 334 [M + H]^+^.

**MC1729** ***N*** **-hydroxy-6-((6-oxo-4-(*m*-tolyl)-1**,**6-dihydropyrimidin-2-yl)­thio)­hexanamide (4b)**. Mp: 194–196 °C; yield: 58%; rec. solv.: methanol; ^1^H NMR (400 MHz, DMSO-*d*_6_) δ 1.39–1.41 (m, 2H, −C*H*_2_CH_2_CH_2_S-), 1.52–1.56 (m, 2H, -*CH*_2_CH_2_CO−), 1.70–1.73 (m, 2H, -*CH*_2_CH_2_S-),1.95 (t, 2H, −C*H*_2_CO−), 2.36 (s, 3H, Ph–C*H*_3_), 3.20 (t, 2H, −C*H*_2_S-), 6.63 (s, 1H, C_5_-*H*), 7.29–7.37 (m, 2H, 2 × −C*H*- benzene ring), 7.83–7.86 (m, 2H, 2 × −C*H*- benzene ring), 8.68 (br s, 1H, -N*H*OH), 10.34 (s, 1H, -NHO*H*), 12.58 (s, 1H, -N*H-* uracil ring). ^13^C NMR (100 MHz, DMSO-*d*_6_) δ 21.5, 25.2, 28.5, 29.2, 30.2, 32.7, 104.9, 124.4, 127.8, 129.1,131.7, 136.5, 138.4, 160.6, 163.7, 169.4, 174.8. MS (ESI), *m*/*z*: 348 [M + H]^+^.

**MC1725** ***N*** **-hydroxy-5-((6-oxo-4-(*p*-tolyl)-1**,**6-dihydropyrimidin-2-yl)­thio)­pentanamide (5a)**. Mp: 176–178 °C; yield: 61%; rec. solv.: methanol; ^1^H NMR (400 MHz, DMSO-*d*_6_) δ 1.65–1.67 (m, 4H, -SCH_2_C*H*_2_C*H*_2_CH_2_CO−), 1.99 (t, 2H, −C*H*_2_CO−), 2.35 (s, 3H, Ph–C*H*_3_),3.21 (t, 2H, −C*H*_2_S-), 6.61 (s, 1H, C_5_-*H*), 7.28 (d, 2H, 2 × −C*H*- benzene ring), 7.94 (d, 2H, 2 × −C*H*- benzene ring), 8.70 (br s, 1H, -N*H*OH), 10.36 (s, 1H, -NHO*H*), 12.66 (s, 1H, -N*H-* uracil ring). ^13^C NMR (100 MHz, DMSO-*d*_6_) δ 21.4, 24.9, 29.0, 30.0, 32.3, 104.9, 127.2 (2C), 129.9 (2C), 133.7, 141.0,163.5, 164.5, 169.3, 174.8. MS (ESI), *m*/*z*: 334 [M + H]^+^.

**MC1726** ***N*** **-hydroxy-6-((6-oxo-4-(*p*-tolyl)-1**,**6-dihydropyrimidin-2-yl)­thio)­hexanamide (5b)**. Mp: 200–202 °C; yield: 54%; rec. solv.: methanol; ^1^H NMR (400 MHz, DMSO-*d*_6_) δ 1.36–1.39 (m, 2H, −C*H*_2_CH_2_CH_2_S-), 1.51–1.55 (m, 2H, -*CH*_2_CH_2_CO−), 1.70–1.72 (m, 2H, -*CH*_2_CH_2_S-),1.95 (t, 2H, −C*H*_2_CO−), 2.35 (s, 3H, Ph–C*H*_3_), 3.20 (t, 2H, −C*H*_2_S-), 6.61 (s, 1H, C_5_-*H*), 7.29 (d, 2H, 2 × −C*H*- benzene ring), 7.93 (d, 2H, 2 × −C*H*- benzene ring), 8.67 (br s, 1H, -N*H*OH), 10.35 (s, 1H, -NHO*H*), 12.69 (s, 1H, -N*H-* uracil ring). ^13^C NMR (100 MHz, DMSO-*d*_6_) δ 21.4, 25.2, 28.4, 29.1, 30.2, 32.7, 105.3, 127.2 (2C), 129.8 (2C), 133.7, 141.0, 169.4, 174.9. MS (ESI), *m*/*z*: 348 [M + H]^+^.

**MC1723 5-((4-(2-chlorophenyl)-6-oxo-1**,**6-dihydropyrimidin-2-yl)­thio)-** ***N*** **-hydroxypentanamide (6a)**. Mp: 204–206 °C; yield: 52%; rec. solv.: methanol; ^1^H NMR (400 MHz, DMSO-*d*_6_) δ 1.56–1.63 (m, 4H, -SCH_2_C*H*_2_C*H*_2_CH_2_CO−), 1.94 (t, 2H, −C*H*_2_CO−), 3.11 (t, 2H, −C*H*_2_S-), 6.32 (s, 1H, C_5_-*H*), 7.45–7.61 (m, 4H, 4 × −C*H*- benzene ring), 8.72 (br s, 1H, -N*H*OH), 10.34 (s, 1H, -NHO*H*), 12.83 (s, 1H, -N*H-* uracil ring). ^13^C NMR (100 MHz, DMSO-*d*_6_) δ 24.8, 28.9, 30.0, 32.2, 110.7, 127.9, 130.7, 131.4, 131.4, 131.5,137.1, 161.2, 162.7, 169.3, 175.9. MS (ESI), *m*/*z*: 354 [M + H]^+^.

**MC1724 6-((4-(2-chlorophenyl)-6-oxo-1**,**6-dihydropyrimidin-2-yl)­thio)-** ***N*** **-hydroxyhexanamide (6b)**. Mp: 188–192 °C; yield: 54%; rec. solv.: methanol; ^1^H NMR (400 MHz, DMSO-*d*_6_) δ ^1^H NMR (100 MHz, DMSO-*d*_6_): δ 1.31–1.32 (m, 2H, −C*H*_2_CH_2_CH_2_S-), 1.44–1.50 (m, 2H, -*CH*_2_CH_2_CO−), 1.63–1.66 (m, 2H, -*CH*_2_CH_2_S-),1.91 (t, 2H, −C*H*_2_CO−), 3.10 (t, 2H, −C*H*_2_S-), 6.31 (s, 1H, C_5_-*H*), 7.45–7.60 (m, 4H, 4 × −C*H*- benzene ring), 8.66 (br s, 1H, -N*H*OH), 10.32 (s, 1H, -NHO*H*), 12.78 (s, 1H, -N*H-* uracil ring). ^13^C NMR (100 MHz, DMSO-*d*_6_) δ 25.1, 28.2, 29.0, 30.1, 32.6, 106.4, 127.9, 130.7, 131.3, 131.4,131.5, 137.1, 160.8, 168.3, 169.4, 172.4. MS (ESI), *m*/*z*: 368 [M + H]^+^.

**MC1716 5-((4-(3-chlorophenyl)-6-oxo-1**,**6-dihydropyrimidin-2-yl)­thio)-** ***N*** **-hydroxypentanamide (7a)**. Mp: 196–198 °C; yield: 61%; rec. solv.: methanol; ^1^H NMR (400 MHz, DMSO-*d*_6_): δ 1.61–1.67 (m, 4H, -SCH_2_C*H*_2_C*H*_2_CH_2_CO−), 1.97 (t, 2H, −C*H*_2_CO−), 3.19 (t, 2H, −C*H*_2_S-), 6.73 (s, 1H, C_5_-*H*), 7.49–7.52 (m, 2H, 2 × −C*H*- benzene ring), 7.99–8.06 (m, 2H, 2 × −C*H*- benzene ring), 8.65 (br s, 1H, -N*H*OH), 10.32 (s, 1H, -NHO*H*), 12.82 (s, 1H, -N*H-* uracil ring). ^13^C NMR (100 MHz, DMSO-*d*_6_) δ 25.3, 29.3, 30.6, 32.7, 107.4, 127.4, 128.5, 132.3, 132.7, 135.7,140.3, 160.5, 164.9, 171.2, 176.7. MS (ESI), *m*/*z*: 354 [M + H]^+^.

**MC1717 6-((4-(3-chlorophenyl)-6-oxo-1**,**6-dihydropyrimidin-2-yl)­thio)-** *N* **-hydroxyhexanamide (7b)**. Mp: 188–192 °C; yield: 67%; rec. solv.: methanol; ^1^H NMR (400 MHz, DMSO-*d*_6_) δ ^1^H NMR (100 MHz, DMSO-*d*_6_) δ1.38–1.40 (m, 2H, −C*H*_2_CH_2_CH_2_S-), 1.52–54 (m, 2H, -*CH*_2_CH_2_CO−), 1.69–1.72 (m, 2H, -*CH*_2_CH_2_S-), 1.94 (t, 2H, −C*H*_2_CO−), 3.19 (t, 2H, −C*H*_2_S-), 6.75 (s, 1H, C_5_-*H*), 7.49–7.53 (m, 2H, 2 × −C*H*- benzene ring), 8.00–8.08 (m, 2H, 2 × −C*H*- benzene ring), 8.66 (br s, 1H, -N*H*OH), 10.33 (s, 1H, -NHO*H*), 12.76 (s, 1H, -N*H-* uracil ring). ^13^C NMR (100 MHz, DMSO-*d*_6_) δ 25.2, 28.4, 29.1, 30.3, 32.6, 106.1, 125.9, 127.0, 130.8, 131.1,134.2, 138.7, 158.6, 162.6, 169.4, 176.2. MS (ESI), *m*/*z*: 368 [M + H]^+^.

**MC1714 5-((4-(4-chlorophenyl)-6-oxo-1**,**6-dihydropyrimidin-2-yl)­thio)-** ***N*** **-hydroxypentanamide (8a)**. Mp: 198–200 °C; yield: 52%; rec. solv.: methanol; ^1^H NMR (400 MHz, DMSO-*d*_6_) δ 1.61–1.67 (m, 4H, -SCH_2_C*H*_2_C*H*_2_CH_2_CO−), 1.96 (t, 2H, −C*H*_2_CO−), 3.19 (t, 2H, −C*H*_2_S-), 6.67 (s, 1H, C_5_-*H*), 7.52 (d, 2H, 2 × −C*H*- benzene ring), 8.05 (d, 2H, 2 × −C*H*- benzene ring), 8.65 (br s, 1H, -N*H*OH), 10.32 (s, 1H, -NHO*H*), 12.72 (s, 1H, -N*H-* uracil ring). ^13^C NMR (100 MHz, DMSO-*d*_6_) δ 24.9, 28.9, 30.1, 32.3, 105.5, 129.1 (2C), 129.3 (2C), 135.4, 135.9, 159.0,163.1, 169.3, 174.8. MS (ESI), *m*/*z*: 354 [M + H]^+^.

**MC1715 6-((4-(4-chlorophenyl)-6-oxo-1**,**6-dihydropyrimidin-2-yl)­thio)-** ***N*** **-hydroxyhexanamide (8b)**. Mp: 183–186 °C; yield: 49%; rec. solv.: methanol; ^1^H NMR (400 MHz, DMSO-*d*_6_) δ 1.45–1.46 (m, 2H, −C*H*_2_CH_2_CH_2_S-), 1.52–1.56 (m, 2H, -*CH*_2_CH_2_CO−), 1.74-1-79 (m, 2H, -*CH*_2_CH_2_S-), 2.02 (t, 2H, −C*H*_2_CO−), 3.26 (t, 2H, −C*H*_2_S-), 6.77 (s, 1H, C_5_-*H*), 7.62 (d, 2H, 2 × −C*H*- benzene ring), 8.18 (d, 2H, 2 × −C*H*- benzene ring), 8.78 (br s, 1H, -N*H*OH), 10.42 (s, 1H, -NHO*H*), 12.78 (s, 1H, -N*H-* uracil ring). ^13^C NMR (100 MHz, DMSO-*d*_6_) δ 25.2, 28.4, 29.1, 30.2, 32.6, 105.0, 129.0 (2C), 129.3 (2C), 135.3, 135.9, 159.2, 162.9, 169.5, 174.9. MS (ESI), *m*/*z*: 368 [M + H]^+^.

**MC1910 5-((4-(3-fluorophenyl)-6-oxo-1**,**6-dihydropyrimidin-2-yl)­thio)-** ***N*** **-hydroxypentanamide (9a)**. Mp: 201–203 °C; yield: 50%; rec. solv.: ethanol; ^1^H NMR (400 MHz, DMSO-*d*_6_) δ 1.64–1.77 (m, 4H, -SCH_2_C*H*_2_C*H*_2_CH_2_CO−), 1.98 (t, 2H, −C*H*_2_CO−), 3.23 (t, 2H, −C*H*_2_S-), 6.74 (s, 1H, C_5_-*H*), 7.31–7.35 (m, 1H, −C*H*- benzene ring), 7.51–7.56 (m, 1H, −C*H*- benzene ring), 7.88 (dd, 2H, 2 × −C*H*- benzene ring), 8.66 (br s, 1H, -N*H*OH), 10.33 (s, 1H, -NHO*H*), 12.80 (s, 1H, -N*H-* uracil ring). ^13^C NMR (100 MHz, DMSO-*d*_6_) δ 24.9, 28.8, 30.1, 32.3, 105.3, 113.8, 114.0, 117.7, 117.9, 123.4,123.4, 131.2, 131.3, 139.0, 139.1, 159.0, 159.0, 161.7, 164.1, 168.1, 169.3, 174.7. MS (ESI), *m*/*z*: 338 [M + H]^+^.

**MC1911 6-((4-(3-fluorophenyl)-6-oxo-1**,**6-dihydropyrimidin-2-yl)­thio)-** ***N*** **-hydroxyhexanamide (9b)**. Mp: 173–175 °C; yield: 47%; rec. solv.: methanol; ^1^H NMR (400 MHz, DMSO-*d*_6_) δ 1.39–1.41 (m, 2H, −C*H*_2_CH_2_CH_2_S-), 1.52–1.54 (m, 2H, -*CH*_2_CH_2_CO−), 1.70-1-74 (m, 2H, -*CH*_2_CH_2_S-), 1.95 (t, 2H, −C*H*_2_CO−), 3.22 (t, 2H, −C*H*_2_S-), 6.74 (s, 1H, C_5_-*H*), 7.31–7.35 (m, 1H, −C*H*- benzene ring), 7.52–7.57 (m, 1H, −C*H*- benzene ring), 7.88 (dd, 2H, 2 × −C*H*- benzene ring), 8.63 (br s, 1H, -N*H*OH), 10.32 (s, 1H, -NHO*H*), 12.70 (s, 1H, -N*H-* uracil ring). ^13^C NMR (100 MHz, DMSO*-d*_6_) δ 25.2, 28.4, 29.0, 30.3, 32.6, 105.9, 113.8, 114.0, 117.7, 117.9,123.3, 123.4, 131.2, 131.3, 139.0, 139.1, 158.8, 161.7, 163.2, 164.1, 169.4, 174.8. MS (ESI), *m*/*z*: 352 [M + H]^+^.

**MC1887 5-((4-(4-fluorophenyl)-6-oxo-1**,**6-dihydropyrimidin-2-yl)­thio)-** ***N*** **-hydroxypentanamide (10a)**. Mp: 178–180 °C; yield: 61%; rec. solv.: methanol; ^1^H NMR (400 MHz, DMSO-*d*_6_) δ 1.63–1.69 (m, 4H, -SCH_2_C*H*_2_C*H*_2_CH_2_CO−), 1.99 (t, 2H, −C*H*_2_CO−), 3.22 (t, 2H, −C*H*_2_S-), 6.67 (s, 1H, C_5_-*H*), 7.32 (t, 2H, 2 × −C*H*- benzene ring), 8.12 (t, 1H, −C*H*- benzene ring), 8.69 (br s, 1H, -N*H*OH), 10.35 (s, 1H, -NHO*H*), 12.80 (s, 1H, -N*H-* uracil ring). ^13^C NMR (100 MHz, DMSO-*d*_6_) δ 24.9, 28.9, 30.1, 32.3, 104.3, 116.1 (2C), 116.3 (2C), 129.7 (2C), 129.8 (2C), 132.96.0,132.99, 159.5, 162.9, 164.1, 165.3, 169.3, 174.8. MS (ESI), *m*/*z*: 338 [M + H]^+^.

**MC1820** ***N*** **-hydroxy-5-((4-(3-methoxyphenyl)-6-oxo-1**,**6-dihydropyrimidin-2-yl)­thio)­pentanamide (11a)**. Mp: 160–162 °C; yield: 61%; rec. solv.: acetonitrile; ^1^H NMR (400 MHz, DMSO-*d*_6_) δ 1.63–1.70 (m, 4H, -SCH_2_C*H*_2_C*H*_2_CH_2_CO−), 1.97 (t, 2H, −C*H*_2_CO−), 3.22 (t, 2H, −C*H*_2_S-), 3.81 (s, 3H, -OC*H*_3_), 6.69 (s, 1H, C_5_-*H*), 7.04 (d, 1H, −C*H*- benzene ring), 7.39 (t, 1H, −C*H*- benzene ring), 7.60 (m, 2H, 2 × −C*H*- benzene ring), 8.71 (br s, 1H, -N*H*OH), 10.36 (s, 1H, -NHO*H*), 12.78 (s, 1H, -N*H-* uracil ring). ^13^C NMR (100 MHz, DMSO-*d*_6_) δ 24.9, 29.0, 30.1, 32.3, 55.6, 105.1, 112.2, 117.0, 119.6, 130.3,138.0, 160.0, 162.1, 163.9, 169.3, 176.3. MS (ESI), *m*/*z*: 350 [M + H]^+^.

**MC1819** ***N*** **-hydroxy-6-((4-(3-methoxyphenyl)-6-oxo-1**,**6-dihydropyrimidin-2-yl)­thio)­hexanamide (11b)**. Mp: 168–172 °C; yield: 63%; rec. solv.: acetonitrile; ^1^H NMR (400 MHz, DMSO-*d*_6_) δ 1.36–1.49 (m, 2H, −C*H*_2_CH_2_CH_2_S-), 1.51–1.53 (m, 2H, -*CH*_2_CH_2_CO−), 1.69–1.73 (m, 2H, -*CH*_2_CH_2_S-),1.94 (t, 2H, −C*H*_2_CO−), 3.20 (t, 2H, −C*H*_2_S-), 3.81 (s, 3H,-OC*H*_3_), 6.69 (s, 1H, C_5_-*H*), 7.05 (dd, 1H, −C*H*- benzene ring), 7.36 (t, 1H, −C*H*- benzene ring), 7.58–7.63 (m, 2H, 2 × −C*H*- benzene ring), 8.69 (br s, 1H, -N*H*OH), 10.35 (s, 1H, -NHO*H*), 12.66 (s, 1H, -N*H-* uracil ring). ^13^C NMR (100 MHz, DMSO-*d*_6_) δ 25.2, 28.4, 29.1, 30.3, 32.6, 55.6, 105.9, 112.3, 116.9, 119.5, 130.3, 138.0, 160.0, 160.5, 163.2, 169.4, 174.8. MS (ESI), *m*/*z*: 364 [M + H]^+^.

**MC1821** ***N*** **-hydroxy-5-((4-(4-methoxyphenyl)-6-oxo-1**,**6-dihydropyrimidin-2-yl)­thio)­pentanamide (12a)**. Mp: 142–144 °C; yield: 59%; rec. solv.: benzene; ^1^H NMR (400 MHz, DMSO-*d*_6_) δ 1.62–1.70 (m, 4H, -SCH_2_C*H*_2_C*H*_2_CH_2_CO−), 1.99 (t, 2H, −C*H*_2_CO−), 3.22 (t, 2H, −C*H*_2_S-), 3.81 (s, 3H, -OC*H*_3_), 6.57 (s, 1H, C_5_-*H*), 7.03 (d, 2H, 2 × −C*H*- benzene ring), 8.19 (d, 2H, 2 × −C*H*- benzene ring), 8.69 (br s, 1H, -N*H*OH), 10.35 (s, 1H, -NHO*H*), 12.66 (s, 1H, -N*H-* uracil ring). ^13^C NMR (100 MHz, DMSO-*d*_6_) δ 24.9, 28.9, 30.0, 32.3, 55.8, 102.8, 114.6 (2C), 128.8, 128.9 (2C), 160.3,161.8, 164.3, 169.3, 174.8. MS (ESI), *m*/*z*: 350 [M + H]^+^.

**MC1818** ***N*** **-hydroxy-6-((4-(4-methoxyphenyl)-6-oxo-1**,**6-dihydropyrimidin-2-yl)­thio)­hexanamide (12b)**. Mp: 168–170 °C; yield: 63%; rec. solv.: methanol; ^1^H NMR (400 MHz, DMSO-*d*_6_) δ 1.38–1.39 (m, 2H, −C*H*_2_CH_2_CH_2_S-), 1.52–1.56 (m, 2H, -*CH*_2_CH_2_CO−), 1.69–1.72 (m, 2H, -*CH*_2_CH_2_S-),1.95 (t, 2H, −C*H*_2_CO−), 3.20 (t, 2H, −C*H*_2_S-), 3.81 (s, 3H, -OC*H*_3_), 6.57 (s, 1H, C_5_-*H*), 7.03 (d, 2H, 2 × −C*H*- benzene ring), 8.02 (d, 2H, 2 × −C*H*- benzene ring), 8.69 (br s, 1H, -N*H*OH), 10.35 (s, 1H, -NHO*H*), 12.55 (s, 1H, -N*H-* uracil ring). ^13^C NMR (100 MHz, DMSO-*d*_6_) δ 24.9, 28.9, 30.1, 32.1, 32.3, 55.9, 103.0, 114.7 (2C), 128.9, 129.1 (2C), 160.5, 161.9, 164.4, 169.4, 175.0. MS (ESI), *m*/*z*: 364 [M + H]^+^.

**MC1930 5-((4-([1**,**1′-biphenyl]-3-yl)-6-oxo-1**,**6-dihydropyrimidin-2-yl)­thio)-** ***N*** **-hydroxypentanamide (13a)**. Mp: 198–200 °C; yield: 52%; rec. solv.: methanol; ^1^H NMR (400 MHz, DMSO-*d*_6_) δ 1.67–1.73 (m, 4H, -SCH_2_C*H*_2_C*H*_2_CH_2_CO−), 2.02 (t, 2H, −C*H*_2_CO−), 3.26 (t, 2H, −C*H*_2_S-), 6.73 (s, 1H, C_5_-*H*), 7.28–8.26 (m, 9H, 9 × −C*H*- 3′-biphenyl ring), 8.71 (br s, 1H, -N*H*OH), 10.38 (s, 1H, -NHO*H*), 12.63 (s, 1H, -N*H-* uracil ring). ^13^C NMR (100 MHz, DMSO-*d*_6_) δ 25.1, 28.2, 29.7, 32.6, 110.5, 127.3, 128.0, 128.7 (2C), 129.2 (2C), 130.1, 130.2, 131.1, 137.3, 140.8, 141.5, 162.6, 164.1, 169.4, 175.2. MS (ESI), *m*/*z*: 396 [M + H]^+^.

**MC1931 6-((4-([1**,**1′-biphenyl]-3-yl)-6-oxo-1**,**6-dihydropyrimidin-2-yl)­thio)-** ***N*** **-hydroxyhexanamide (13b)**. Mp: 188–190 °C; yield: 56%; rec. solv.: methanol; ^1^H NMR (400 MHz, DMSO-*d*_6_) δ 1.17–1.21 (m, 2H, −C*H*_2_CH_2_CH_2_S-), 1.33–1.34 (m, 2H, -*CH*_2_CH_2_CO−), 1.41–1.43 (m, 2H, -*CH*_2_CH_2_S-),1.89 (t, 2H, −C*H*_2_CO−), 2.57 (t, 2H, −C*H*_2_S-), 6.01 (s, 1H, C_5_-*H*), 7.20–7.59 (m, 9H, 9 × −C*H*- 3′-biphenyl ring), 8.66 (br s, 1H, -N*H*OH), 10.31 (s, 1H, -NHO*H*), 12.58 (s, 1H, -N*H-* uracil ring). ^13^C NMR (100 MHz, DMSO-*d*_6_) δ 25.1, 28.2, 28.5, 29.7, 32.6, 109.5, 127.3, 128.0, 128.7 (2C), 129.2 (2C),130.1, 130.2, 131.1, 137.3, 140.8, 141.5, 162.0, 164.3, 169.4, 174.9. MS (ESI), *m*/*z*: 410 [M + H]^+^.

**MC1739** ***N*** **-hydroxy-5-((4-(naphthalen-1-yl)-6-oxo-1**,**6-dihydropyrimidin-2-yl)­thio)­pentanamide (15a)**. Mp: 182–184 °C; yield: 48%; rec. solv.: methanol; ^1^H NMR (400 MHz, DMSO-*d*_6_) δ 1.55–1.57 (m, 2H, -SCH_2_CH_2_C*H*_2_CH_2_CO−), 1.63–1.65 (m, 2H, -SCH_2_C*H*_2_C*H*_2_CH_2_CO−), 1.94 (t, 2H, −C*H*_2_CO−), 3.09 (t, 2H, −C*H*_2_S-), 6.34 (s, 1H, C_5_-*H*), 7.58–7.67 (m, 4H, 4 × −C*H*- 1-naphtyl ring), 8.01–8.05 (m, 2H, 2 × −C*H*- 1-naphtyl ring), 8.17 (d, 1H, −C*H*- 1-naphtyl ring), 8.69 (br s, 1H, -N*H*OH), 10.34 (s, 1H, -NHO*H*), 12.86 (s, 1H, -N*H-* uracil ring). ^13^C NMR (100 MHz, DMSO-*d*_6_) δ 24.7, 29.1, 29.9, 32.3, 104.7, 125.80, 125.85, 126.7, 127.1, 127.6,128.8, 130.2, 130.4, 133.8, 136.3, 163.1, 165.7, 169.2, 174.9. MS (ESI), *m*/*z*: 370 [M + H]^+^.

**MC1737** ***N*** **-hydroxy-6-((4-(naphthalen-1-yl)-6-oxo-1**,**6-dihydropyrimidin-2-yl)­thio)­hexanamide (15b)**. Mp: 198–200 °C; yield: 54%; rec. solv.: methanol; ^1^H NMR (400 MHz, DMSO-*d*_6_) δ 1.23–1.29 (m, 2H, −C*H*_2_CH_2_CH_2_S-), 1.45–1.51 (m, 2H, -*CH*_2_CH_2_CO−), 1.62–1.67 (m, 2H, -*CH*_2_CH_2_S-),1.90 (t, 2H, −C*H*_2_CO−), 3.09 (t, 2H, −C*H*_2_S-), 6.34 (s, 1H, C_5_-*H*), 7.53–7.66 (m, 4H, 4 × −C*H*- 1-naphtyl ring), 8.01–8.05 (m, 2H, 2 × −C*H*- 1-naphtyl ring), 8.16 (d, 1H, −C*H*- 1-naphtyl ring), 8.66 (br s, 1H, -N*H*OH), 10.32 (s, 1H, -NHO*H*), 12.83 (s, 1H, -N*H-* uracil ring). ^13^C NMR (100 MHz, DMSO-*d*_6_) δ 25.1, 28.2, 29.2, 30.1, 32.6, 111.1, 125.7, 125.8, 126.6, 126.9, 127.4, 128.8, 130.1, 130.4, 133.7, 136.2, 161.3, 163.2, 169.4, 174.8. MS (ESI), *m*/*z*: 384 [M + H]^+^.

**MC1850 5-((4-cyclohexyl-6-oxo-1**,**6-dihydropyrimidin-2-yl)­thio)-** ***N*** **-hydroxypentanamide (17a)**. Mp: 175–179 °C; yield: 51%; rec. solv.:methanol; ^1^H NMR (400 MHz, DMSO-*d*_6_) δ 1.15–1.41 (m, 5H, -SCH_2_C*H*_2_C*H*_2_CH_2_CO- + −CH- cyclohexyl ring), 1.59–1.75 (m, 9H, 9 × −C*H*- cyclohexyl ring), 1.96 (t, 2H, −C*H*_2_CO−), 2.29–2.32 (m, 1H, −C*H*- cyclohexyl ring), 3.09 (t, 2H, −C*H*_2_S-), 5.86 (s, 1H, C_5_-*H*), 8.69 (br s, 1H, -N*H*OH), 10.34 (s, 1H, -NHO*H*), 12.78 (s, 1H, -N*H-* uracil ring).^13^C NMR (100 MHz, DMSO-*d*_6_) δ 24.8, 26.0, 26.1 (2C), 29.1, 29.8, 31.3 (2C), 32.3, 44.9, 105.2, 161.9, 164.3,169.2, 171.7. MS (ESI), *m*/*z*: 326 [M + H]^+^.

**MC1851 6-((4-cyclohexyl-6-oxo-1**,**6-dihydropyrimidin-2-yl)­thio)-** ***N*** **-hydroxyhexanamide (17b)**. Mp: 172–174 °C; yield: 49%; rec. solv.:methanol; ^1^H NMR (400 MHz, DMSO-*d*_6_) δ 1.15–1.41 (m, 9H, -SCH_2_C*H*_2_ *CH*_2_C*H*_2_CH_2_CO- + 3 × −CH_2_– cyclohexyl ring ring), 1.59–1.75 (m, 7H, 7 × −C*H*- cyclohexyl ring), 1.93 (t, 2H, −C*H*_2_CO−), 2.30–2.34 (m, 1H, −C*H*- cyclohexyl ring), 3.07 (t, 2H, −C*H*_2_S-), 5.87 (s, 1H, C_5_-*H*), 8.65 (br s, 1H, -N*H*OH), 10.32 (s, 1H, -NHO*H*), 12.37 (s, 1H, -N*H-* uracil ring). ^13^C NMR (100 MHz, DMSO-*d*_6_) δ 25.0, 26.28 (2C), 26.33 (2C), 29.3, 30.1, 31.0, 31.6 (2C), 32.6, 45.3, 106.1, 163.3, 165.4,170.6, 173.2. MS (ESI), *m*/*z*: 340 [M + H]^+^.

**MC1852** ***N*** **-hydroxy-6-((4-(2-methylbenzyl)-6-oxo-1**,**6-dihydropyrimidin-2-yl)­thio)­hexanamide (18)**. Mp: 163–166 °C; yield: 49%; rec. solv.: acetonitrile/methanol; ^1^H NMR (400 MHz, DMSO-*d*_6_) δ 1.23–1.26 (m, 2H, −C*H*_2_CH_2_CH_2_S-), 1.43–1.50 (m, 4H, -SCH_2_C*H*_2_CH_2_C*H*_2_CH_2_CO−), 1.92 (t, 2H, −C*H*_2_CO−), 2.66 (s, 3H, Ph–C*H*_3_), 2.98 (t, 2H, −C*H*_2_S-), 3.76 (s, 2H, Ph–C*H*_2_-), 5.78 (s, 1H, C_5_-*H*), 7.10–7.19 (m, 4H, 4 × −C*H*- benzene ring), 8.67 (br s, 1H, -N*H*OH), 10.33 (s, 1H, -NHO*H*), 12.58 (s, 1H, -N*H-* uracil ring).^13^C NMR (100 MHz, DMSO-*d*_6_) δ 19.8, 25.1, 28.2, 29.2, 29.9, 32.6, 40.8, 108.3, 126.3, 127.2, 130.5, 130.7, 136.6, 136.9, 163.9, 166.9, 169.4, 174.9. MS (ESI), *m*/*z*: 362 [M + H]^+^.

**MC1849** ***N*** **-hydroxy-6-((4-(3-methylbenzyl)-6-oxo-1**,**6-dihydropyrimidin-2-yl)­thio)­hexanamide (19)**. Mp: 164–167 °C; yield: 46%; rec. solv.:methanol; ^1^H NMR (400 MHz, DMSO-*d*_6_) δ 1.27–1.28 (m, 2H, −C*H*_2_CH_2_CH_2_S-), 1.45–1.55 (m, 4H, -SCH_2_C*H*_2_CH_2_C*H*_2_CH_2_CO−), 1.92 (t, 2H, −C*H*_2_CO−), 2.26 (s, 3H, Ph–C*H*_3_), 3.03 (t, 2H, −C*H*_2_S-), 3.68 (s, 2H, Ph–C*H*_2_-), 5.91 (s, 1H, C_5_-*H*), 7.01–7.19 (m, 4H, 4 × −C*H*- benzene ring), 8.68 (br s, 1H, -N*H*OH), 10.33 (s, 1H, -NHO*H*), 12.54 (s, 1H, -N*H-* uracil ring). ^13^C NMR (100 MHz, DMSO-*d*_6_) δ 21.5, 25.1, 28.2, 29.1, 30.0, 32.6, 43.3, 108.3, 126.8, 127.6, 128.7,130.3, 137.8, 138.3, 163.1, 166.0, 169.4, 176.2. MS (ESI), *m*/*z*: 362 [M + H]^+^.

**MC1848** ***N*** **-hydroxy-6-((4-(4-methylbenzyl)-6-oxo-1**,**6-dihydropyrimidin-2-yl)­thio)­hexanamide (20)**. Mp: 171–173 °C; yield: 55%; rec. solv.:methanol; ^1^H NMR (400 MHz, DMSO-*d*_6_) δ 1.21–1.28 (m, 2H, −C*H*_2_CH_2_CH_2_S-), 1.44–1.52 (m, 4H, -SCH_2_C*H*_2_CH_2_C*H*_2_CH_2_CO−), 1.91 (t, 2H, −C*H*_2_CO−), 2.25 (s, 3H, Ph–C*H*_3_), 3.00 (t, 2H, −C*H*_2_S-), 3.66 (s, 2H, Ph–C*H*_2_-), 5.90 (s, 1H, C_5_-*H*), 7.11 (dd, 4H, 4 × −C*H*- benzene ring), 8.68 (br s, 1H, -N*H*OH), 10.34 (s, 1H, -NHO*H*), 12.50 (s, 1H, -N*H-* uracil ring). ^13^C NMR (100 MHz, DMSO-*d*_6_) δ 21.1, 25.1, 28.3, 29.2, 29.9, 32.6, 42.9, 108.0, 129.4 (2C), 129.6 (2C), 135.3,135.9, 163.0, 167.0, 169.4, 174.9. MS (ESI), *m*/*z*: 362 [M + H]^+^.

**MC1847 6-((4-(2-chlorobenzyl)-6-oxo-1**,**6-dihydropyrimidin-2-yl)­thio)-** ***N*** **-hydroxyhexanamide (21)**. Mp: 158–160 °C; yield: 56%; rec. solv.: acetonitrile/methanol; ^1^H NMR (400 MHz, DMSO-*d*_6_) δ 1.21–1.23 (m, 2H, −C*H*_2_CH_2_CH_2_S-), 1.42–1.47 (m, 4H, -SCH_2_C*H*_2_CH_2_C*H*_2_CH_2_CO−), 1.92 (t, 2H, −C*H*_2_CO−),2.95 (t, 2H, −C*H*_2_S-), 3.89 (s, 2H, Ph–C*H*_2_-), 5.81 (s, 1H, C_5_-*H*), 7.29–7.44 (m, 4H, 4 × −C*H*- benzene ring), 8.70 (br s, 1H, -N*H*OH), 10.33 (s, 1H, -NHO*H*), 12.52 (s, 1H, -N*H-* uracil ring). ^13^C NMR (100 MHz, DMSO-*d*_6_) δ 25.1, 28.2, 29.2, 29.9, 32.6, 41.2, 107.6, 127.7, 129.1, 129.7, 132.6, 134.0, 135.8, 164.0, 165.1, 169.4, 175.0. MS (ESI), *m*/*z*: 382 [M + H]^+^.

**MC1840 6-((4-(3-chlorobenzyl)-6-oxo-1**,**6-dihydropyrimidin-2-yl)­thio)-** ***N*** **-hydroxyhexanamide (22)**. Mp: 162–165 °C; yield: 61%; rec. solv.: methanol; ^1^H NMR (400 MHz, DMSO-*d*_6_) δ 1.26–1.27 (m, 2H, −C*H*_2_CH_2_CH_2_S-), 1.44–1.53 (m, 4H, -SCH_2_C*H*_2_CH_2_C*H*_2_CH_2_CO−), 1.92 (t, 2H, −C*H*_2_CO−), 3.01 (t, 2H, −C*H*_2_S-), 3.75 (s, 2H, Ph–C*H*_2_-), 5.99 (s, 1H, C_5_-*H*), 7.23–7.37 (m, 4H, 4 × −C*H*- benzene ring), 8.68 (br s, 1H, -N*H*OH), 10.34 (s, 1H, -NHO*H*), 12.58 (s, 1H, -N*H-* uracil ring). ^13^C NMR (100 MHz, DMSO-*d*_6_) δ 25.1, 28.2, 29.2, 30.0, 32.6, 42.6, 108.4, 126.9, 128.5, 129.6,130.6, 133.3, 140.9, 161.2, 165.7, 169.4, 176.1. MS (ESI), *m*/*z*: 382 [M + H]^+^.

**MC1846 6-((4-(4-chlorobenzyl)-6-oxo-1**,**6-dihydropyrimidin-2-yl)­thio)-** ***N*** **-hydroxyhexanamide (23)**. Mp: 172–174 °C; yield: 59%; rec. solv.: methanol; ^1^H NMR (400 MHz, DMSO-*d*_6_) δ 1.26–1.29 (m, 2H, −C*H*_2_CH_2_CH_2_S-), 1.43–1.53 (m, 4H, -SCH_2_C*H*_2_CH_2_C*H*_2_CH_2_CO−), 1.93 (t, 2H, −C*H*_2_CO−), 3.01 (t, 2H, −C*H*_2_S-), 3.74 (s, 2H, Ph–C*H*_2_-), 5.96 (s, 1H, C_5_-*H*), 7.32 (dd, 4H, 4 × −C*H*- benzene ring), 8.66 (br s, 1H, -N*H*OH), 10.33 (s, 1H, -NHO*H*), 12.50 (s, 1H, -N*H-* uracil ring). ^13^C NMR (100 MHz, DMSO-*d*_6_) δ 25.1, 28.2, 29.2, 30.0, 32.6, 42.4, 108.2, 128.7 (2C), 131.6 (2C), 131.7,137.5, 162.0, 166.0, 169.4, 174.9. MS (ESI), *m*/*z*: 382 [M + H]^+^.

**MC1867 6-((4-([1**,**1′-biphenyl]-4-ylmethyl)-6-oxo-1**,**6-dihydropyrimidin-2-yl)­thio)-** ***N*** **-hydroxyhexanamide (24)**. Mp: 208–210 °C; yield: 47%; rec. solv.: ethanol; ^1^H NMR (400 MHz, DMSO-*d*_6_) δ 1.28–1.31 (m, 2H, −C*H*_2_CH_2_CH_2_S-), 1.45–1.56 (m, 4H, -SCH_2_C*H*_2_CH_2_C*H*_2_CH_2_CO−), 1.91 (t, 2H, −C*H*_2_CO−), 3.05 (t, 2H, −C*H*_2_S-), 3.78 (s, 2H, Ph–C*H*_2_-), 5.99 (s, 1H, C_5_-*H*), 7.32–7.46 (m, 5H, 5 × −C*H*- 4′-biphenyl ring), 7.59–7.65 (m, 4H, 4 × −C*H*- 4′-biphenyl ring), 8.68 (br s, 1H, -N*H*OH), 10.34 (s, 1H, -NHO*H*), 12.62 (s, 1H, -N*H-* uracil ring). ^13^C NMR (100 MHz, DMSO-*d*_6_) δ 25.1, 28.2, 29.2, 30.0, 32.6, 42.9, 108.2, 127.0, 127.1, 127.8, 129.4, 130.3, 137.7, 138.9, 140.4, 163.0, 166.6, 169.4, 174.9. MS (ESI), *m*/*z*: 424 [M + H]^+^.

**MC1861** ***N*** **-hydroxy-6-((4-(naphthalen-1-ylmethyl)-6-oxo-1**,**6-dihydropyrimidin-2-yl)­thio)­hexanamide (25)**. Mp: 172–174 °C; yield: 51%; rec. solv.: methanol; ^1^H NMR (400 MHz, DMSO-*d*_6_) δ 1.15–1.18 (m, 2H, −C*H*_2_CH_2_CH_2_S-), 1.38–1.42 (m, 4H, -SCH_2_C*H*_2_CH_2_C*H*_2_CH_2_CO−), 1.91 (t, 2H, −C*H*_2_CO−), 2.93 (t, 2H, −C*H*_2_S-), 4.24 (s, 2H, Ph–C*H*_2_-), 5.99 (s, 1H, C_5_-*H*), 7.48–7.52 (m, 4H, 4 × −C*H*- 1-naphtyl ring), 7.82–7.84 (m, 1H, −C*H*- 1-naphtyl ring), 7.92 (d, 1H, −C*H*- 1-naphthyl ring), 8.10 (m, 1H, −C*H*- 1-naphtyl ring), 8.67 (br s, 1H, -N*H*OH), 10.34 (s, 1H, -NHO*H*), 12.64 (s, 1H, -N*H-* uracil ring). ^13^C NMR (100 MHz, DMSO-*d*_6_) 25.1, 28.2, 29.1, 29.9, 32.6, 43.8, 108.4, 124.9, 126.0, 126.1, 126.5, 127.7, 128.5, 128.9, 132.2, 133.9, 134.5, 161.2, 166.3, 169.4, 176.5. MS (ESI), *m*/*z*: 398 [M + H]^+^.

**MC1866** ***N*** **-hydroxy-6-((4-(naphthalen-2-ylmethyl)-6-oxo-1**,**6-dihydropyrimidin-2-yl)­thio)­hexanamide (26)**. Mp: 160–162 °C; yield: 52%; rec. solv.: acetonitrile/methanol; ^1^H NMR (400 MHz, DMSO-*d*_6_) δ 1.19–1.23 (m, 2H, −C*H*_2_CH_2_CH_2_S-), 1.37–1.50 (m, 4H, -SCH_2_C*H*_2_CH_2_C*H*_2_CH_2_CO−), 1.87 (t, 2H, −C*H*_2_CO−), 3.00 (t, 2H, −C*H*_2_S-), 3.91 (s, 2H, Ph–C*H*_2_-), 6.01 (s, 1H, C_5_-*H*), 7.43–7.46 (m, 3H, 3 × −C*H*-2-naphtyl ring), 7.78 (s, 1H, −C*H*- 2-naphtyl ring), 7.84–7.86 (m, 3H, 3 × −C*H*-2-naphtyl ring), 8.69 (br s, 1H, -N*H*OH), 10.33 (s, 1H, -NHO*H*), 12.59 (s, 1H, -N*H-* uracil ring). ^13^C NMR (100 MHz, DMSO-*d*_6_) δ 25.0, 28.2, 29.2, 30.0, 32.6, 43.4, 108.5, 126.1, 126.6, 127.9,127.96, 127.99, 128.2, 128.3, 132.3, 133.5, 136.1, 161.2, 163.2, 169.4, 174.9. MS (ESI), *m*/*z*: 398 [M + H]^+^.

##### 4.1.2.5 3-([1,1′-Biphenyl]-4-yl)-3-aminoacrylamide (**132**)

Ethyl 4-phenylbenzoylacetate (4.02 g, 15 mmol) and aqueous ammonia (12.77 mL, 28%) were heated together at 120 °C for 2 h in a stainless-steel bomb. After cooling, the mixture was poured into water (20 mL) and refrigerated for 1 h. The product was filtered, dried and purified by crystallization.

Mp: 138–140 °C; yield: 58%; rec. solv.: acetonitrile; ^1^H NMR (400 MHz, CDCl_3_) δ 4.91 (s, 2H, -N*H*_2_C=CHCO−), 5.01 (s, 1H, -NH_2_C=C*H*-), 6.90 (s, 2H, −CON*H*_2_), 7.45–7.49 (m, 3H, 3 × −C*H*- benzene ring), 7.60–7.63 (m, 6H, 6 × −C*H*- benzene ring).

##### 4.1.2.6 6-(4-([1,1′-Biphenyl]-4-yl)-6-oxo-1,6-dihydropyrimidin-2-yl)­hexanoic Acid (**133**)

Sodium metal (0.78 g, 33.9 mmol, 5 equiv) was dissolved in 14 mL of absolute ethanol, and the 3-([1,1′-biphenyl]-4-yl)-3-aminoacrylamide **132** (2.46 g, 6.78 mmol, 1 equiv) and diethyl pimelate (2.90 g, 13.56 mmol, 2 equiv) were added to the clear solution. The mixture was heated at reflux for 6 h. After the completion of reaction the mixture was cooled, the solvent was distilled in vacuo at 40–50 °C until dry and the residue was dissolved in water (10 mL). The aqueous phase was extracted with ethyl acetate (3 × 10 mL). HCl 2N was then added to the aqueous layer until the pH was 2, and the precipitate was filtered and recrystallized from tetrahydrofuran/methanol to yield the title compound **133** as a pure white solid.

Mp: 239–241 °C; yield: 67%; rec. solv.: tetrahydrofuran/methanol;^1^H NMR (400 MHz, DMSO-*d*_6_) δ 1.32–1.34 (m, 2H, −CH_2_CH_2_C*H*_2_CH_2_CH_2_CO−), 1.51–1.53 (m, 2H, −CH_2_CH_2_CH_2_C*H*_2_CH_2_CO−), 1.72–1.75 (m, 2H, −CH_2_C*H*_2_CH_2_CH_2_CH_2_CO−), 2.14 (t, 2H, −C*H*_2_CO−), 2.63 (t, 2H, −C*H*_2_CH_2_CH_2_CH_2_CH_2_CO−), 6.76 (s, 1H, C_5_-*H*), 7.37–7.50 (m, 3H, 3 × −C*H*- 4′-biphenyl ring), 7.68–7.77 (m, 4H, 4 × −C*H*- 4′-biphenyl ring), 8.12–8.14 (m, 2H, 2 × −C*H*- 4′-biphenyl ring), 10.28 (s, 1H, -NHO*H*), 12.48 (br s, 2H, OH + -N*H-* uracil ring).

##### 4.1.2.7 Procedure for the Synthesis of 6-(4-([1,1′-Biphenyl]-4-yl)-6-oxo-1,6-dihydropyrimidin-2-yl)-*N*-hydroxyhexanamide (MC2026–27)

Compound **27** was prepared in a similar manner to compounds **1a**–**26**. Triethylamine (3.74 mmol, 0.52 mL, 2.6 equiv) and ethyl chloroformate (3.46 mmol, 0.33 mL, 2.4 equiv) were added in sequence to a 0 °C cooled solution of intermediate **133** (1.44 mmol, 522 mg, 1 equiv) in anhydrous THF (8 mL), and the resulting mixture was stirred at room temperature for 10 min. The obtained white solid was quickly filtered off and commercially *O*-(2-methoxy-2-propyl)­hydroxylamine (8.64 mmol, 0.64 mL, 6 equiv) was added to the filtrate while cooling at 0 °C. The resulting mixture was then stirred at room temperature for 3 h, evaporated under reduced pressure, and the residue dissolved in methanol (5 mL). Amberlyst 15 ion-exchange resin (290 mg) was then added to the solution of the *O*-protected hydroxamate, and the resulting mixture was stirred at room temperature for 1.5 h. The reaction mixture was then filtered to remove the resin, and the filtrate was concentrated under vacuum to yield the crude compound **27**, which was finally purified by crystallization from methanol.

Mp: 213–215 °C; yield: 54%; rec. solv.: methanol; ^1^H NMR (400 MHz, DMSO-*d*_6_) δ 1.31–1.33 (m, 2H, −CH_2_CH_2_C*H*_2_CH_2_CH_2_CO−), 1.52–1.56 (m, 2H, −CH_2_CH_2_CH_2_C*H*_2_CH_2_CO−), 1.72–1.75 (m, 2H, −CH_2_C*H*_2_CH_2_CH_2_CH_2_CO−),1.95 (t, 2H, −C*H*_2_CO−), 2.60 (t, 2H, −C*H*_2_CH_2_CH_2_CH_2_CH_2_CO−), 6.77 (s, 1H, C_5_-*H*), 7.39–7.41 (m, 1H, −C*H*- 4′-biphenyl ring), 7.47–7.51 (m, 2H, 2 × −C*H*- 4′-biphenyl ring), 7.72–7.79 (m, 4H, 4 × −C*H*- 4′-biphenyl ring), 8.11–8.15 (m, 2H, 2 × −C*H*- 4′-biphenyl ring), 8.66 (br s, 1H, -N*H*OH), 10.28 (s, 1H, -NHO*H*), 12.52 (s, 1H, -N*H-* uracil ring). ^13^C NMR (100 MHz, DMSO-*d*_6_) δ 25.3, 26.9, 28.5, 32.6, 34.7, 107.1, 127.2 (2C), 127.3, 128.0, 128.4,129.5 (2C), 135.8, 139.8, 142.4, 160.5, 163.4, 169.5, 174.9. MS (ESI), *m*/*z*: 378 [M + H]^+^.

### 4.2 HDAC1–11 Inhibition Assays

The described compounds were tested against purified hrHDAC1, 3, 4, 6, and 8 in a 10-dose IC_50_ mode with 3-fold serial dilution starting from 100 μM solutions. Three fluorogenic peptides, from p53 residues 379–382 (RHKK­(Ac)­AMC) (for HDAC1–3, 6, 10), or the fluorogenic class IIa (Boc-Lys­(trifluoroacetyl)-AMC) substrate (for HDAC4, 5, 7, 9 and 11)^63^ the diacetylated p53 residues 379–382 (RHK­(Ac)­K­(Ac) AMC) (for HDAC8) were used as substrates, and TSA was employed as reference compound and positive control. Deacetylated peptides were sensitive toward lysine peptidase, and free fluorogenic 4-methylcoumarin-7-amide (MCA) was generated, which can be excited at 355 nm and observed at 460 nm. The data were analyzed on a plate-to-plate basis in relation to the control and imported into analytical software. IC_50_ values were calculated using GraphPad Prism 8 based on a sigmoidal dose–response equation.

### 4.3 Cell Culture

HL60, U937, NB4, MV4:11, IMS-M2, and Karpass299 cells were maintained in RPMI-1640 medium supplemented with 10% bovine serum (FBS, Euroclone Milan IT), 2 mM l-glutamine, 100 U/mL penicillin, and 100 μg/mL streptomycin (Euroclone) at 37 °C in a humidified atmosphere containing 5% CO_2_.

Human lung carcinoma (H1299), glioblastoma (ADF), breast adenocarcinoma (MCF7), melanoma (M14), ovarian carcinoma (OVCA3), colorectal carcinoma (HT29), fibroblastoma (HT1080), and B lymphocyte AHH1 cell line were maintained in RPMI-1640 medium supplemented with 10% FBS and antibiotics. Human fibroblasts (BJ) and endothelial cells (EA.hy926), were maintained in DMEM medium supplemented with 10% fetal bovine serum and antibiotics. Mammary epithelial cells (HME) were cultured in Human Mammary Epithelial Cell Basal Medium (Lonza) supplemented with Bovine Pituitary Extract (0.004 mL/ml), recombinant human Epidermal Growth Factor (10 ng/mL), Insulin (5 μg/mL), Hydrocortisone (0.5 μg/mL), and antibiotics.

OCI-AML3 and Kasumi-1 cells were grown in RPMI-1640 with 20% FBS, 2 mM l-glutamine, 100 U/mL penicillin, 100 μg/mL streptomycin. OCI-AML2 cells were propagated in MEM-α supplemented with 20% FBS, 2 mM l-glutamine, and 100 U/mL penicillin, 100 μg/mL streptomycin. All reagents were purchased from Euroclone (Italy).

PC-3M-luc2 and 22Rv1-luc cells were grown in MEM (Corning, #15-010-CV) and RPMI medium (1640 Corning, #10-040-CV), respectively, supplemented with 10% FBS (GIBCO, #10270106), 1% glutamine (Corning #25,005-CI), 1% penicillin and streptomycin (Corning #30002-CI). Medium for 22Rv1-luc cells was supplemented with 1% HEPES (Corning #25060-CI), 1% sodium pyruvate (Corning #25000-CIR), and 1% glucose. Cells were incubated at 37 °C with 5% CO_2_. The genetic identity of PC-3M-luc2 and 22Rv1-luc cell lines were authenticated by BMR Genomics (Padova, Italy) in October 2022. Indirect (Hoechst) methods routinely screened all cell lines for mycoplasma contamination.

### 4.4 Cell Viability and Antiproliferative Activity Assays

The influence on cell viability of selected compounds against U937 and HL60 cell lines was evaluated using the CellTiter-Glo luminescent cell viability assay (Promega, Madison, WI, USA) according to the manufacturer’s instructions. Cells were incubated with various inhibitor concentrations. An equivalent of the CellTiter-Glo reagent was then added, the solution was mixed for 2 min in order to induce cell lysis, and the luminescence was recorded after a further 10 min. The antiproliferative effect of selected compounds against solid tumor cell lines and nontransformed cell lines was evaluated by 3-(4,5-dimethylthiazol-2-yl)-2,5-diphenyltetrazolium bromide (MTT, mitochondrial respiration analysis, Sigma–Aldrich), as previously reported.^64^ CC_50_ values were calculated using GraphPad 8 software.

The effect on cell viability of the selected compounds for OCI-AML2, OCI-AML3, U937, NB4, HL-60, MV4:11, IMS-M2, and Karpass299 cells was evaluated using the WST-1 assay (Roche Diagnostic GmbH Germany). A total amount of 5 × 10^3^ cells was plated in 22.5 μL of medium in 384-viewplate (PerkinElmer USA). Cells were stimulated in triplicate, with increasing concentrations of compounds (dose–response from 10^–9^ M to 10^–5^ M) and incubated at 37 °C, 5% CO_2_ for 48 h then 2.5 μL of WST-1 reagent was added in each well and incubated for an additional 45 min at 37 °C. Dose–response curves were analyzed using GraphPad Prism 8.0 as nonlinear regression curves variable slope–four parameters to calculate the CC_50_ values for each compound in each cell line.

PC-3M-luc2 and 22Rv1-luc cells were treated with **14a** and SAHA for 70 h at the concentrations as indicated in the legend to figures. DMSO 0.5% (v/v) was used as a control. PC-3M-luc2 and 22Rv1-luc cells were seeded at 1500 cells/well and 2500 cell/well respectively, in sextuplicate in a 96-well plate containing complete medium. After 24 h, cells were exposed to drugs for 72 h. Proliferation was assessed using the IncuCyte system S3 Kinetic Live Cell Imaging System (Sartorius, Essen BioScience, Ann Arbor, MI, USA) at 37 °C with 5% CO_2_. Images, at 10× magnification, were acquired every 2 h and analyzed using the IncuCyte Cell-by-Cell software (version 2022b rev2) to detect and quantify live cells. Statistical analysis was performed using a Student’s *t* test (*p < 0.05; ** *p* < 0.01). Control consists of 0.5% (v/v) DMSO-treated cells.

### 4.5 Caco-2 Permeability Assay

Caco-2 cells (American Type Culture Collection, ATCC HTB-37, Manassas, VA, USA), supplied by LGC Standards S.r.l. (Milan, Italy), were cultured at an initial seeding density of approximately 2 × 10^4^ cells/cm^2^ in Dulbecco’s Modified Eagle’s Medium (DMEM), supplemented with 10% (v/v) fetal bovine serum, 2 mM l-glutamine, 100 μg/mL streptomycin, and 100 U/mL penicillin, and maintained under standard culture conditions (37 °C, 5% CO_2_). The culture medium was renewed twice weekly. Subculturing was performed when cells reached approximately 80% confluence. All experiments related to cell expansion were performed during the logarithmic growth phase, prior to differentiation.

For the permeability studies, Caco-2 cells at confluence were seeded onto 24-well PET Transwell inserts (0.33 cm^2^ surface area, 0.4 μm pore size; Merck Millipore, Merck KGaA, Darmstadt, Germany) at a density of 6 × 10^4^ cells/cm^2^ and allowed to differentiate for 21 days under a humidified atmosphere of 5% CO_2_ at 37 °C, according to previous published methods.^65^ The culture medium was replaced every two to 3 days. Fully differentiated monolayers exhibiting transepithelial electrical resistance (TEER) values greater than 300 Ω·cm^2^ were selected for subsequent permeability experiments. In order to assess intestinal barrier integrity, the cells were washed twice with 1× Hank’s Balanced Salt Solution (HBSS) to remove residual serum components and then treated in the apical compartment to compounds **14a** and **14b** at a final concentration of 20 μM in serum-free culture medium for 2 h. Dimethyl sulfoxide (DMSO) was used as the solvent for the tested compounds at a final concentration of 0.5% (v/v), which did not affect monolayer integrity or cell viability. Ethanol 50% v/v in serum-free culture medium was used as a positive control to verify the ability of the experimental model to detect disruption of Caco-2 monolayer integrity. DMSO-treated wells served as the negative control, whereas 50% ethanol was included as a positive control for barrier disruption.

After 2 h, basolateral samples were collected for quantification. In parallel, apical samples were collected at the end of the incubation for mass-balance assessment. TEER was recorded after compound exposure using a Millicell ERS 3.0 Digital Voltohmmeter (Merck Millipore) and expressed as Ω·cm^2^ after normalization to the inset surface area. Each experimental condition was tested in at least three inserts per experiment and in a minimum of two independent experiments. Collected apical and basolateral samples were processed by centrifugation, followed by lyophilization and reconstitution in HPLC Solvent A prior to analysis. Specifically, basolateral samples (1.5 mL) were lyophilized and reconstituted in 0.3 mL Solvent A (5× concentration). Control solutions (20 μM compound in culture medium) were processed in parallel and concentrated 5× (e.g., 0.5 to 0.1 mL) to match the enrichment factor applied to transport samples. Test compound concentrations were determined by HPLC using the same chromatographic method described above, with and an injection volume of 20 μL. Quantification was performed by comparing the analyte AUC in AP and BL samples with the processed 20 μM control (AUC ratios), considering the linear detector response under these conditions. Apparent permeability coefficients (*P*_app_) were estimated from the 120 min end point as *P*_app_ ≈ *n*_BL_/(*A*·*C*_0_·*t*), where *A* = 0.33 cm^2^, *C*_0_ = 20 μM, and *t* = 7200 s. Mass recovery (mass balance) was calculated as % Recovery = 100 × (*n*_AP,end_ + *n*_BL_)/*n*_AP,0_, where *n*_AP,0_ = *C*_0_·*V*_AP_ and *V*_AP_ = 0.5 mL. Data are reported as mean ± SD (*n* = 2).

### 4.6 Cell Cycle Analysis and Apoptosis Assay

U937 cells were maintained in RPMI-1640 medium supplemented with 10% bovine serum (FBS, Euroclone Milan IT), 2 mM l-glutamine, 100 U/mL penicillin, and 100 μg/mL streptomycin (Euroclone) at 37 °C in a humidified atmosphere containing 5% CO_2_. Cells were seeded at a density of 2 × 10^5^ cells/mL and treated with the following compounds: SAHA (1 μM), **14a** (0.125 and 0.25 μM), and **14b** (0.125 and 0.25 μM). Control cells were treated with an equivalent volume of the vehicle (0.5% (*v/v*) DMSO). Cells were harvested after 24 and 48 h of treatment for subsequent analyses.

For cell cycle analysis, 1 × 10^6^ cells were harvested after treatment, washed twice with PBS, and fixed in 70% cold ethanol at −20 °C overnight. Fixed cells were washed twice with PBS and incubated with a mix containing RNase A (100 μg/mL) and PI (50 μg/mL) for 30 min at room temperature in the dark. The DNA content was analyzed by flow cytometry using a FACSCalibur flow cytometer (BD Biosciences). For each sample, 20,000 events were collected. The percentages of cells in sub-G1 (apoptotic cells) were determined using Flowing software.

Apoptosis was evaluated using the Annexin V-FITC/Propidium Iodide (PI) double staining method by using Annexin V-FITC apoptosis detection kit (Enzo Life science). Briefly, after treatment, cells were harvested, washed with PBS, and resuspended in 100 μL of 1× binding buffer. Cells were then incubated with 5 μL of Annexin V-FITC and 5 μL of PI for 15 min at room temperature in the dark. After incubation, 600 μL of 1× binding buffer was added to each sample, and cells were analyzed by flow cytometry using a FACSCalibur flow cytometer (BD Biosciences). For each sample, at least 10,000 events were collected. The percentages of viable cells (Annexin V^–^/PI^–^), early apoptotic cells (Annexin V^+^/PI^–^), late apoptotic cells (Annexin V^+^/PI^+^), and necrotic cells (Annexin V^–^/PI^+^) were determined using Kaluza software (Beckman Coulter).

All experiments were performed in triplicate, and data are presented as mean ± standard deviation (SD). Statistical significance was determined using two-way analysis of variance (ANOVA) followed by Tukey’s posthoc test. GraphPad Prism 8 software was used for all statistical analyses and graph generation.

### 4.7 Histone Extraction

Cells were seeded at a density of 2 × 10^5^ per mL and treated with the compounds for 48 h. Then, cells were collected and washed two times with ice-cold PBS. Samples were resuspended in Triton extraction buffer (TEB; PBS containing 0.5% Triton X-100 (v/v), 2 mM phenylmethylsulfonylfluoride (PMSF), and 0.02% (w/v) NaN_3_) and washed for 10 min at 4 °C with gentle stirring. Next, samples were centrifuged at 2000 rpm for 10 min at 4 °C, and the pellets were washed in TEB (half of the volume) and centrifuged in the same conditions. Samples were resuspended in 0.2 N HCl, and histones were extracted overnight at 4 °C with gentle stirring. Samples were centrifuged at 2000 rpm for 10 min at 4 °C. Histone concentrations were determined by Bradford assay (Bio-Rad).

### 4.8 Total Protein Extraction and Western Blotting

For total protein extraction, U937 cells were harvested and washed two times with ice-cold PBS and lysed in ice-cold lysis buffer (50 mM Tris·HCl pH 7.4, 150 mM NaCl, 1% NP40, 10 mM NaF, 1 mM PMSF and protease inhibitor cocktail). The lysates were centrifuged at 12,000 rpm for 15 min at 4 °C. Protein concentrations were determined by Bradford assay (Bio-Rad).

For WB analysis, 30 μg of total protein extract and 3 μg of histone extracts were denatured and boiled in sample buffer Laemmli (0.25 M Tris·HCl, pH 6.8, 8% SDS, 40% glycerol, 5% 2-mercaptoethanol, and 0.05% bromophenol blue) for 3 min and then separated on polyacrylamide gels. After electrophoresis, total and histone proteins were transferred to nitrocellulose membranes (Bio-Rad mini-protean gel and Transblot Turbo transfer system, Bio-Rad). Membranes were blocked at room temperature for 1 h in 5% nonfat milk and then incubated overnight at 4 °C with the appropriate primary antibodies. Antimouse or antirabbit immunoglobulin G (IgG)–horseradish peroxidase (HRP)-conjugated antibodies were used as secondary antibodies at 1:5000 dilution for 1 h at room temperature. According to the manufacturer’s specification, antibody binding was visualized by enhanced chemiluminescence and recorded on autoradiography film.

Antibodies used in experiments with U937 cells were: α-Tubulin (Santa Cruz sc-32293, mouse monoclonal); Acetylated-Tubulin (Sigma-Aldrich T 6793, mouse monoclonal); Cyclin D1 (Cell Signaling Technology 2978S, Rabbit monoclonal); histone H3 (Nouvs Biologicals NB500171, Rabbit polyclonal); Acetyl-Histone H3 Lys9 (Cell signaling technology 9649, Rabbit monoclonal); HSP90 (Santa Cruz sc-13119, mouse monoclonal). HSP90 and histone H3 were used as equal loading of total and histone proteins, respectively.

*T*otal protein extracts from PC-3M-luc2 and 22Rv1-luc cells were obtained using USA buffer as in ref 66. Briefly, after incubation on ice for 15 min, they were sonicated for 10 min and centrifuged at 14,000 rpm for 20 min at 4 °C. Protein concentration was measured using the Bradford Protein Assay according to manufacturer’s instruction Western blots were performed using 20 μg of protein extract resolved by 4–12% gradient Invitrogen Precast gel (MES buffer) and revealed with an ECL Western Blot Detection Kit (Amersham Pharmacia Biotech, Buckinghamshire, England) using UVIDOC (Eppendorf, Hamburg, Germany). Densitometry analysis was performed with ImageJ software (version 1.8.0).

Antibodies used in experiments with PC-3M-luc2 and 22Rv1-luc cells were: anti Histone H3 96C10 Cell signaling (#3638), anti Histone H3 (acetyl K9) abcam (#ab10812), Acetyl-α-Tubulin (Lys40) Invitrogen (#32-2700), Bcl-2 R&D system (#AF810), p21 WAF1/Cip1 (12D1) Cell Signaling (#2947), β-actin Abcam (#ab8227), Donkey anti-Goat IgG HRP Abcam (#ab6885), Goat anti-Mouse IgG HRP Bio-Rad Cat (#170-6516), Goat-anti-Rabbit IgG HRP SeraCare KPL (#5220–0336).

### 4.9 mRNA and miRNAs Extraction and Reverse-Transcriptase Polymerase Chain Reaction

mRNAs and miRNAs were extracted from cell cultures with miRNeasy Mini Kit (QIAGEN Cat. 217004). MiRNAs were reverse transcribed with Mir-X miRNA First-Strand Synthesis Kit (Cat. 638313) according to the manufacturer’s instructions. mRNAs were reverse transcribed with Takara Prime Script RT Mastermix (RR036A-1) from Takara (Kusatsu, Japan). cDNAs were amplified by qPCR reaction using GoTaq qPCR Master Mix (Promega, Madison, WI, USA), and the reaction was carried out in a BioRad-iQ-iCycler. The results were analyzed with CFX Manager software (Biorad), and the relative amounts obtained with 2­(−Δ*Ct*) method were normalized with respect to the gene L34 and miR-16. Statistical significance was determined with a *t* test (one tailed) using GraphPad Prism version 8.0 (La Jolla, CA, USA). Differences were considered significant *: *P* < 0.05; ** *P* < 0.01; *** *P* < 0.001.

## Supplementary Material





## Data Availability

Data will be made available upon request.

## Notes

Elemental analyses for final compounds **3a**,**b–13a**,**b**, **15a**,**b**, **17a**,**b–27** (Table S1); HDAC inhibitory activity and chemical-physical parameters of final compounds **1**–**27** (Table S2); Caco-2 intestinal permeability of compounds **14a** and **14b** (Table S3); purity control by HPLC of compounds **8a**, **14a**, **14b**, **16a**, and **27**; HPLC traces of compounds **8a**, **14a**, **14b**, **16a**, **27** (Figures S1–S5); HPLC traces of compound **14a** and **14b** in PBS (pH 7.4) after 0, 48, and 72 h (Figures S6–S7); Caco-2 transwell transport of compounds **14a** and **14b** and quantification in the basolateral (BL) compartment (Figure S8); effect of **14a** and **14b** on the TEER of differentiated Caco-2 cell monolayers (Figure S9); and uncropped Western blots (PDF)
Molecular formula strings (CSV)

## Abbreviations Used

AP: apical
BL: basolateral
CC_50_: concentration required to reduce cell viability by 50%
CU: connecting unit
EMA: European Medicines Agency
EMT: epithelial–mesenchymal transition
GI_50_: half-maximal growth inhibition
HDACi: HDAC inhibitor
miRNA: microRNA
MTT: 3-(4,5-dimethylthiazol-2-yl)-2,5-diphenyltetrazolium bromide
NMPA: Chinese National Medical Products Administration
UBHAs: Uracil-based hydroxamic acids
WB: Western blot
ZBG: zinc-binding group (ZBG)
