# Supplementary material for: Development of Biphenyl-Substituted Uracil-Based Hydroxamic Acids (UBHAs) as Potent HDAC Inhibitors with Pro-Apoptotic Activity in Leukemia and Prostate Cancer Cells
Source: J Med Chem. 2026 Apr 30;69(9):10060–82. doi: 10.1021/acs.jmedchem.5c02737 (PMC13181782; doi:10.1021/acs.jmedchem.5c02737)
Supplement: Supplementary file 1 [file jm5c02737_si_001.pdf]

Supporting Information

Development of Biphenyl-Substituted Uracil-Based Hydroxamic Acids (UBHAs) as potent HDAC Inhibitors with Pro-Apoptotic Activity in Leukaemia and Prostate Cancer Cells

Francesco Fiorentino[§1], Giulio Bontempi[§2, 3], Federica Michetti[2, 3], Valeria Pecci[4], Emanuele Fabbrizi[5], Daniela Passeri[6], Letizia Corsetti[7], Valentina Farini[8], Fabrizio Casano[5], Antimo Gioiello[9], Antonella Di Sotto[7], Roberto Pellicciari[6], Donatella Del Bufalo[8], Daniela Trisciuoglio[10], Simona Nanni[4,11], Raffaele Strippoli[2,3,*], Antonello Mai[5,*], Dante Rotili[12,13*]

[1] *Department of Biochemical Sciences, Sapienza University of Rome, Piazzale Aldo 5, 00185, Rome, Italy.*

[2] *Department of Molecular Medicine, Sapienza University of Rome, Piazzale Aldo Moro 5, 00185, Rome, Italy*

[3] *Gene Expression Laboratory, National Institute for Infectious Diseases, Lazzaro Spallanzani IRCCS, Via Portuense, 292, 00149, Rome, Italy*

[4] *Department of Translational Medicine and Surgery, Università Cattolica del Sacro Cuore, Largo Francesco Vito, 1, 00168 Rome, Italy.*

[5] *Department of Drug Chemistry and Technologies, Sapienza University of Rome, Piazzale Aldo 5, 00185, Rome, Italy.*

[6] *TES Pharma S.r.l., Via P. Togliatti 20, Corciano, 06073, Perugia, Italy.*

[7] *Department of Physiology and Pharmacology "V. Erspamer", Sapienza University of Rome, Piazzale Aldo Moro 5, 00185, Rome, Italy.*

[8] *Preclinical Models and New Therapeutic Agents Unit, IRCCS-Regina Elena National Cancer Institute, Rome 00144, Italy.*

[9] *Department of Pharmaceutical Sciences, University of Perugia, Via del Liceo 1, 06122 Perugia, Italy.*

[10] *Institute of Molecular Biology and Pathology, National Research Council (CNR), Via degli Apuli, 4, Rome 00185, Italy.*

[11] *Fondazione "Policlinico Universitario A. Gemelli IRCCS", Largo Francesco Vito, 1, 00168 Rome, Italy.*

[12] *Department of Science, Roma Tre University, Viale Marconi 446, 00146, Rome, Italy.*

[13] *Biostructures and Biosystems National Institute (INBB), Via dei Carpegna 19, 00165, Rome, Italy.*

[§] *These authors contributed equally to this work*

[*] ***Correspondence**: Dante Rotili (dante.rotili@uniroma3.it), Antonello Mai (antonello.mai@uniroma1.it), Raffaele Strippoli (raffaele.strippoli@uniroma1.it)*

Contents	Page(s):
**Table S1.** Elemental analyses for final compounds **3a,b – 13a,b, 15a,b, 17a,b – 27.**	S3
**Table S2.** HDAC Inhibitory activity and chemical-physical parameters of final compounds **1-27**.	S5
**Table S3.** Caco-2 Intestinal Permeability of compounds **14a** and **14b**.	S8
**Purity control by HPLC of compounds 8a, 14a, 14b, 16a, and 27.**	S9
**Supplementary Figure S1.** HPLC trace of compound **8a**.	S9
**Supplementary Figure S2.** HPLC trace of compound **14a**.	S10
**Supplementary Figure S3.** HPLC trace of compound **14b**.	S10
**Supplementary Figure S4.** HPLC trace of compound **16a**.	S11
**Supplementary Figure S5.** HPLC trace of compound **27**.	S11
**Supplementary Figure S6.** HPLC traces of compound **14a** in PBS (pH 7.4) after 0, 48, and 72 h.	S12
**Supplementary Figure S7.** HPLC traces of compound **14b** in PBS (pH 7.4) after 0, 48, and 72 h.	S12
**Supplementary Figure S8.** Caco-2 transwell transport of compounds **14a** and **14b** and quantification in the basolateral (BL) compartment.	S13
**Supplementary Figure S9.** Effect of compounds **14a** and **14b** (20 µM each) on the transepithelial electrical resistance (TEER) of differentiated Caco-2 cell monolayers after 2h incubation.	S14
**Uncropped western blots.**	S15

Table S1. Elemental analyses for final compounds **3a,b – 13a,b, 15a,b, 17a,b – 27.**												
**Cpd**	**Lab Code**	**MW**	**Calculated, %**					**Found, %**				
			**C**	**H**	**N**	**F/Cl**	**S**	**C**	**H**	**N**	**F/Cl**	**S**
**3a**	MC1736	333.406	57.64	5.74	12.60	-	9.62	57.75	5.76	12.55	-	9.60
**3b**	MC1732	347.433	58.77	6.09	12.09	-	9.23	58.83	6.11	12.03	-	9.21
**4a**	MC1730	333.406	57.64	5.74	12.60	-	9.62	57.71	5.76	12.54	-	9.61
**4b**	MC1729	347.433	58.77	6.09	12.09	-	9.23	58.85	6.11	12.03	-	9.21
**5a**	MC1725	333.406	57.64	5.74	12.60	-	9.62	57.75	5.76	12.56	-	9.60
**5b**	MC1726	347.433	58.77	6.09	12.09	-	9.23	58.83	6.11	12.04	-	9.22
**6a**	MC1723	353.821	50.92	4.56	11.88	10.02	9.06	50.99	4.58	11.82	10.01	9.04
**6b**	MC1724	367.848	52.24	4.93	11.42	9.64	8.72	52.31	4.95	11.36	9.62	8.71
**7a**	MC1716	353.821	50.92	4.56	11.88	10.02	9.06	50.99	4.58	11.82	10.00	9.05
**7b**	MC1717	367.848	52.24	4.93	11.42	9.64	8.72	52.3	4.95	11.38	9.63	8.71
**8a**	MC1714	353.821	50.92	4.56	11.88	10.02	9.06	51.03	4.58	11.83	10.00	9.04
**8b**	MC1715	367.848	52.24	4.93	11.42	9.64	8.72	52.31	4.95	11.37	9.63	8.70
**9a**	MC1910	337.369	53.4	4.78	12.46	5.63	9.5	53.5	4.8	12.41	5.61	9.48
**9b**	MC1911	351.396	54.69	5.16	11.96	5.41	9.12	54.77	5.18	11.9	5.4	9.10
**10a**	MC1887	337.369	53.4	4.78	12.46	5.63	9.50	53.49	4.8	12.42	5.62	9.49
**10b**	MC1888	351.396	54.69	5.16	11.96	5.16	9.12	54.76	5.18	11.91	5.15	9.10
**11a**	MC1820	349.405	55.0	5.48	12.03	-	9.18	55.09	5.5	11.98	-	9.16
**11b**	MC1819	363.432	56.18	5.82	11.56	-	8.82	56.28	5.84	11.52	-	8.80
**12a**	MC1821	349.405	55.0	5.48	12.03	-	9.18	55.1	5.5	11.97	-	9.17
**12b**	MC1818	363.432	56.18	5.82	11.56	-	8.82	56.27	5.84	11.51	-	8.81
**13a**	MC1930	395.477	63.78	5.35	10.63	-	8.11	63.88	5.37	10.58	-	8.09
**13b**	MC1931	409.504	64.53	5.66	10.26	-	7.83	64.62	5.68	10.20	-	7.82
**15a**	MC1739	369.439	61.77	5.18	11.37	-	8.68	61.85	5.2	11.33	-	8.66
**15b**	MC1737	383.466	62.64	5.52	10.96	-	8.36	62.73	5.54	10.91	-	8.34
**17a**	MC1850	325.427	55.36	7.12	12.91	-	9.85	55.44	7.14	12.87	-	9.84
**17b**	MC1851	339.454	56.61	7.42	12.38	-	9.44	56.69	7.44	12.34	-	9.43
**18**	MC1852	361.460	59.81	6.41	11.63	-	8.87	59.88	6.43	11.57	-	8.86
**19**	MC1849	361.460	59.81	6.41	11.63	-	8.87	59.88	6.43	11.59	-	8.86

**20**	MC1848	361.460	59.81	6.41	11.63	-	8.87	59.89	6.43	11.57	-	8.85
**21**	MC1847	381.875	53.47	5.28	11.00	9.28	8.4	53.57	5.30	10.95	9.27	8.39
**22**	MC1840	381.875	53.47	5.28	11.00	9.28	8.4	53.55	5.30	10.96	9.26	8.39
**23**	MC1846	381.875	53.47	5.28	11.00	9.28	8.4	53.56	5.30	10.95	9.26	8.38
**24**	MC1867	423.531	65.23	5.95	9.92	-	7.57	65.33	5.97	9.87	-	7.55
**25**	MC1861	397.493	63.46	5.83	10.57	-	8.07	63.57	5.85	10.51	-	8.06
**26**	MC1866	397.493	63.46	5.83	10.57	-	8.07	63.54	5.85	10.52	-	8.05
**27**	MC2026	377.444	70.01	6.14	11.13	-	12.72	70.12	6.16	11.07	-	12.71

Table S2. HDAC Inhibitory activity and chemical-physical parameters of compounds **1-27**.[a]

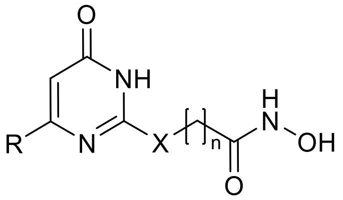

Cpd	Lab code	R	X	n	IC_{50}[b] (nM)					Calcd. P_{app} (10^{-6} cm/s)[c]	LogD (pH 7.4)[c]
					HDAC1	HDAC3	HDAC4	HDAC6	HDAC8		
**1a**	**MC1641**	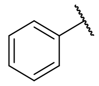	S	4	1310	1230	>100000	7.4	529	6.37	1.921
**1b**	**MC1650**	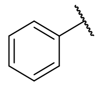	S	5	776	1260	93100	17.4	304	5.89	2.283
**2**	**MC1637**	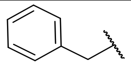	S	5	1210	1850	>100000	19.1	270	5.16	2.062
**3a**	**MC1736**	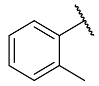	S	4	2980	4190	>100000	10.9	228	6.68	2.480
**3b**	**MC1732**	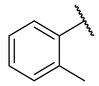	S	5	1710	3850	>100000	39.2	156	6.49	2.667
**4a**	**MC1730**	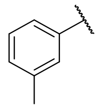	S	4	540	376	>100000	8.7	419	5.79	2.481
**4b**	**MC1729**	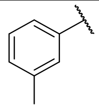	S	5	360	345	>100000	21.3	247	5.53	2.818
**5a**	**MC1725**	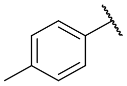	S	4	197	149	>100000	7.3	106	5.70	2.470
**5b**	**MC1726**	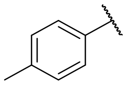	S	5	119	115	>100000	15.8	81.4	6.01	2.800
**6a**	**MC1723**	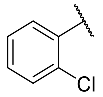	S	4	3020	5090	>100000	15.2	195	8.61	2.045
**6b**	**MC1724**	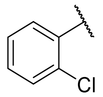	S	5	2130	5450	>100000	31.4	135	7.78	2.414
**7a**	**MC1716**	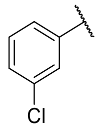	S	4	1650	540	>100000	9.5	1000	8.04	2.408
**7b**	**MC1717**	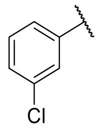	S	5	852	2260	57400	28.5	370	7.24	2.724

**8a**	**MC1714**	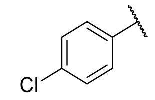	S	4	1220	468	>100000	4.1	743	8.15	2.449
**8b**	**MC1715**	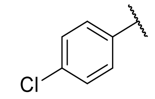	S	5	330	1990	>100000	22.0	229	7.38	2.748
**9a**	**MC1910**	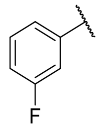	S	4	932	1530	>100000	7.6	1180	6.55	2.086
**9b**	**MC1911**	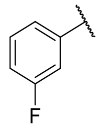	S	5	830	1620	>100000	21.4	510	6.01	2.444
**10a**	**MC1887**	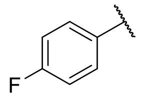	S	4	860	786	>100000	7.1	209	6.84	2.074
**10b**	**MC1888**	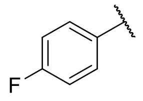	S	5	365	890	>100000	17.6	93.6	6.32	2.432
**11a**	**MC1820**	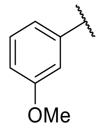	S	4	314	307	>100000	8.4	614	4.99	2.321
**11b**	**MC1819**	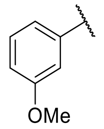	S	5	310	1040	>100000	31.3	188	4.83	2.626
**12a**	**MC1821**	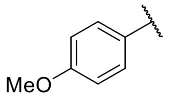	S	4	190	205	>100000	7.9	310	5.24	2.272
**12b**	**MC1818**	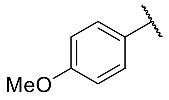	S	5	72.1	235	>100000	26.5	115	5.05	2.581
**13a**	**MC1930**	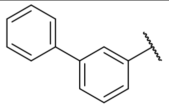	S	4	1510	1040	>100000	20.1	56.2	7.03	3.173
**13b**	**MC1931**	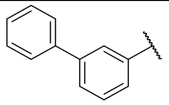	S	5	3250	3670	>100000	64.3	75.3	6.41	3.370
**14a**	**MC1742**	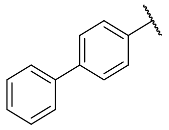	S	4	101	23.1	>100000	2.7	613	7.23	3.166
**14b**	**MC1738**	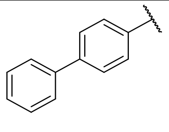	S	5	112	623	71300	4.9	1140	6.64	3.358
**15a**	**MC1739**	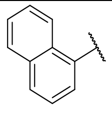	S	4	1150	908	>100000	8.9	533	8.67	2.807

**15b**	**MC1737**	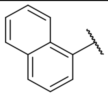	S	5	1390	1435	>100000	14.2	630	7.94	3.063
**16a**	**MC1745**	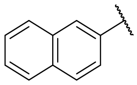	S	4	285	276	>100000	4.8	580	7.78	2.744
**16b**	**MC1746**	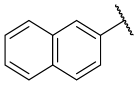	S	5	650	63.1	58100	9.7	610	7.10	3.041
**17a**	**MC1850**	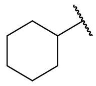	S	4	3550	4570	>100000	40.7	1620	5.85	2.200
**17b**	**MC1851**	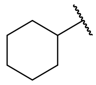	S	5	2100	3890	>100000	98.5	1310	5.16	2.584
**18**	**MC1852**	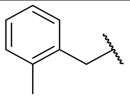	S	5	3240	4210	>100000	59.2	192	5.26	2.616
**19**	**MC1849**	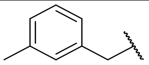	S	5	830	750	>100000	32.9	349	4.81	2.609
**20**	**MC1848**	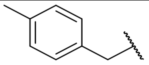	S	5	408	304	>100000	18.9	175	4.94	2.614
**21**	**MC1847**	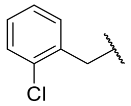	S	5	3780	6920	>100000	47.8	255	6.08	2.503
**22**	**MC1840**	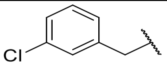	S	5	1800	2630	>100000	32.5	460	5.97	2.622
**23**	**MC1846**	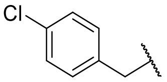	S	5	720	2210	>100000	29.3	242	5.98	2.654
**24**	**MC1867**	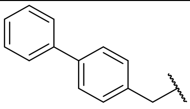	S	5	3250	6080	88700	171	1980	5.16	3.303
**25**	**MC1861**	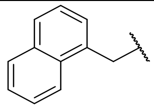	S	5	2190	2720	>100000	17.4	706	6.05	2.882
**26**	**MC1866**	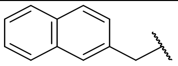	S	5	984	2940	>100000	13.9	721	5.87	2.849
**27**	**MC2026**	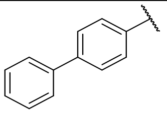	CH_2	4	2000	2840	>100000	4.9	2080	8.47	2.856
**SAHA**		-	-	-	260	350	490	30.0	240	10.7	1.575

[a] The compounds were tested against HDAC1, 3, 4, 6, 8 in a 10-dose IC_{50} mode with three-fold serial dilution starting from 100 μM solutions. [b] Inhibitory dose 50: dose required to inhibit the enzymatic activity by 50%.
[c] Calculated through ADMETlab2.0 (https://admetmesh.scbdd.com/)

**Table S3.** Caco-2 permeability of compounds **14a** and **14b**.[a]		
**Compound**	**P_{app} (cm/s)**	**Recovery (%)**
**14a**	$(1.17 \pm 0.03) \cdot 10^{-5}$	76 ± 4
**14b**	$(1.24 \pm 0.13) \cdot 10^{-5}$	78 ± 1

[a]Values are reported as mean ± standard deviation (SD).

Purity control by HPLC of compounds 8a, 14a, 14b, 16a, and 27.

The HPLC traces of compounds **8a**, **14a**, **14b**, **16a**, and **27** are sown below. The HPLC system consisted of a Dionex UltiMate 3000 UHPLC (Thermo Fisher) system equipped with an automatic injector, column heater and coupled with a Diode Array Detector DAD-3000 (Thermo Fisher). The analytical controls were performed on a Hypersil GOLD™ C18 Selectivity 5 µm (4.6 × 250 mm) HPLC Column (Thermo Fisher) in gradient elution. Eluents: A) H_2O/CH_3CN, 95/5 (v/v) + 0.1% TFA; B) CH_3CN/H_2O, 95/5 (v/v) + 0.1% TFA. The chromatographic run comprised 5 min at 10% solvent A, a subsequent 20 min linear gradient from 10% to 90% solvent B, and a final 5 min isocratic step at 90% solvent B. The flow rate was 1.0 mL/min, and the column was kept at a constant temperature of 30 °C. Samples were dissolved in solvent A at a concentration of 0.6 mg/mL and the injection volume was 1 µL.

By analysing the HPLC traces at 280 nm, a chemical purity > 95 % was recorded for all molecules:

i) 96.7% for compound **8a** (retention time 16.60 min);

ii) 97.3% for compound **14a** (retention time 17.50 min);

iii) 97.7% for compound **14b** (retention time 17.76 min);

iv) 96.6% for compound **16a** (retention time 17.21 min);

v) 96.4% for compound **27** (retention time 17.42 min).

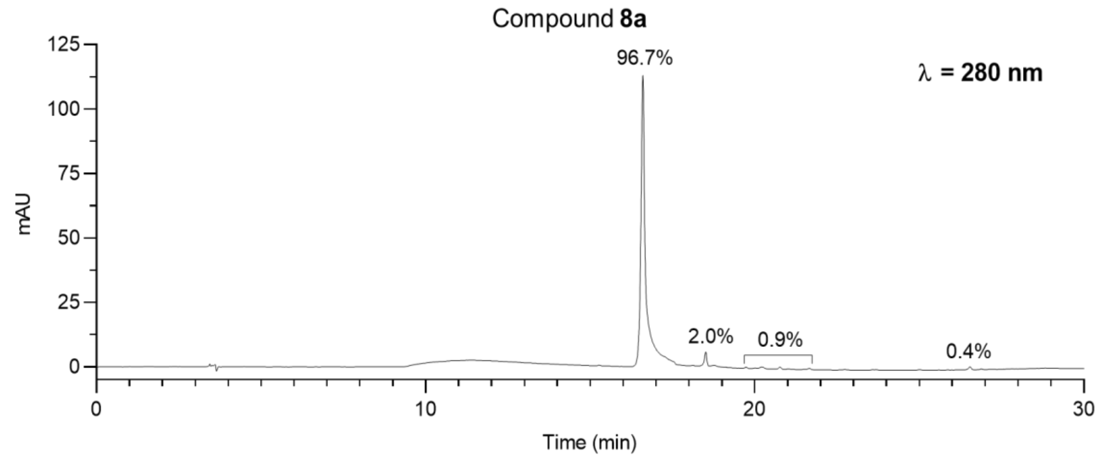

Supplementary Figure S1. HPLC trace of compound **8a**.

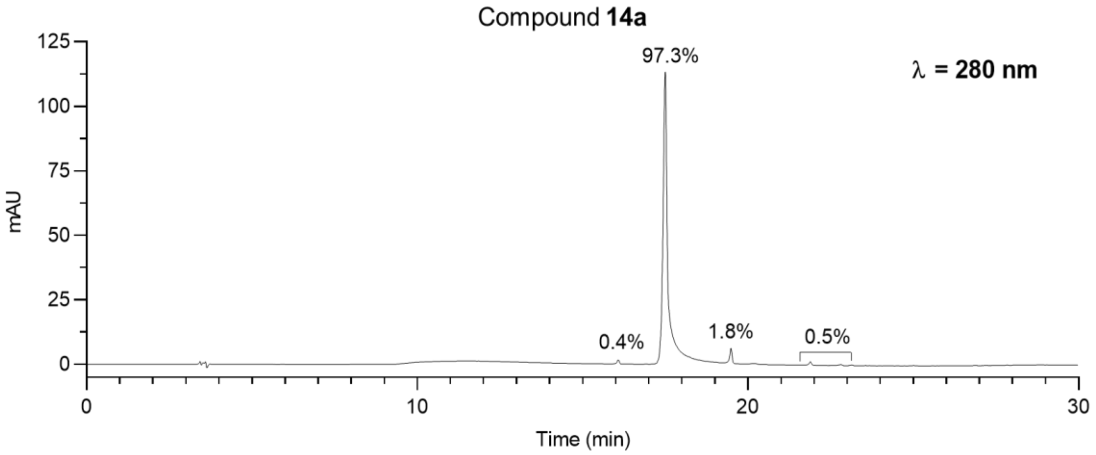

Supplementary Figure S2. HPLC trace of compound **14a**.

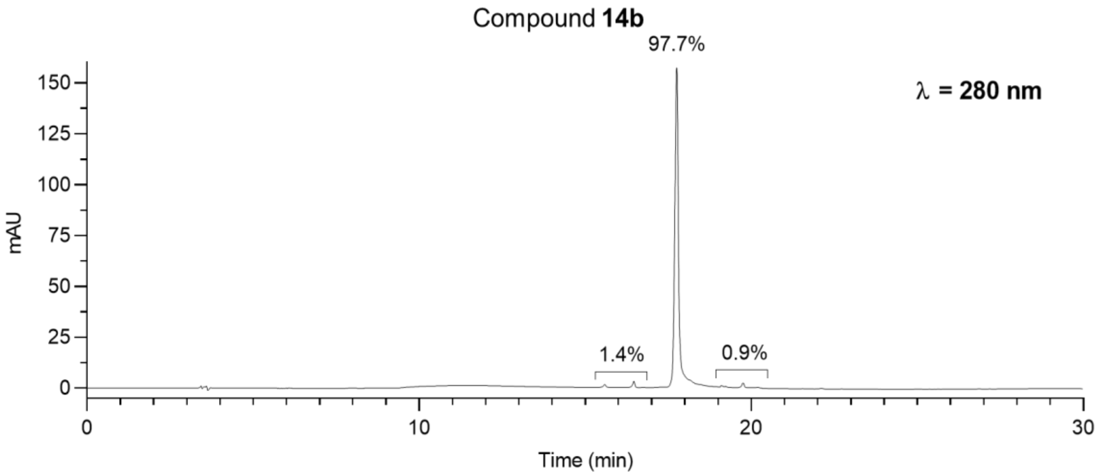

Supplementary Figure S3. HPLC trace of compound **14b**.

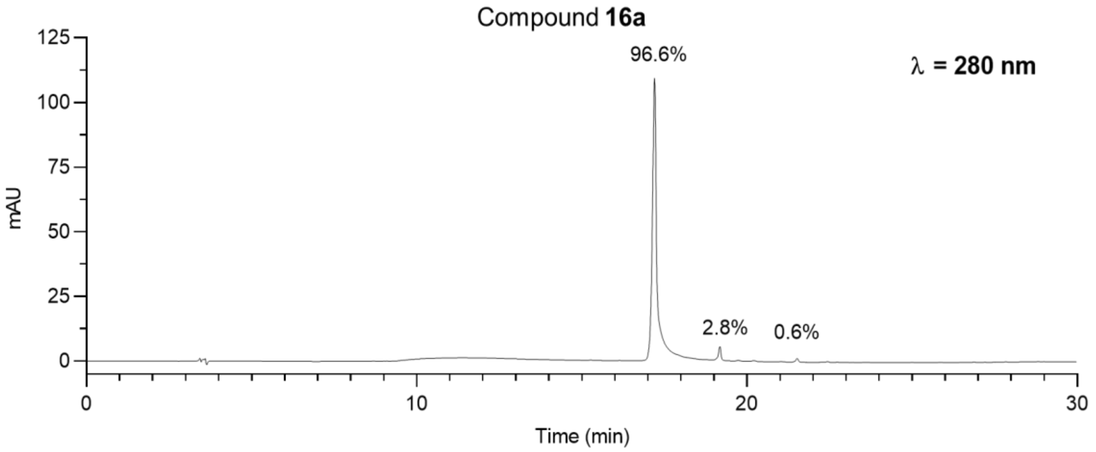

Supplementary Figure S4. HPLC trace of compound **16a**.

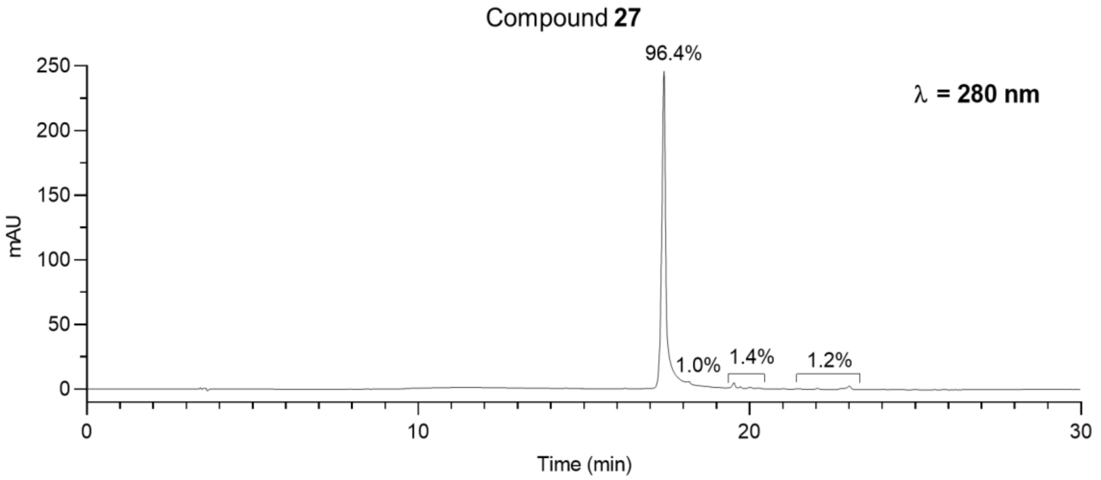

Supplementary Figure S5. HPLC trace of compound **27**.

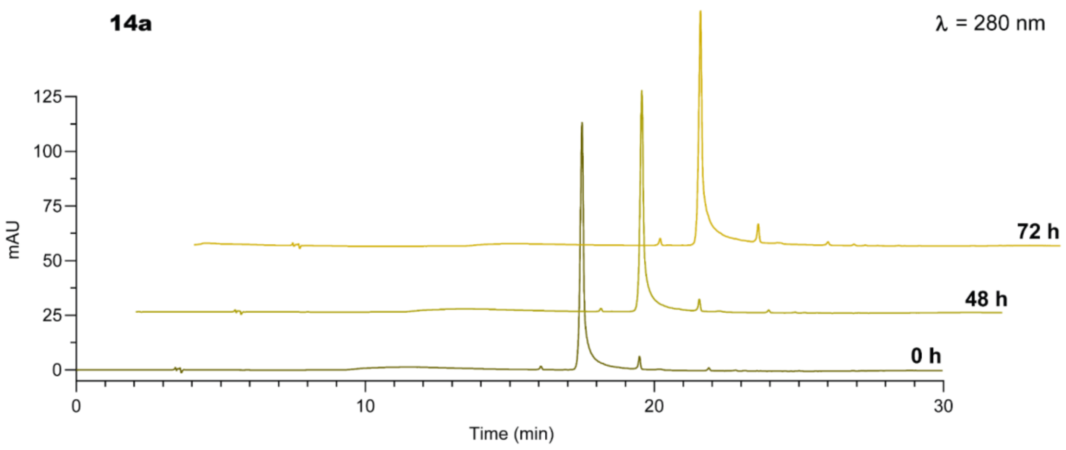

Supplementary Figure S6. HPLC traces of compound **14a** in PBS (pH 7.4) after 0, 48, and 72 h at 37 °C. Compound **14a** was diluted in PBS (c = 0.6 mg/ml) from a 30× DMSO stock. The solution was incubated at rt and then analyzed through analytical HPLC under the same conditions described in the Experimental Section. HPLC runs were performed after 0, 48, and 72 h of incubation and acquired at 280 nm.

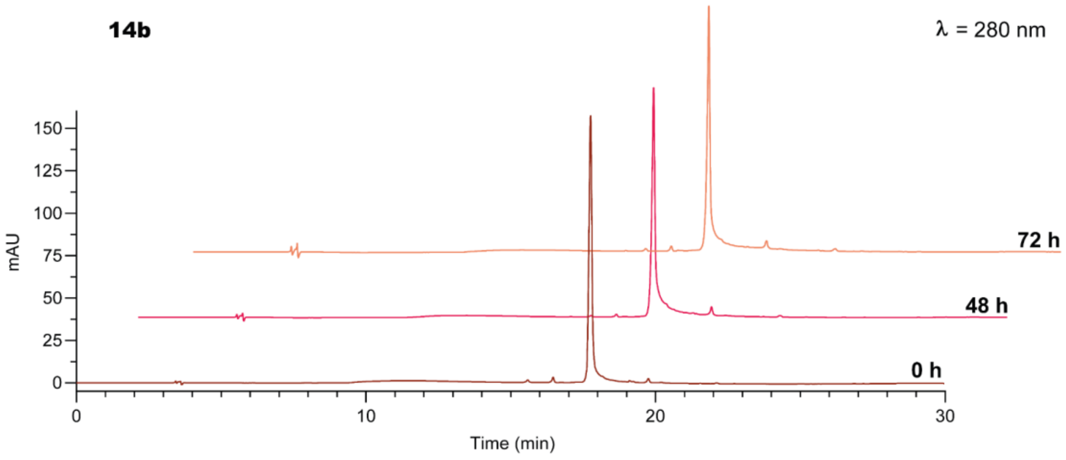

Supplementary Figure S7. HPLC traces of compound **14b** in PBS (pH 7.4) after 0, 48, and 72 h at 37 °C. Compound **14b** was diluted in PBS (c = 0.6 mg/ml) from a 30× DMSO stock. The solution was incubated at rt and then analyzed through analytical HPLC under the same conditions described in the Experimental Section. HPLC runs were performed after 0, 48, and 72 h of incubation and acquired at 280 nm.

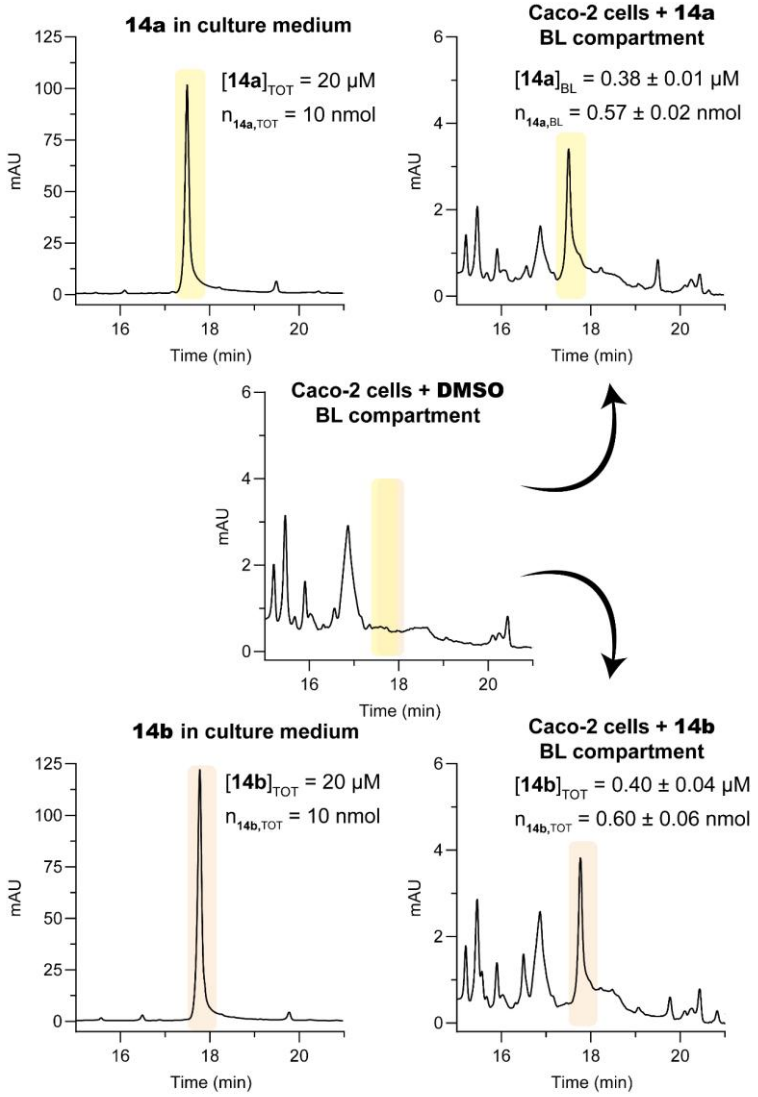

Supplementary Figure S8. Caco-2 Transwell transport of compounds **14a** and **14b** and quantification in the basolateral (BL) compartment. Representative HPLC chromatograms of **14a** and **14b** prepared in culture medium (20 µM; apical loading concentration; $n_{\mathrm{TOT}} = 10$ nmol in 0.5 mL) and of BL samples collected after 2 h incubation of Caco-2 monolayers with 20 µM compound (AP→BL direction). A BL sample from Caco-2 cells treated with vehicle (DMSO) is shown as a negative control. Highlighted regions indicate the retention-time window used for peak integration. BL concentrations were estimated from the AUC ratio relative to the processed 20 µM control (same sample preparation), and BL amounts were calculated as $n_{BL} = C_{BL} \cdot V_{BL}$ (with $V_{BL} = 1.5$mL). Values are reported as mean ± SD.

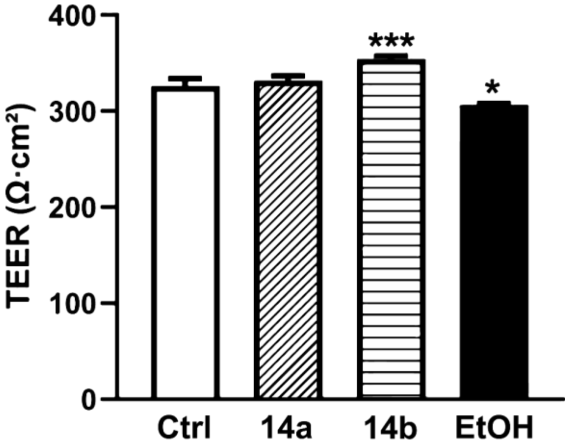

Supplementary Figure S9. Effect of compounds **14a** and **14b** (20 μM each) on the transepithelial electrical resistance (TEER) of differentiated Caco-2 cell monolayers after 2h incubation. TEER values were measured after treatment and expressed as Ω·cm². EtOH refers to treatment with ethanol at a concentration of 50% (v/v). Data are presented as mean ± SD of at least two independent experiments performed in triplicate. * $p < 0.05$ and *** $p < 0.001$, denote a significant difference with respect to control (one-way ANOVA, followed by Dunnett's multiple comparison test).

Uncropped western blots

FIG. 5A

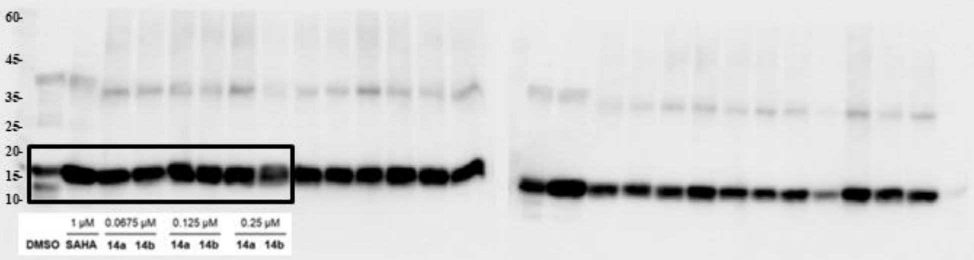

H3K9Ac n1

FIG. 5A

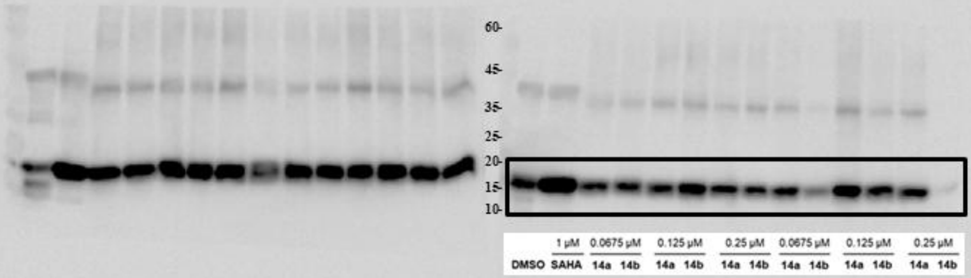

H3K9Ac n2 and n3

FIG. 5A

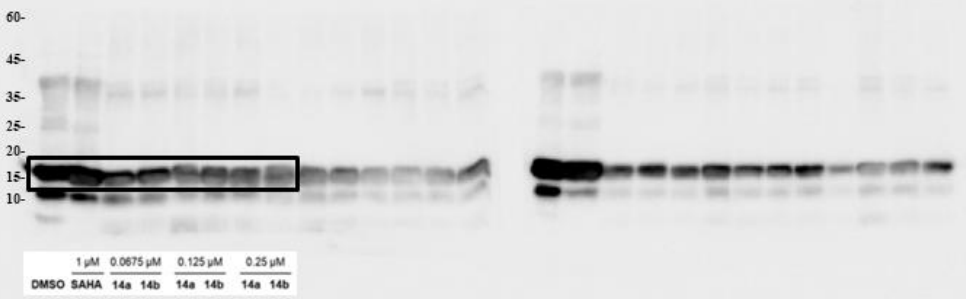
60-
45-
35-
25-
20-
15-
10-
1 μM
0.0675 μM
0.125 μM
0.25 μM
DMSO
SAHA
14a
14b
14a
14b
14a
14b

H3 n1

FIG. 5A

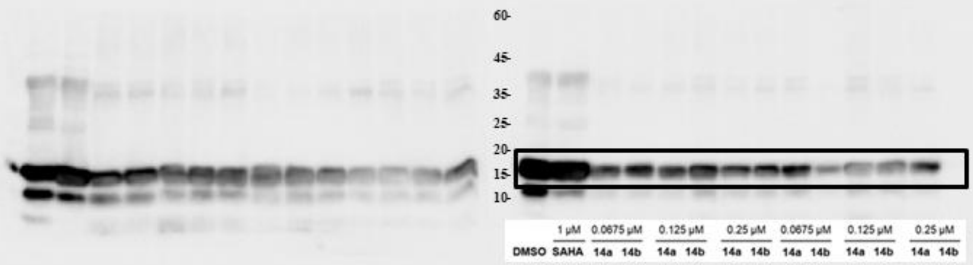

H3 n2 and n3

FIG. 5B

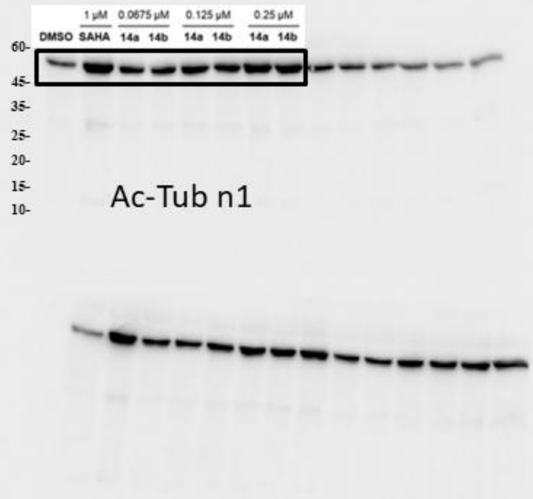
1 μM
0.0675 μM
0.125 μM
0.25 μM
DMSO
SAHA
14a
14b
14a
14b
14a
14b
60-
45-
35-
25-
20-
15-
10-
Ac-Tub n1

FIG. 5B

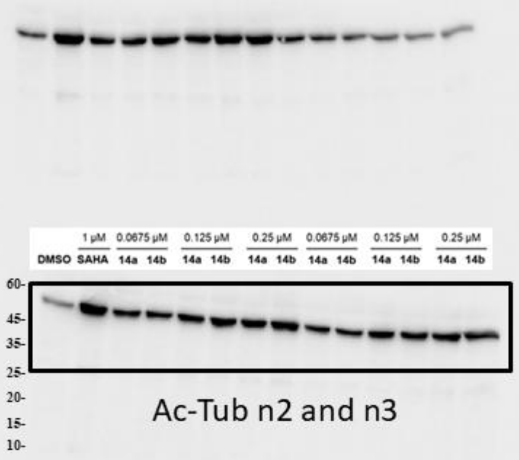
1 μM
0.0675 μM
0.125 μM
0.25 μM
0.0675 μM
0.125 μM
0.25 μM
DMSO
SAHA
14a
14b
14a
14b
14a
14b
14a
14b
14a
14b
14a
14b
60-
45-
35-
25-
20-
15-
10-
Ac-Tub n2 and n3

FIG. 5B

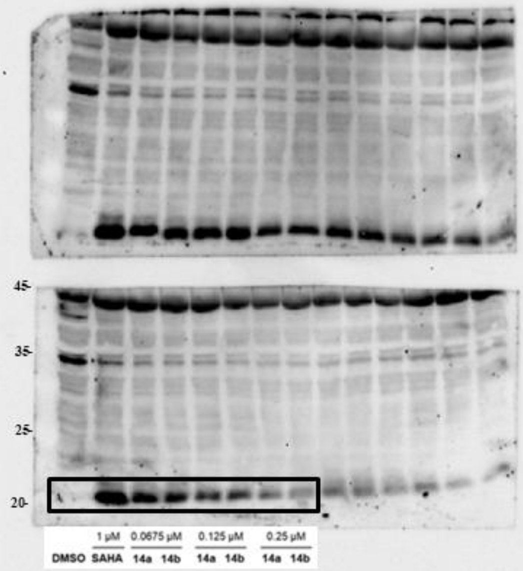
45
35
25
20
1 µM
0.0675 µM
0.125 µM
0.25 µM
DMSO SAHA 14a 14b 14a 14b 14a 14b

p21 n1

FIG. 5B

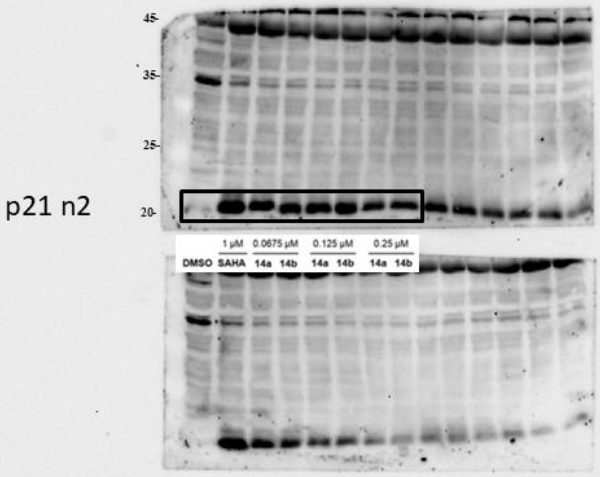
45-
35-
25-
20-
p21 n2
1 µM
0.0675 µM
0.125 µM
0.25 µM
DMSO SAHA 14a 14b 14a 14b 14a 14b

FIG. 5B

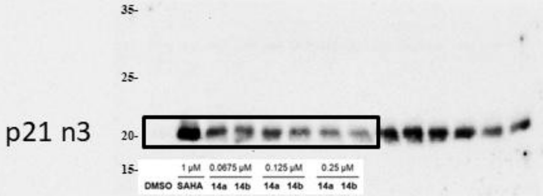
35-
25-
20-
15-
p21 n3
1 µM
0.0675 µM
0.125 µM
0.25 µM
DMSO SAHA 14a 14b 14a 14b 14a 14b

FIG. 5B

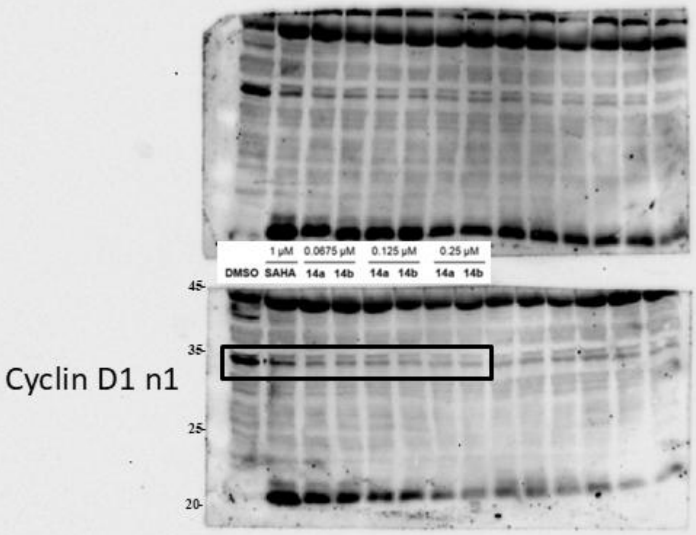
1 µM
0.0675 µM
0.125 µM
0.25 µM
DMSO
SAHA
14a
14b
14a
14b
14a
14b
45
35
25
20
Cyclin D1 n1

FIG. 5B

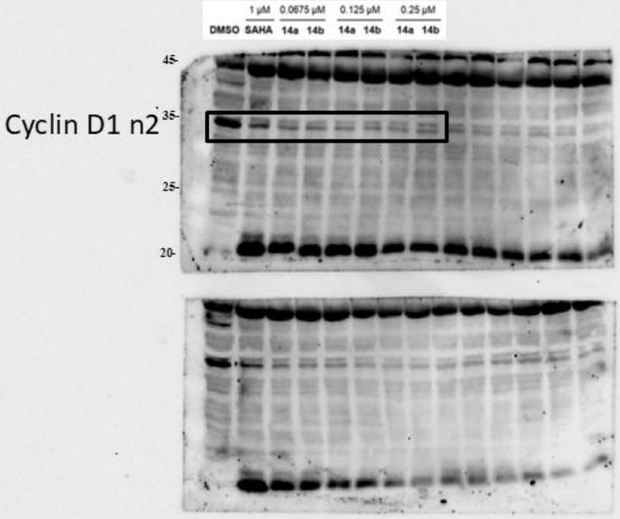
1 µM
0.0675 µM
0.125 µM
0.25 µM
DMSO
SAHA
14a
14b
14a
14b
14a
14b
45
35
25
20
Cyclin D1 n2

FIG. 5B

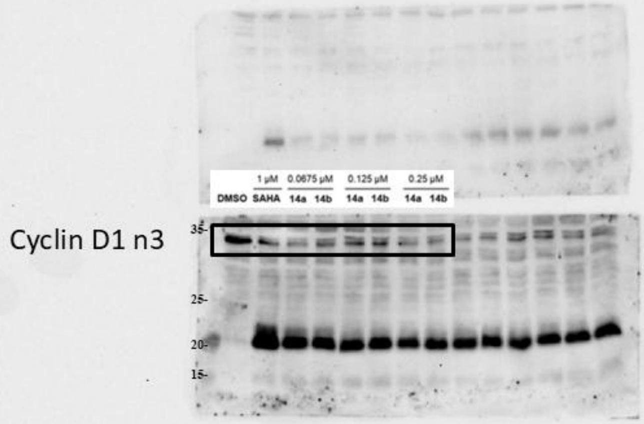

1 μM
0.0675 μM
0.125 μM
0.25 μM
DMSO
SAHA
14a
14b
14a
14b
14a
14b
Cyclin D1 n3
35-
25-
20-
15-

FIG. 5B

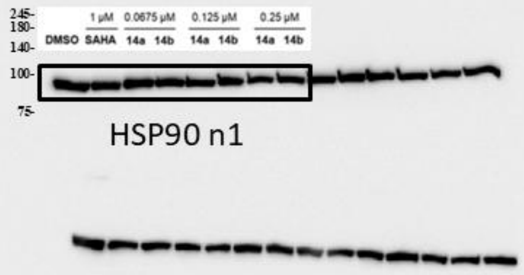
245-
180-
140-
100-
75-
1 μM
0.0675 μM
0.125 μM
0.25 μM
DMSO
SAHA
14a
14b
14a
14b
14a
14b
HSP90 n1

FIG. 5B

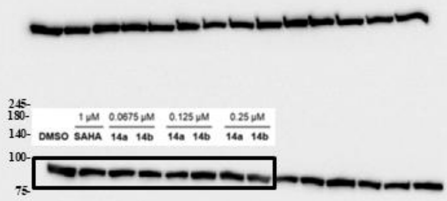

HSP90 n2

FIG. 5B

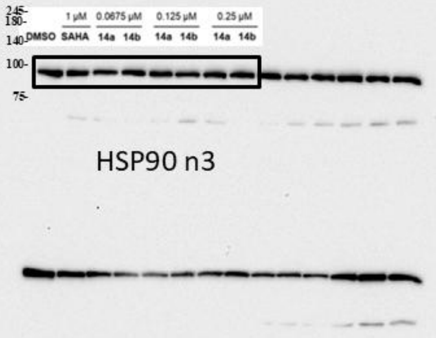

FIG. 8F

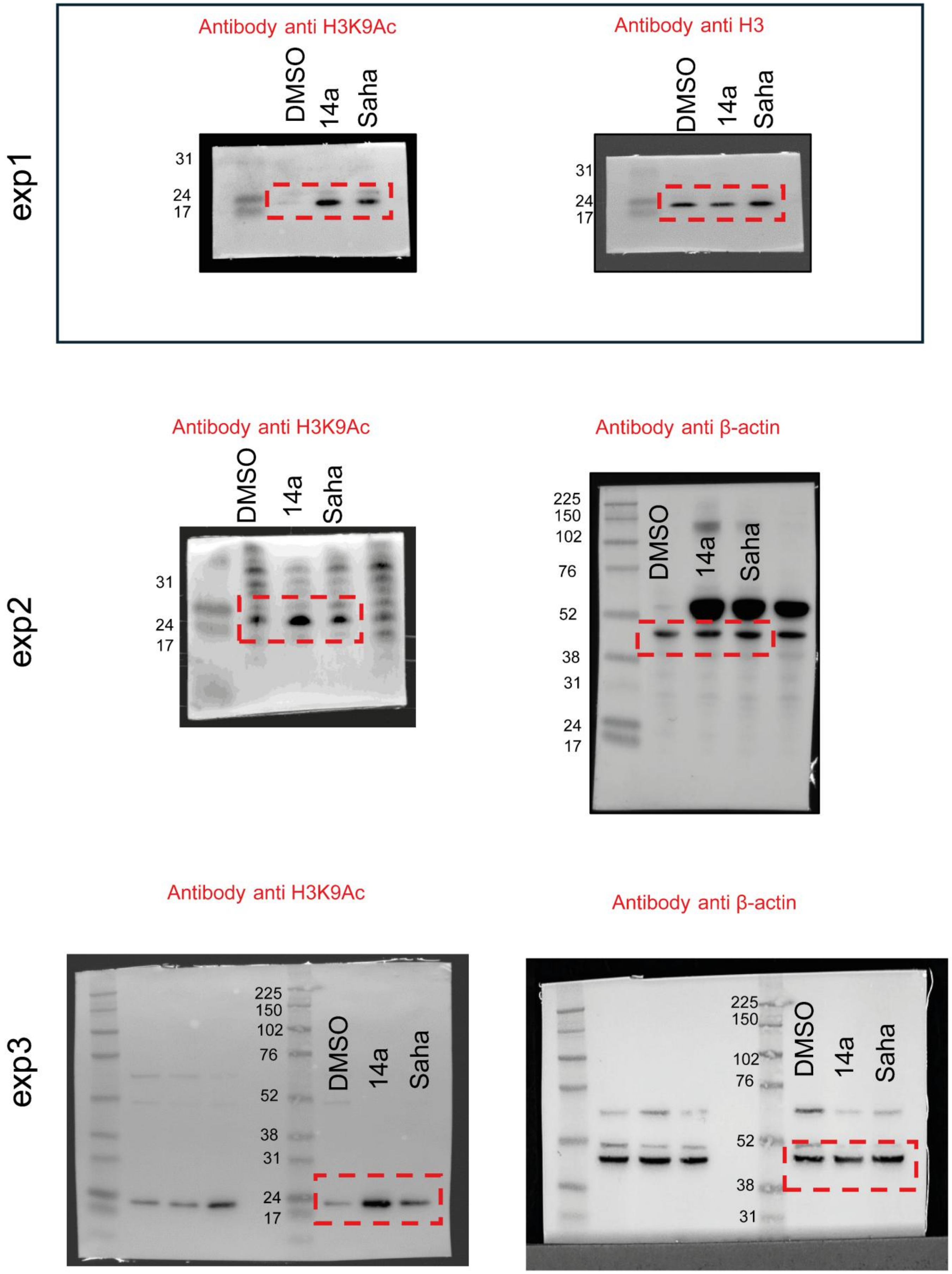
exp1
Antibody anti H3K9Ac
DMSO
14a
Saha
31
24
17
Antibody anti H3
DMSO
14a
Saha
31
24
17
exp2
Antibody anti H3K9Ac
DMSO
14a
Saha
31
24
17
Antibody anti β-actin
225
150
102
76
52
38
31
24
17
DMSO
14a
Saha
exp3
Antibody anti H3K9Ac
225
150
102
76
52
38
31
24
17
DMSO
14a
Saha
Antibody anti β-actin
225
150
102
76
52
38
31
DMSO
14a
Saha

FIG. 8G

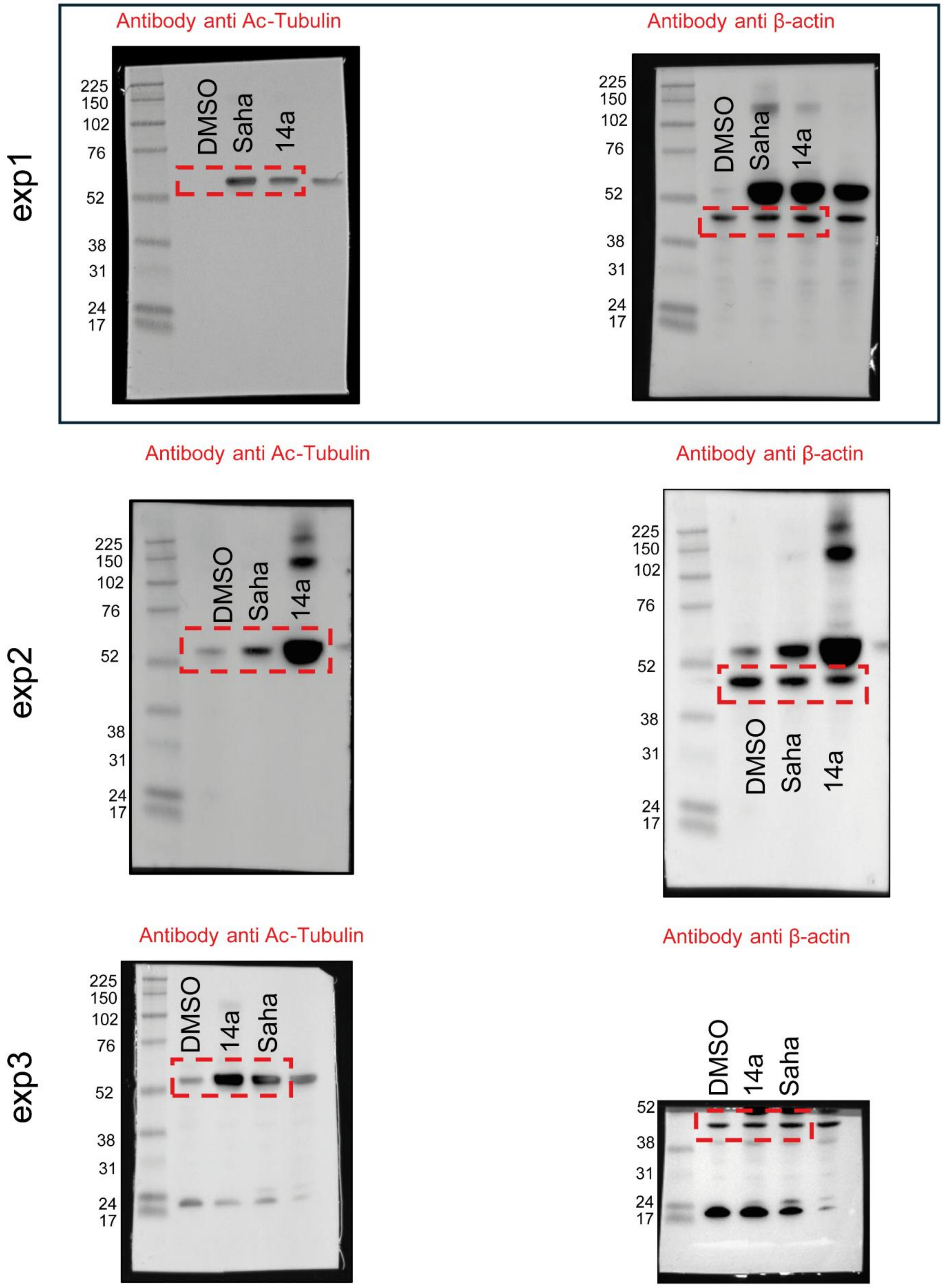

Antibody anti Ac-Tubulin
Antibody anti β-actin
exp1
225
150
102
76
52
38
31
24
17
DMSO
Saha
14a
Antibody anti Ac-Tubulin
Antibody anti β-actin
exp2
225
150
102
76
52
38
31
24
17
DMSO
Saha
14a
Antibody anti Ac-Tubulin
Antibody anti β-actin
exp3
225
150
102
76
52
38
31
24
17
DMSO
14a
Saha

FIG. 8G

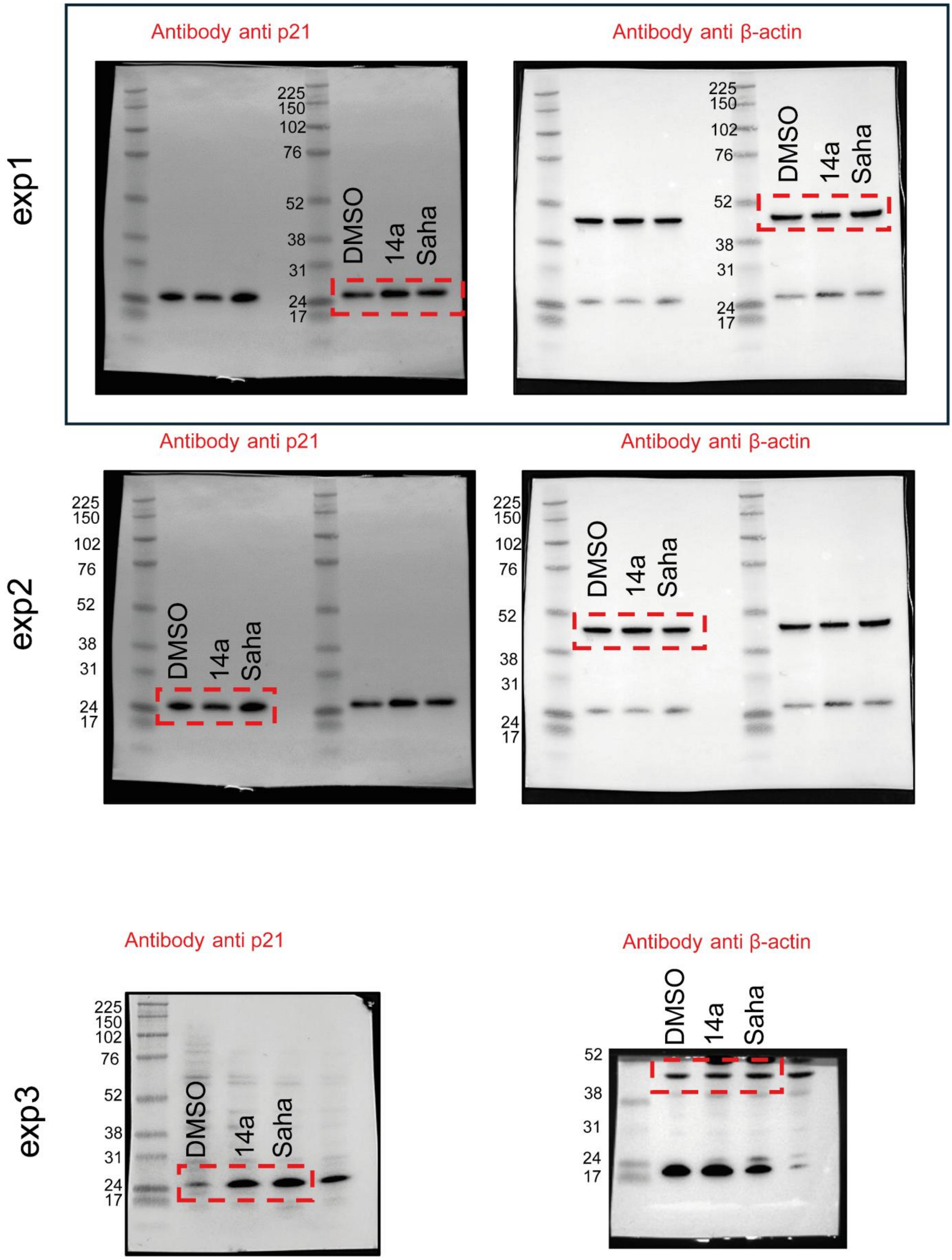
exp1
Antibody anti p21
225
150
102
76
52
38
31
24
17
DMSO
14a
Saha
Antibody anti β-actin
225
150
102
76
52
38
31
24
17
DMSO
14a
Saha
exp2
Antibody anti p21
225
150
102
76
52
38
31
24
17
DMSO
14a
Saha
Antibody anti β-actin
225
150
102
76
52
38
31
24
17
DMSO
14a
Saha
exp3
Antibody anti p21
225
150
102
76
52
38
31
24
17
DMSO
14a
Saha
Antibody anti β-actin
DMSO
14a
Saha
52
38
31
24
17

FIG. 8I

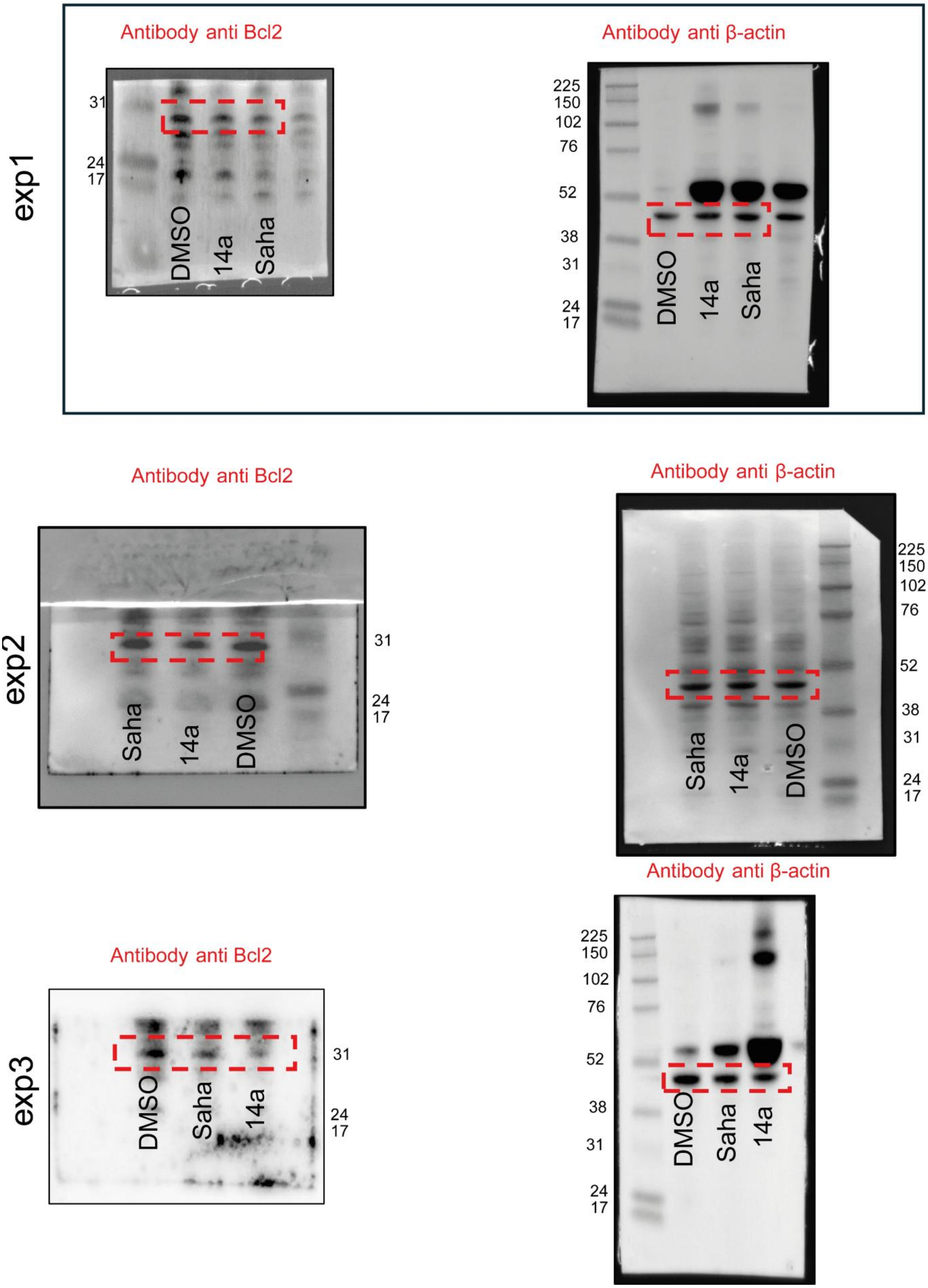

exp1
Antibody anti Bcl2
31
24
17
DMSO
14a
Saha
Antibody anti β-actin
225
150
102
76
52
38
31
24
17
DMSO
14a
Saha
exp2
Antibody anti Bcl2
31
24
17
Saha
14a
DMSO
Antibody anti β-actin
225
150
102
76
52
38
31
24
17
Saha
14a
DMSO
exp3
Antibody anti Bcl2
31
24
17
DMSO
Saha
14a
Antibody anti β-actin
225
150
102
76
52
38
31
24
17
DMSO
Saha
14a
